# EGFR-mediated local invasiveness and response to Cetuximab in head and neck cancer

**DOI:** 10.1186/s12943-025-02290-1

**Published:** 2025-03-22

**Authors:** Jiefu Zhou, Min He, Qiong Zhao, Enxian Shi, Hairong Wang, Vaidehi Ponkshe, Jiahang Song, Zhengquan Wu, Dongmei Ji, Gisela Kranz, Anna Tscherne, Sabina Schwenk-Zieger, Nilofer Abdul Razak, Julia Hess, Claus Belka, Horst Zitzelsberger, Iordanis Ourailidis, Fabian Stögbauer, Melanie Boxberg, Jan Budczies, Christoph A. Reichel, Martin Canis, Philipp Baumeister, Hongxia Wang, Kristian Unger, Andreas Mock, Olivier Gires

**Affiliations:** 1 Department of Otorhinolaryngology, LMU University Hospital, LMU Munich, Munich, Germany; 2 Department of Sports Medicine, Xiangya Hospital, Central South University, Xiangya Road 87, Changsha, 410008 China; 3 Key Laboratory of Organ Injury, Aging and Regenerative Medicine of Hunan Province, Xiangya Road 87, Changsha, 410008 China; 4 Hunan Engineering Research Center of Sports and Health, Changsha, 410008 China; 5 Department of Dermatology, The Third Affiliated Hospital of Sun Yat-Sen University, Guangzhou, 510630 Guangdong China; 6 Department of Medical Oncology, Department of Oncology, Fudan University Shanghai Cancer Center, Shanghai Medical College, Fudan University, Shanghai, 200032 China; 7 Research Unit Translational Metabolic Oncology, Institute for Diabetes and Cancer, Helmholtz Zentrum München, Deutsches Forschungszentrum Für Gesundheit Und Umwelt (GmbH), Neuherberg, Germany; 8 Department of Radiation Oncology, University Hospital, LMU Munich, Munich, Germany; 9 Bavarian Cancer Research Center (BZKF), Munich, Germany; 10 German Cancer Consortium (DKTK), Partner Site, Munich, Germany; 11 Comprehensive Cancer Center (CCC), Munich, Germany; 12 Institute of Pathology, University of Heidelberg, Im Neuenheimer Feld 224, 69120 Heidelberg, Germany; 13 Technical University of Munich, TUM School of Medicine and Health, Institute of General and Surgical Pathology, Munich, Germany; 14 Institute of Pathology, Faculty of Medicine, LMU Munich, Munich, Germany

**Keywords:** Oncogene addiction, EGFR, R/M-HNSCC, Cetuximab, EMT, Local invasion, fDEGs, Invasive gene regulatory network

## Abstract

**Background:**

Recurrent/metastatic head and neck squamous cell carcinoma (R/M-HNSCC) is a severe, frequently lethal condition. Oncogene addiction to epidermal growth factor receptor (EGFR) is a hallmark of HNSCC, but the clinical efficacy of EGFR-targeted therapies remains low. Understanding molecular networks governing EGFR-driven progression is paramount to the exploration of (co)-treatment targets and predictive markers.

**Methods:**

We performed function-based mapping of differentially expressed genes in EGFR-mediated local invasion (fDEGs) using photoconvertible tracers and RNA-sequencing (RNA-seq) in a cellular 3D-model.

**Results:**

Upon alignment with public single-cell RNA-seq (scRNA-seq) datasets and HNSCC-specific regulons, a gene regulatory network of local invasion (invGRN) was inferred from gene expression data, which was overrepresented in budding tumors. InvGRN comprises the central hubs inhibin subunit beta alpha (*INHBA*) and snail family transcriptional repressor 2 (*SNAI2*), and druggable fDEGs integrin subunit beta 4 (*ITGB4*), laminin 5 (*LAMB3*/*LAMC2*), and sphingosine kinase 1 (*SPHK1*). Blockade of INHBA repressed local invasion and was reverted by activin A, laminin 5, and sphingosine-1-phosphate, demonstrating a functional interconnectivity of the invGRN. Epithelial-to-mesenchymal transition (EMT) of malignant cells and the invGRN are induced by newly defined EGFR-activity subtypes with prognostic value that are promoted by amphiregulin (*AREG*) and epiregulin (*EREG*). Importantly, co-inhibition of SPHK1 showed synthetic effects on Cetuximab-mediated invasion blockade and high expression of selected fDEGs was associated with response to Cetuximab in patient-derived xenotransplantation (PDX) and R/M-HNSCC patients.

**Conclusions:**

We describe an actionable network of EGFR-mediated local invasion and define druggable effectors with predictive potential regarding the response of R/M-HNSCC to Cetuximab.

**Supplementary Information:**

The online version contains supplementary material available at 10.1186/s12943-025-02290-1.

## Background

Locally advanced HNSCC frequently progress to recurrent and/or metastatic HNSCC (R/M-HNSCC) with barely any means for cure and poor survival [1]. According to current treatment guidelines, choices of first-line therapy depend on programmed cell death-ligand 1 (PD-L1) expression. Tumors with a combined positive score (CPS) > 1 receive immune checkpoint inhibitors (ICI) alone or with chemotherapy. First-line therapy of PD-L1-negative tumors consists of the TPEx regimen, combining cisplatin and docetaxel with anti-EGFR antibody Cetuximab. In second line, Cetuximab is considered as monotherapy or combined with paclitaxel [2]. Since less than 20% of patients show long-term benefits from ICI or EGFR-based therapy, novel therapeutic developments are in need to cope with challenges of R/M-HNSCC [3]. The largest umbrella trial testing genomics-guided therapies, however, has not demonstrated clinically meaningful efficacies [4]. Reasons for progression under genomics-guided therapies include enhanced intratumor heterogeneity (ITH) and a high plasticity to perturbations [5]. Both aspects rely on (epi)-genetic changes and distinct meta-programs observed at single cell level in malignant progeny. Specifically, enhanced proportions of malignant cells in partial epithelial-to-mesenchymal transition (p-EMT) were associated with nodal metastases and reduced overall survival (OS) [5, 6]. EMT enhances migration and invasion through loss of cell–cell contact promoting tumor budding (TB) and dissemination, treatment resistance, and stemness traits [7]. Comparing matched primary HNSCC and local recurrences determined a dominance of the basal molecular subtype (BA) with a frequent switch to BA in recurrences, and correlations to p-EMT and hypoxia [8].

EMT is governed by six canonical EMT transcription factors (EMT-TFs) [9] and ligand-receptor signaling with major focus on transforming growth factor β receptors (TGFβR), EGFR, NOTCH and WNT in HNSCC. EGFR-mediated EMT initiates through hyper-activation of the MAPK pathway and subsequent induction of EMT-TFs [10]. A transcriptomic signature of EGFR-mediated EMT included prognostic genes and candidate therapeutic targets [11].

We hypothesized that differential EGFR signaling and varying entailed functionalities dictate clinical responses to EGFR-based therapy. Addressing this hypothesis, a gene regulatory network of EGFR-mediated local invasion associated with EMT was identified. This network is induced by ligand-dependent EGFR-activity subtypes and comprises druggable targets for (co)-treatment with Cetuximab, and potential predictive markers.

## Methods

### Cell lines and treatments

FaDu, Kyse30, Cal27, Cal33, OSC19, HSC4 (ATCC, Manassas, VA, USA and JCRB cell bank, Neuss, Germany) were regularly confirmed via short tandem-repeat typing (STR), mycoplasma tested, and passaged in DMEM, DMEM/HAM F12 (OSC19) or RPMI, 10% FCS, 1% penicillin/streptomycin, 5% CO_2_ atmosphere at 37 °C. Treatment with EGF (50 ng/mL, Gibco Fisher Scientific, Munich, Germany), AREG (1,000 ng/mL) and EREG (80 ng/mL) (Peprotech, PeproTech, Hamburg, Germany), Activin A (Miltenyi Biotec, 130–115-012), Cetuximab (10 µg/mL, Erbitux, Merck Serono, Darmstadt, Germany), MEK1/2 (MAPK–ERK kinase) inhibitor AZD6244 (100 nM, Selleckchem, Munich, Germany), Akt signaling pathway inhibitor MK2206 (1 µM; Selleckchem, Munich, Germany), SPHK1 inhibitor PF-543 (5–10 µM, Selleckchem, Munich, Germany, s7177), Follistatin (20 ng/mL, PeproTech, Hamburg, Germany), recombinant human laminin 332 (10 μg/ml for coating and 1.6–9.0 nM for treatment and complementation of type I collagen, Biolaminin 332 LN, BioLaminaAB, Sundbyberg, Sweden), sphingosine-1-phosphate (1.5 mM, MedChemExpress, South Brunswick, US) were conducted under serum-free conditions.

### Flow cytometry

Cells were stained with ITGB4-specific antibody (439-9B, Thermo FisherScientific, Hamburg, Germany),1:100 dilution in PBS-3% FCS, 60 min on ice, washed three times in PBS-3% FCS, and stained with FITC-conjugated antibody (1:100, 40 min at 4 °C, FI-4001, Vector Laboratories/Biozol, Eching, Germany). Fluorescence intensity was assessed in a CytoFlex instrument using CytExpert Software, Version 2.2 (Beckman Coulter, Krefeld, Germany) and quantified with FlowJo software version 10.8.1 (FlowJo, Ashland, OR, USA).

### Quantitative reverse-transcription polymerase chain reaction (qRT-PCR)

After isolation of UV-irradiated cells from collagen-embedded spheroids and sorting for red Dendra2-fluorescence, total RNA was extracted with RNeasy Mini Kit (Qiagen, Germany) and transcribed into cDNA with QuantiTect Reverse Transcription kit (Qiagen, Germany). Expression of selected fDEGs was quantified by qRT-PCR in triplicates for *n* = 3 independent experiments with SYBR-Green Master PCR mix and gene-specific primers (QuantStudio3, Thermo Fisher Scientific, Germany). All mRNA quantifications were normalized to the house-keeping gene glyceraldehyde-3-phosphate dehydrogenase (GAPDH).

The following primers were used:

**Table:** 

GAPDH	FW	5´-GTCTCCTCTGACTTCAACAGCG-3´
	RV	5´-ACCACCCTGTTGCTGTAGCCAA-3´
INHBA	FW	5´-AGTCGGGGAGAACGGGTATGTGG-3´
	RV	5´-TCTTCCTGGCTGTTCCTGACTCG-3´
ITGA5	FW	5´-TGCCTCCCTCACCATCTTC-3´
	RV	5´-TGCTTCTGCCAGTCCAGC-3´
ITGB4	FW	5´-CTC CAC CGA GTC AGC CTT C-3´
	RV	5´-CGG GTA GTC CTG TGT CCT GTA-3´
LAMA3	FW	5´-GCCCAGCGCATGATGAGGGA-3´
	RV	5´-CGGTTCAGCAAGAGCTGCGACT-3´
LAMB3	FW	5´-GGCTTATCCAGGACAGGGTTG-3´
	RV	5´-GCTGCTTGGTCATGCTTGTCA-3´
LAMC2	FW	5´-CCTGCATCTGATGGACCAGCCT-3´
	RV	5´-CATGGGCCGCAGTTGGCTGT-3´
ODC1	FW	5´-GATGACTTTTGATAGTGAAGTTGAGTTGA-3´
	RV	5´-GGCACCGAATTTCACACTGA-3´−3´
RGS2	FW	5´-AAGATTGGAAGACCCGTTTGAG
	RV	5´-GCAAGACCATATTTGCTGGCT-3´
SERPINE1	FW	5´-ATCGAGGTGAACGAGAGTGG-3´
	RV	5´-ACTGTTCCTGTGGGGTTGTG-3´
SPHK1	FW	5´-GGCTTCATTGCTGATGTGGA-3´
	RV	5´-AGGAAGGTGCCCAGAGTGAA-3´

### CRISPR plasmid and gRNA

ITGB4 knockout in FaDu and Kyse30 cells was achieved using all-in-one CRISPR/Cas9 plasmids (Thermo Fisher Scientific, Munich, Germany) with ITGB4-specific guides sgRNA-1, 5’-AGAAGTTGACTCCCTCCTG-3’, and sgRNA-2, 5’-GTGCTGATGGCGCCCCGCT-3’ as described [12]. Briefly, both cell lines were transfected separately with each sgRNA-containing plasmid and selected for the co-expressed green fluorescence protein (GFP) expression. Enriched cells were selected as single cell clones in the presence of puromycin. Verification of knockouts was conducted via flow cytometry, Western blot analysis, and sequencing of affected genomic regions.

### 3D invasion

Spheroids were formed in BIOFLOAT ultra-low attachment 96-well round-bottom plates (faCellitate, Mannheim, Germany; 3000 cells/well; 72 h) and transferred into 35 mm glass bottom dishes (Sarstedt, Sarstedt, Germany) in 200 µL of serum-free medium containing type I collagen (Corning, Oak Park, Bedford, MA, USA; 1.7 mg/mL) in the absence or presence of LN5 (Laminin 332; 9 nM). Treatment was conducted with 9 nM EGF in the absence or presence of inhibitors/compounds as specified, and spheroids were allowed to invade for up to 72 h in a 5% CO2 atmosphere at 37 °C without further media changes. Images were taken using a Leica DMi8 microscope 5x/10 × in the PH channel (Leica, Nussloch, Germany). Invasive area was quantified as subtraction of core areas from total areas by ImageJ/Fuji from five to 10 spheroids per treatment. Invasive distance was quantified as average distance of the farthest cells from spheroid centers (10 cells/spheroid).

Dual core spheroids were generated with 2,000 cells/well FaDu cells stably expressing Dendra2, seeded for 24 h into 96 well BIOFLOAT ultra-low attachment plates to create a fluorescence core. After formation of the inner, fluorescent core, 8,000 FaDu-WT non-fluorescent cells were added for additional 24 h. Eventually, dual core spheroids comprise a fluorescent inner core (Dendra2-pos.) and non-fluorescent peripheral layers and enable the determination of spheroidal sub-localization of invasive cells.

### IC_50_ determination

FaDu spheroids were treated with EGF (9 nM) combined with 0—1.0 µg/mL Cetuximab with or without SPHK1 inhibitor (0.1 µM). Quantified invasive area data were evaluated by nonlinear regression analysis to determine IC_50_ values for each group (GraphPad Prism version 9.0.0 for Windows, GraphPad Software, San Diego, California USA).

### Time-lapse imaging and diffusivity test

Time-lapse microscopy was performed on a Leica DMi8 microscope equipped with an Ibidi stage top incubation system (Ibidi, Gräfelfing Germany) at 37 °C, 5% CO2, and a flow rate of 10 L/hour and 80% humidity in Ibidi 35 mm glass bottom u-dishes mounted into Ibidi incubator 35 mm inserts (#10,934, Ibidi, Martinsried, Germany). Upon completion of spheroid detection, focus (Z coordinate) was adjusted manually according to the FITC channel. All target spheroids and their positions, including X–Y-Z coordinate information, were marked and recorded in the Leica DMi8 microscope system and the LAS-X software. Microscope setting: FIM 100%, IL-Fld 6 round, time interval (20 min), exposure (PH channel 10 ms; FITC channel 50 ms), cycle (217 times = 72 h), and PH channel and FITC channel were used for 72 h observation.

### Immunohistochemistry, fluorescence imaging, immunoblotting

Normal mucosa, primary HNSCC, and nodal metastases were collected as matched triplets (*n* = 25) as reported [13] and stained with SPHK1 antibody (PA5-28,584, Invitrogen, 1:100,). Immunohistochemical quantification was performed as described [14] and compared to a semi-automated quantification using QuPath. Immunoblotting of total and phosphorylated EGFR and ERK1/2 was done according to [10] with phospho-EGFR (Tyr^1173^) (#4407), EGFR (#2232), phospho-ERK1/2 (#4370) and ERK1/2 (#4695), all from Cell Signaling Technology (Leiden, The Netherlands). Detection was performed with HRP-conjugated secondary antibody and ECL-reagent (Thermo Fisher Scientific, Munich, Germany).

### Generation of Dendra2-expressing cell clones

FaDu and Kyse30 cell lines were stably transfected with a Dendra2-Farnesyl-5 expression plasmid (#57,717, Addgene, Teddington, UK) using MATra reagent (IBA, Goettingen, Germany) and Lipofectamine™ 2000 transfection reagent (Invitrogen™, Carlsbad CA, USA), respectively. Stable clones were selected with 1150 µg/mL neomycin (G418, InvivoGen, San Diego, USA) and FACS. Dendra2 is an engineered, monomeric GFP-like photoconvertible fluorescent protein that changes its emission spectrum from green to red upon irradiation with blue and UV-light [15].

### UV-light-mediated photoconversion, and cell enrichment

Spheroids from 8,000 FaDu-Dendra2 and 10,000 Kyse30-Dendra2 cells were embedded in type I collagen and treated with serum-free medium, high-dose EGF (EGF^high^, 9 nM), EGF and Cetuximab (9 nM EGF + 10 µg/mL Cetuximab), and EGF and MEK inhibitor (9 nM EGF + 100 nM AZD6244) for 72 h. 10–12 invasive areas covering all invasive cells were selected for photoconversion by UV-light for 40 spheroids per independent experiment under stage function M&F mode (invasive cells). X–Y-Z coordinates of target areas were automatically recorded with LAS X (Leica). In addition, one single target area covering spheroid cores of EGF-treated cells (non-invasive cells), EGF plus Cetuximab (Cetuximab), or EGF plus MEK inhibitor (MEKi) treated cells were photoconverted per independent experiment. Photoconversion was conducted with a Leica DMi8 microscope system (Leica, Nussloch, Germany) in the DAPI channel. Cells in target areas were exposed with the following settings: FIM 100%, IL-Fld 2 round, time interval (2 secs), exposure (5 secs), cycle (4 times). All images were adjusted by Fiji/ImageJ and LAS X.

After photoconversion, type I collagen was degraded with 0.1 mg/mL collagenase (C9722, Sigma, Missouri, USA for 15 min, 37 °C. Recovered spheroids were centrifuged at 400 g, 4 min, collected into RNAse-free tubes (1.5 mL), and resuspended in Accutase for 10 min BD Bios (#07922, STEMCELL Technologies, Cologne, Germany). After centrifuging and washing twice with PBS, single-cell suspensions were prepared in ice-cold FACS buffer for sorting of > 1,000 cells per group into RNAse-free tubes using a FACS Aria Fusion-4 device (BD Biosciences, Heidelberg, Germany) in collaboration with the LMU Core facility.

### RNA sequencing

RNA was extracted with Qiagen RNeasy Micro Kit (Cat.74004, QIAGEN GmbH, Hilden, Germany), digested with DNAse I, quantified with a Qubit-Fluorometer, and reverse transcribed with the QuantiTect reverse transcription kit (Qiagen GmbH, Hilden, Germany). RNA quality was assessed using a Bioanalyzer 2100 System (Agilent Technologies Inc., SA) with the Agilent RNA6000Pico kit (#5067–1513, Agilent Technologies Inc., USA). Lexogen QuantSeq 3′ mRNA-Seq V2 Library Prep Kit FWD for Illumina was used according to the manufacturer´s instructions with 50 ng RNA input (SKU:015.96, Lexogen GmbH, Austria). For library preparation, optimal numbers of amplification cycles were determined using the Add-on PCR kit (SKU:020.96, Lexogen GmbH, Austria). 16 µl of library preparation were transferred to a new plate. Quant-iT™ PicoGreen™ dsDNA Assay Kit (P 7589, invitrogen, USA) and the Bioanalyzer High Sensitivity DNA Analysis Kit (#5067–4626, Agilent Technologies, Inc., USA) were used to determine the quality and concentration of libraries. An equimolar library pool was prepared, and 3′-RNA-sequencing was performed on Illumina NovaSeq platforms (Illumina, Inc. USA) by Novogene (Company Limited, UK). 3´-RNA-seq profile pre-processing was conducted by Kristian Unger. Genes with a sum of count greater than 300 across all samples were kept for further analysis.

### Differential expression (DE), functional enrichment analysis and principal component analysis

DE analysis was conducted with *DEseq2* Bioconductor package with RNA count data from cell lines and HNSCC samples. Log2FC > 1.0 and the Benjamini–Hochberg method to control the FDR at 5% were used as threshold for DEGs. Gene set enrichment analysis (GSEA) was performed with genes ranked by fold-change of DE or correlation between genes using hallmark pathways gene sets in the Molecular Signatures Database (MSigDB). Enrichment analysis was conducted and visualized with *clusterProfiler* package and *enrichplot* package (Bioconductor). Principal component analysis (PCA) of bulk 3´-RNA-seq expression data was performed with *prcomp* R function.

### Pathway activation inference

Based on tumor mRNA expression data, signaling activity of 14 pathways was inferred by Pathway response GEnes for activity inference (*PROGENy*). Spearman correlation and significance between genes and pathway activation were visualized with *corrplot* R function.

### External datasets

**GSE103322**: scRNA-seq analysis of *n* = 10 patients with OSCC (*n* = 5,902 total cells) using SmartSeq2 [5].

**GSE181919**: scRNA-seq analysis of *n* = 23 HNSCC-patients with *n* = 37 tissue specimens of non-tumoral normal tissue (NL, *n* = 9), leukoplakia (LP, *n* = 4), primary cancer (CA, *n* = 20), and metastatic tumors in lymph nodes (LN, *n* = 4) (*n* = 54,239 total cells) [16].

**GSE188737**: scRNA-seq analysis of *n* = 24 HNSCC patients including matched pairs of primary tumors (*n* = 7) and nodal metastasis (*n* = 7) (*n* = 53,459 total cells) [17].

**GSE208253**: Spatial transcriptomic dataset of *n* = 12 oral squamous cell carcinoma (OSCC). GSE208253 was generated utilizing the Visium platform from 10 × Genomics at a spatial resolution of 55 μm per spot [18].

**GSE65021**: Whole genome-cDNA-mediated annealing, selection, extension, and ligation (WG-DASL) microarray analysis of *n* = 40 R/M-HNSCC patients treated with Cetuximab in combination with platinum-based chemotherapy [19].

**GSE65858**: Gene expression analysis of *n* = 270 HPV-neg. and -pos. HNSCC using Illumina array [20]. *n* = 196 HPV-neg. HNSCC patients were used in the present study.

**GSE84713**: Affymetrix gene expression (HG U133 Plus 2.0 array) profiles of *n* = 28 HNSCC patient-derived xenotransplants in dependency to treatment response to Cetuximab [21].

**TCGA-HNSCC**: Bulk-seq analysis of HPV-neg. HNSCC (*n* = 415) within The Cancer Genome Atlas (TCGA) [22], including detailed and systematic patient data (Suppl. Table 2). Dataset was downloaded from cBioportal at: https://cbioportal-datahub.s3.amazonaws.com/hnsc_tcga_pan_can_atlas_2018.tar.gz.

### GSEA, scRNA-seq and spatial transcriptomic analysis

GSEA analysis was carried out with *DEseq2*-determined DEGs ranked according to log fold changes between groups as input for the GSEA function in 'clusterProfiler' (fGSEA). Spatial transcriptomic dataset GSE208253 was collected from the Gene Expression Omnibus (GEO). Preprocessing steps encompassed quality control measures to exclude spots that exhibited fewer than 500 detected genes or had mitochondrial gene expression levels above 20%. Data normalization was performed using the SCTransform function of “Seurat” package. In addition, we integrated the cluster annotation (normal region, leading edge, transitory region, and tumor core) originally provided in GSE208253 for subsequent analyses [18]. Based on spatial spots with malignant tumor, unsupervised Louvain clustering was performed to divide these regions into three major clusters. Differential expression gene analysis was conducted to annotate these clusters. Marker gene expression patterns were used to define Cluster 1 as "Tumor Core" (TC; CLDN4 as marker), Cluster 3 as "Leading Edge" (LE; LAMC2 as marker), and Cluster 2, exhibiting features of both, as "Transitory region." Based on the above cluster annotation, we extracted and integrated spots corresponding to the LE and TC regions from each sample. To detect expression differences of genes of interest between LE and TC, differential analysis was conducted using a Student’s t-test to assess the statistical significance (*p < 0.0332, ****p < 0.0001).

### Non-negative matrix factorization (NMF) clustering, CellChat

After preliminary sample integration of GSE181919 via “Harmony” (https://github.com/immunogenomics/harmony) and “Seurat” (V4; https://satijalab.org/seurat/articles/get_started.html), eight common cell types were labeled based on common cell markers, and epithelial cells extracted [16]. Genes sets were downloaded from MSigDB (https://www.gsea-msigdb.org/gsea/msigdb) and Reactome (https://reactome.org/). The according gene signatures characterized well-defined biological activities, states, and processes (MSIGDB-hallmarks), including the EGFR activity signature genes “REACTOME_SIGNALING_BY_EGFR” (https://www.gsea-msigdb.org/gsea/msigdb/human/geneset/REACTOME_SIGNALING_BY_EGFR.html), respectively. *AddModuleScore* served to calculate target gene sets in epithelial cells and correlations were visualized using *corrplot*. Subtypes of EGFR activity were identified through non-negative matrix factorization-based (R package “*NMF”*) dimension reduction analysis using “REACTOME_SIGNALING_BY_EGFR” (*n* = 48 of 50 genes showed sufficient expression level) across all epithelial cells. Seurat object *FindAllMarkers* and *Cytotrace/2* (https://cytotrace.stanford.edu/ and https://github.com/digitalcytometry/cytotrace2) were used to analyze gene expression differences between cell clusters, which were visualized via volcano plot and gene distribution map, respectively (function *ggplot* and *DimPlot*). Interactions between epithelial cells and non-malignant cells were addressed with the CellChat package (https://github.com/sqjin/CellChat) using pre-set thresholds.

To assess correlations with clinical endpoints, top 100 genes correlated with CytoTRACE scores were extracted (top *n* = 50 positively and *n* = 50 negatively correlated genes) and their association with overall survival (OS) was evaluated by univariate Cox regression analysis in HPV-neg. patients of GSE65858 (*n* = 196) [20]. Nineteen genes were significantly associated with OS and were included in a multivariate Cox regression-based prediction model for OS. The established predictive model was validated in HPV-neg. patients of the TCGA-HNSC cohort (*n* = 415; see “External datasets”). Stratification of patients based on risk score at median split was visualized in Kaplan–Meier survival curves.

### Weighted gene co-expression network analysis (WGCNA)

Weighted gene co-expression network analysis (WGCNA) was employed to identify co-expressed gene modules using “*WGCNA*” R package. Top 25% most variant genes across samples, based on overall sums of counts across all samples, were implemented into WGCNA. A soft threshold power (β) of five was chosen to construct a scale-free network, achieving a scale-free topology model fit (R^2^ = 0.90). WGCNA function “*blockwiseModules*” was used to construct signed hybrid, weighted correlation networks. Module-trait correlations were calculated by coding ssGSEA-derived pathway scores (EMT, MAPK, PI3K, EGF_EGFR) as a binary matrix. Resulting module-trait correlations were visualized using WGCNA built-in function ‘*Heatmap’*, displaying Spearman correlation and significance (*p*-value) of traits versus modules.

### Regulatory network analysis

Input to the regulatory network analyses were counts per million (CPM) expression matrices obtained from 3´-RNA-seq of untreated and invasive cells from FaDu and Kyse30. Regulatory network analysis was based on the ARACNe-inferred network of the TCGA-HNSCC cohort, retrieved via the *aracne.networks* R package (version 1.24.0) comprising 6,055 regulators, 19,722 targets and 423,104 interactions (https://bioconductor.org/packages/release/data/experiment/html/aracne.networks.html). Results from differential expression analysis (Wilcoxon rank-sum test) were visualized as a gene regulatory network using *ggraph* R package (version 2.1.0) [23] in “*stress*” layout.

### Tumor budding analysis

Tumor budding (TB) was evaluated according to [24]. Hematoxylin/eosin-stained digital slides of HPV-negative HNSCC (*n* = 286) from the TCGA-HNSCC cohort were assessed for tumor budding, defined as clusters of up to four tumor cells dissociating from the tumor mass and infiltrating into the surrounding stroma [25]. Evaluation was performed by trained pathologists, documenting absolute numbers of tumor buds in ten consecutive high-power fields (HPFs) with one HPF covering an area of 97,464 μm^2^ in the digitized HE-stained slide starting with the HPF including the highest number of buds [24]. A two-tiered classification scheme was applied comparing budding (≥ 1 bud in 10 HPF) with non-budding tumors. Functional DEGs (fDEGs) were analyzed for differential expression between budding and non-budding tumors using DEseq2 and the Benjamini–Hochberg method to control false-discovery rates (FDR) at 5%.

### Statistical analysis

Expression differences between sample groups were compared by Wilcoxon test. Correlation between gene expression was calculated by Spearman’s correlation. Multivariate logistic regression model was applied for testing odd ratios of gene expression level with relapse period since anti-EGFR treatment. Comparison of two or more groups was performed using a student t-test and one or two-way ANOVA with a Tuckey´s post-hoc multiple pairwise comparison test, respectively. Data analysis was performed in R statistical platform (version 4.1.2 (2021–11-01)). All significant *p*-values were marked as follows in figures: * *p*-value < 0.05, ** < 0.01, *** < 0.001, and **** < 0.0001. Unmarked pairs do represent *p*-values > 0.05. Numerical *p*-values for all statistical analyses are compiled in Suppl. Table “*p*-values_Figures”.

## Results

### Function-based enrichment of locally invasive malignant cells

**Fig. 1:**
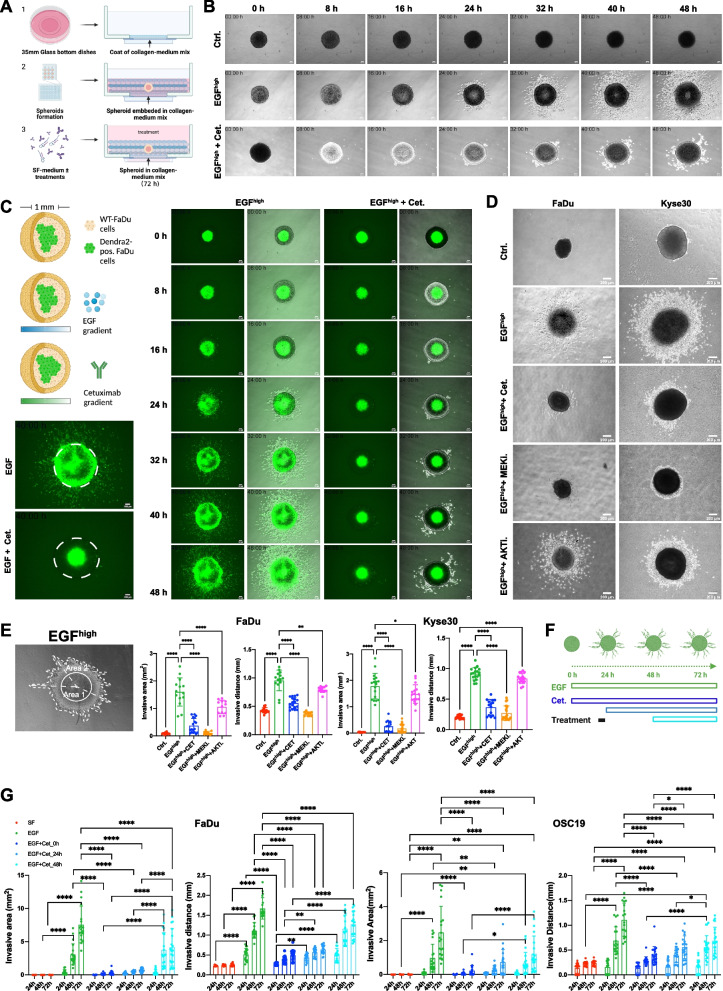
EGFR-mediated local invasion in a 3D spheroid model. **A** Schematic representation of the 3D invasion model showing type I collagen-coated culture dishes (1), spheroid formation in 96-well format (2), and treatment of spheroid in ECM (3). **B** FaDu cell spheroids were treated with the indicated compounds (Ctrl.: control treatment with serum-free medium, EGF^high^, EGF^high^ plus Cetuximab (10 µg/mL). Shown are representative micrographs of a time lapse analysis at the indicated time points from at least three independent experiments performed with > five spheroids each. **C** Upper left: Schematic representation of a dual core spheroid containing a central area composed of Dendra2-positive FaDu engulfed in parental, non-fluorescent FaDu cells. Representative immunofluorescence micrographs with EGF or EGF plus Cetuximab are shown. **D** Spheroids of FaDu and Kyse30 cells were treated with serum-free medium (Ctrl.), EGF^high^, and EGF^high^ in combination with Cetuximab (Cet.), MEK inhibitor (MEKi.), or AKT inhibitor (AKTi.). Shown are representative micrographs from *n* = 3 independent experiments performed with 7–8 spheroids per experiment. **E** Left: Quantification areas for local invasion are exemplified in FaDu cells after EGF^high^ treatment. Right: Shown are mean with SD of invasion area and invasive distance from n = 3 independent experiments with 7–8 spheroids each, following treatment of FaDu and Kyse30 with indicated compounds (Ctrl.: serum-free medium; EGF^high^; CET: Cetuximab; MEKi.: MEK inhibitor; AKTi.: AKT inhibitor). **F** Scheme of concurrent and time-delayed Cetuximab treatment in EGFR-mediated local invasion. Cells were treated with a single dose of EGF at 0 h and remained either untreated or were further treated with Cetuximab as a single dose at 0, 24, or 48 h. Shown are treatment time points and duration (open rectangles) (**G**) Shown are mean with SD of invasion area and invasive distance from *n* = 3 independent experiments with 7–8 spheroids each, following treatment of FaDu and OSC19 spheroids with EGF^high^ and Cetuximab at the indicated time points. Local invasion of untreated cells (SF, red), EGF- (green), EGF plus Cetuximab concurrently (dark blue), EGF plus Cetuximab after 24 h (light blue) or 48 h (cyan). Asterisks reflect significances of Tuckey´s multiple post-hoc comparisons following a 2-way ANOVA. *p*-value < 0.05, ** < 0.01, *** < 0.001, and **** < 0.0001. Unmarked pairs do all represent *p*-values > 0.05

The effects of high-dose EGF (EGF^high^, 9 nM) and selected inhibitors on local invasion were addressed in FaDu and Kyse30 cell spheroids embedded in type I collagen (Fig. 1A). FaDu (hypopharyngeal) cells harbor a wildtype *EGFR* gene, with neither mutations nor copy number variations (CNV + 0). Kyse30 cells (esophageal) have an amplification of *EGFR* (CNV + 1) without further mutations in *EGFR* [11, 26, 27]. Both cell lines have been chosen based on their responsiveness towards EGFR-mediated EMT and reported gene expression profiles [11]. Untreated FaDu spheroids showed no signs of spontaneous invasion in time-lapse imaging, whereas high concentrations of EGF reported to induce EMT in 2D cultures [11] promoted detachment and invasion of tumor cells into type I collagen, which was blocked by concomitant Cetuximab treatment (Fig. 1B and Suppl. Video 1).

Penetration depths of EGF and Cetuximab and initial sub-localizations of invasive cells were addressed in dual core spheroids composed of central fluorescent FaDu cells (core) and peripheral, non-fluorescent wild-type FaDu cells. Fluorescence time-lapse imaging of dual core spheroids after EGF^high^ treatment revealed invasive peripheral and core cells (Fig. 1C and Suppl. Video 2), which were equally well inhibited by Cetuximab (Fig. 1C and Suppl. Video 3). Inhibition with Cetuximab and MEK inhibitor (MEKi; AZD6244) but not AKT inhibitor (AKTi, MK2206) significantly reduced EGF ^high^-induced local invasion (Fig. 1D, E). Thus, EGF and Cetuximab entirely penetrate spheroids (diameter approx. 1 mm) to deploy their MEK-dependent inducing and inhibiting activities, respectively.

It is so far unclear if an initial EGFR trigger is sufficient or whether local invasion requires sustained EGFR activation and, accordingly, effects on Cetuximab efficacy after initiation of local invasion remain unknown. FaDu and tongue cell line OSC19 were treated with a single dose of EGF for 72 h (EGF treatment at 0 h) in combination with one dose of Cetuximab either concurrently (Cetuximab treatment at 0 h) or after 24 h or 48 h of ongoing EGF treatment without further media changes. EGF treatment of both cell lines for 72 h without addition of Cetuximab served as positive controls for maximal invasion (Fig. 1F). Significant local invasion of FaDu and OSC19 cells started at 48 h and further increased at 72 h. Concomitant (0 h) and early (24 h) administration of Cetuximab entirely blocked invasion, while administration after 48 h inhibited any further local invasion. Thus, the results support a requirement for sustained activation of EGFR during local invasion (Fig. 1G).

**Fig. 2:**
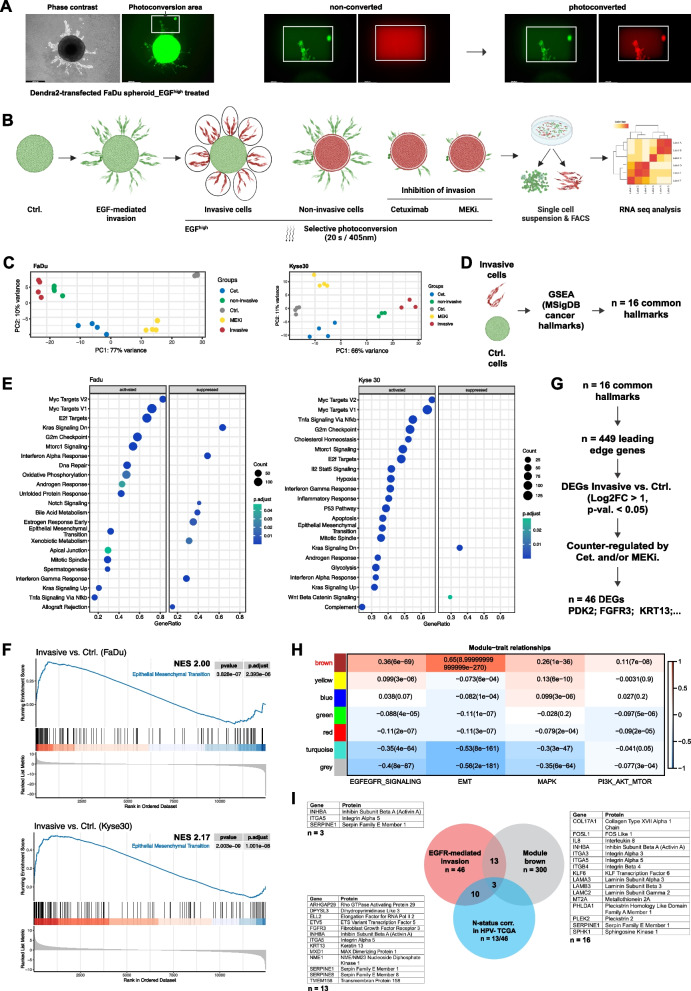
Identification of fDEGs in locally invasive cells. **A** Photoconversion of Dendra2-positive, invasive FaDu cell spheroids showing selected cells before and after conversion. **B** Selection process of invasive cells upon photoconversion, flow sorting, and RNA-seq of the indicated samples. **C** Principal component analysis of control (Ctrl), invasive, non-invasive, Cetuximab (Cet.) or MEK inhibitor (MEKi) co-treated cell populations from FaDu and Kyse30 cell spheroids. **D** Scheme of the gene set enrichment analysis (GSEA) of invasive versus control cells using human cancer hallmarks (MSigDB) resulting in the identification of *n* = 16 commonly regulated hallmarks of FaDu and Kyse30 cells. **E** Significantly activated and suppressed hallmarks in invasive cells of FaDu (*n* = 23) and Kyse30 cells (*n* = 22). **F** Enrichment score curves of the EMT hallmark of invasive versus control-treated FaDu and Kyse30 cells including normalized enrichment scores (NES), *p*-values and adjusted *p*-values, and ranked list metrics. **G** Leading edge genes were extracted from *n* = 16 common hallmarks and *n* = 46 genes with a Log2 fold change (Log2FC) > 1, significant *p*-value (< 0.05) and counter-regulation after Cetuximab and/or MEKi were identified and considered functional DEGs (fDEGs). **H** WGCNA of malignant HNSCC cells (*n* = 2,176; GSE103322) showing modules of co-regulated genes and correlations to EGFR, MAPK, and PI3K/AKT activity, and EMT (Spearman correlations and *p*-values). **I** Venn diagram of intersected genes between fDEGs, gene module brown, and genes correlated with the N-status of HPV-negative HNSCC-TCGA samples. Specific genes are listed in the according tables

FaDu-Dendra2 and Kyse30-Dendra2 spheroids were treated with serum-free medium, high-dose EGF, EGF and Cetuximab, and EGF and MEK inhibitor for 72 h in the described 3D model of local invasion. Areas of invasive cells commonly representing 10–12 circular areas per spheroid were subjected to photoconversion by UV-light in 40 spheroids per treatment. Separately, one area covering spheroid cores of EGF-treated cells (non-invasive cells), EGF plus Cetuximab (Cetuximab), and EGF plus MEK inhibitor (MEKi) treated cells was photoconverted. The latter samples were acquired to address transcriptomic effects in cells lacking signs of invasive potential despite EGF-treatment and following inhibition of invasion by Cetuximab and MEK inhibitor. After collagen degradation, single-cell suspensions were sorted according to red Dendra2-fluorescence (> 1,000 cells per group) and were analyzed by 3´-RNA-seq (Fig. 2A, B). Final samples included controls (Ctrl), invasive (typ I collagen-resident after 72 h) and non-invasive cells (spheroid-resident after 72 h), EGF^high^/Cetuximab and EGF^high^/MEKi treatments (Fig. 2B). Principal component analysis (PCA) determined highest transcriptional distances between control cells and invasive and non-invasive cells (PC1). Cetuximab or MEKi co-treated samples clustered away from control, invasive, and non-invasive cells in PC1, and differed in PC2 (Fig. 2C). These findings suggested strongest differences between EGF-treated and untreated cells, and a reversion of transcriptomic changes upon blockade with Cetuximab and MEK inhibitor.

To interrogate transcriptional changes induced by EGF treatment that entail local invasion, a gene set enrichment analysis (GSEA) of invasive cells versus untreated controls was conducted, identifying 16 of the 50 hallmark pathways of the Molecular Signature Database (MSigDB) that were common to FaDu and Kyse30 cells (Fig. 2D, E and Suppl. Table 1). EMT was activated with normalized enrichment scores (NES) of 2.0 and 2.17 in FaDu and Kyse30 cells, respectively, experimentally linking EMT to local invasion (Fig. 2F). Leading-edge genes extracted from 16 common hallmark pathways with a Log2FC > 1.0 and FDR < 5% were further analyzed regarding their response to Cetuximab and MEK inhibitor by comparison with the according samples. Eventually, only genes that were counter-regulated in their expression by Cetuximab and/or MEKi were considered differentially expressed genes that followed functional effects of EGF, Cetuximab, and MEK inhibitor on invasion (functional differentially expressed genes, fDEGs, *n* = 46; Fig. 2G and Suppl. Table 1). The relevance of the identified fDEGs to patients was addressed in HNSCC tumor cells on the single cell level. For this, weighted gene co-expression network analysis (WGCNA) of malignant HNSCC cells within the external scRNA-seq dataset GSE103322 (*n* = 2,176 [5]) served to identify module “brown” as most strongly associated with EGF-EGFR/MAPK signaling activity and EMT. Upon intersection with *n* = 46 fDEGs, *n* = 16 common genes were identified (Fig. 2I). Furthermore, thirteen of *n* = 46 fDEGs were significantly associated with nodal metastases in the publicly available HPV-negative HNSCC cohort [22] (Supplementary Table 2). Finally, *INHBA*, *ITGA5*, and *SERPINE1* represent fDEGs associated with EGFR/MAPK and EMT in single malignant cells that correlate with the presence of nodal metastases in the large external TCGA-HNSCC cohort (Fig. 2I).

Expression and pathway associations of fDEGs were assessed in malignant HNSCC cells (GSE103322) (Suppl. Figure 1A). *KLF6*, *ITGB4*, *LAMA3*, *LAMB3*, *LAMC2*, *MT2A*, *PHLDA1*, and *SERPINE1* displayed the most consistent expression and distribution (Suppl. Figure 1B, C), and *INHBA*, *ITGA5*, and *SERPINE1* were significantly enhanced in TCGA-HNSCC patients with nodal metastases (Suppl. Figure 1D). PROGENy pathways revealed strongest fDEGs correlations with EGFR, tumor necrosis factor (TNF), transforming growth factor beta (TGFβ), and nuclear factor kappa B (NFκB) pathway activity (Suppl. Figure 1E). Increasing expression level and numbers of cells expressing fDEGs were observed throughout tumor progression from normal epithelia to leukoplakia and carcinoma cells in the external dataset GSE181919 [16] (Suppl. Figure 2A-D). Expression of fDEGs was frequently decreased from primary carcinoma to nodal metastases except for SPHK1 in matched pairs of primary carcinoma and cervical lymph node metastases of treatment-naïve, HPV-negative HNSCC in external dataset GSE188737 [17] (Suppl. Figure 2E). Correlations of all fDEGs were observed with EGFR, MAPK, Androgen, TNF, and TGFβ (Suppl. Figure 2F, G).

To exclude major effects of UV-light irradiation on gene transcription in the presented model, FaDu-Dendra2 and Kyse30-Dendra2 spheroids were UV-irradiated under conditions identical to cell selection via photoconversion. Following isolation and enrichment of photoconverted cells by FACS, expression of selected fDEGs (INHBA, ITGA5, LAMA3, LAMB3, LAMC2, SERPINE1, and SPHK1) was assessed via quantitative RT-PCR and was compared with non-irradiated cells. Quantification of mRNA expression ratios (UV/Ctrl.) demonstrated no or negligeable effects of UV-light irradiation on gene expression under the applied conditions of short-term exposure (Suppl. Figure 3).

### An actionable gene regulatory network of EGFR-mediated local invasion

**Fig. 3:**
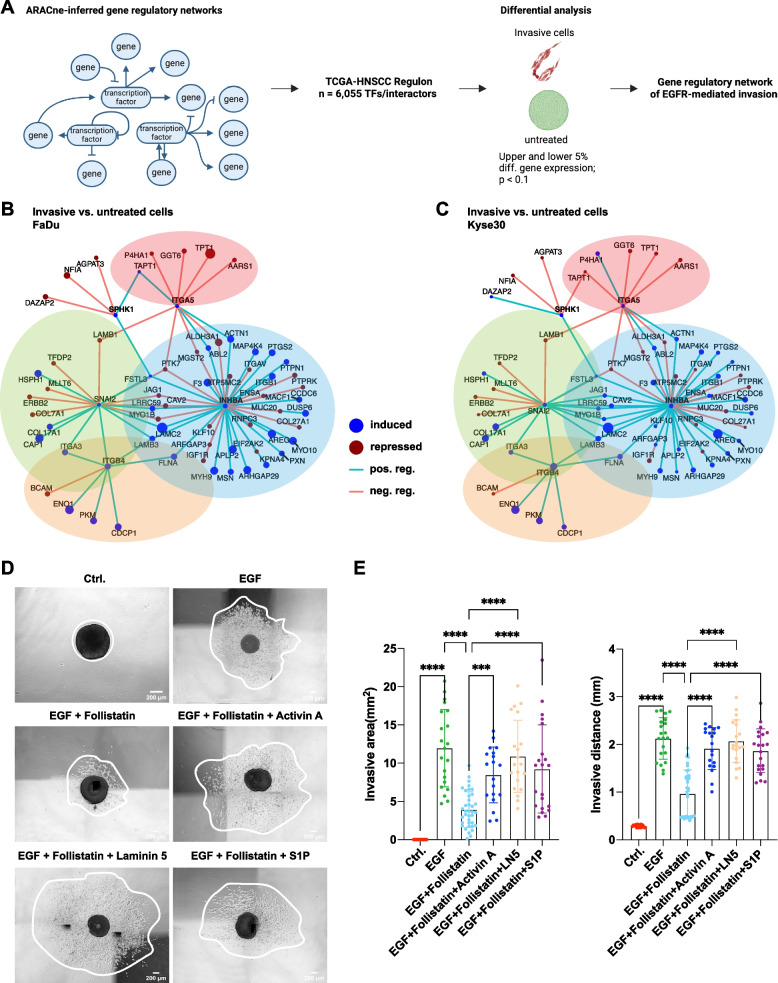
Gene regulatory network of invasive HNSCC cells. **A** Schematic depiction of the gene regulatory network analysis of invasive cells (invGRN). ARACne-inferred GRN from HPV-neg. TCGA-HNSCC (6,055 transcription factors and interactors) served to establish an EGFR-mediated invGRN of FaDu and Kyse30 invasive vs. untreated cells (upper and lower 5% DEGs and *p* > 0.1). **B**, **C** InvGRN are depicted for FaDu (**B**) and Kyse30 cells (**C**). Symbol sizes define levels of differential expression. Blue = induced, red = repressed. Connecting lines define positive (blue) or negative (red) regulation. **D**, **E** FaDu cell spheroids embedded in type I collagen were kept untreated (Ctrl, serum-free), treated with high-dose EGF alone or in combination with Follistatin (Activin A inhibitor), and additionally with activin A, laminin 5 (LN5), or sphingosine-1-phsophate (S1P). Shown are representative micrographs in (**D**) and mean with SD of invasive area (left) and distance (right) from *n* = 3 independent experiments with 5–10 spheroids per treatment and experiment in (**E**). *** *p*-value < 0.001; **** *p*-value < 0.0001

The published ARACNe-defined TCGA-HNSCC transcriptional regulon [28] including 6,055 regulators served to infer a gene regulatory network of invasive cells (invGRN) from our own gene expression data. Comparative analysis was conducted with differential gene expression of invasive and control-treated cells using the 5% most strongly up-regulated and the 5% most down-regulated genes for FaDu and Kyse30 cells (Fig. 3A). An invGRN identified *INHBA*, *SNAI2*, *ITGB4*, and *ITGA5* as common induced regulatory hubs of FaDu and Kyse30 invasive cells (Fig. 3B, C). Competitive inhibition of the homodimerized gene product of *INHBA* (Activin A) with Follistatin reduced EGFR-mediated local invasion of FaDu cells, which was rescued by co-treatment with recombinant Activin A (Fig. 3D, E). *LAMB3* and *LAMC2* are two of three genes composing the ITGB4 ligand laminin 5 (LN5) that are induced by *INHBA*. Complementation with recombinant LN5 reversed Follistatin effects, as did the enzymatic product of SPHK1, sphingosine-1-phosphate (S1P) (Fig. 3D, E). Hence, the invGRN is interactively operational in regulating EGFR-mediated local invasion.

### LN5 triggers EGFR signaling

**Fig. 4:**
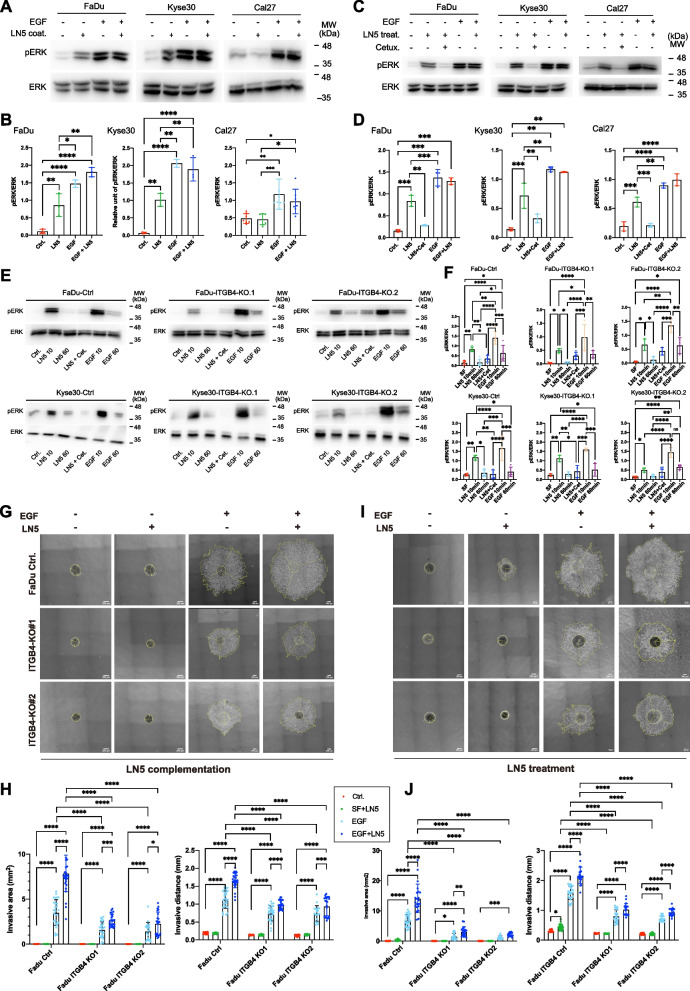
LN5 activates EGFR and fosters local invasion. **A**, **B** FaDu, Kyse30, and Cal27 cells were treated with EGF (10 min) or cultured on LN5 -coating (42 h) under serum-free conditions. Phosphorylated ERK (pERK) and total ERK1/2 were detected by immunoblotting with specific primary antibodies. Shown are representative immunoblots from *n* = 3 independent experiments in (**A**). Quantification of *n* = 3 independent immunoblots as mean with SD and statistical significance is shown in (**B**). * *p*-value < 0.05, ** 0.01, *** 0.001, **** 0.0001. **C**, **D** FaDu, Kyse30, and Cal27 cells were treated with EGF, LN5, LN5 with Cetuximab (Cetux.), or EGF and LN5 (10 min). Phosphorylated ERK (pERK) and total ERK1/2 were detected by immunoblotting with specific primary antibodies. Shown are representative immunoblots from *n* = 3 independent experiments in (**C**). Quantification of *n* = 3 independent immunoblots as mean with SD and statistical significance is shown in (**D**). * *p*-value < 0.05, ** 0.01, *** 0.001, **** 0.0001. **E**, **F** FaDu and Kyse30 WT, ITGB4-Ctrl, ITGB4-KO1 and KO2 cells were treated with LN5 (10 and 60 min), LN5 and Cetuximab (LN5 + Cet., 10 min), and EGF (10 and 60 min). Phosphorylated ERK (pERK) and total ERK1/2 were detected by immunoblotting with specific primary antibodies. Shown are representative immunoblots from *n* = 3 independent experiments in (**E**) for each cell line. Quantification of n = 3 independent immunoblots is shown in (**F**) as mean with SD and statistical significance. * *p*-value < 0.05, ** 0.01, *** 0.001, **** 0.0001. **G**, **H** FaDu ITGB4-Ctrl, ITGB4-KO1 and KO2 cells were grown as spheroids and embedded in type I collagen with or without LN5 in combination with EGF as indicated. Shown are representative spheroids with invasive area and distance from *n* = 3 independent experiments with 5–10 spheroids each per treatment in (**G**). Quantification of local invasion from *n* = 3 independent experiments with 5–10 spheroids each per treatment as mean invasive areas and distances with SD is shown in (**H**). * *p*-value < 0.05, *** < 0.001, **** < 0.0001. **I**, **J** FaDu ITGB4-Ctrl, ITGB4-KO1 and KO2 cells were grown as spheroids, embedded in type I collagen, and treated with LN5, EGF, or LN5 and EGF. Shown are representative spheroids from *n* = 3 independent experiments with 5–10 spheroids each per treatment in (**I**). Quantification of local invasion from *n* = 3 independent experiments with 5–10 spheroids each per treatment as mean invasive areas and distances with SD is shown in (**J**). * *p*-value < 0.05, ** < 0.01, *** < 0.001, **** < 0.0001

Long-term treatment with immobilized LN5 significantly induced ERK1/2 phosphorylation in FaDu and Kyse30, but not in tongue squamous cells carcinoma line Cal27 (Fig. 4A, B). Short-term treatment (10 min) with soluble LN5 significantly induced pERK1/2 in all cell lines and was sensitive to Cetuximab (Fig. 4C, D). Activation occurred as early as 5 min, peaked at 30 min, and was more transient in Cal27 cells, potentially explaining the lack of long-term induction (Suppl. Figure 4A, B). Supplementation of LN5 to extracellular matrix fostered EGF-induced local invasion in a concentration-dependent manner (Suppl. Figure 4C, D).

FaDu and Kyse30 control and *ITGB4* knockouts (Suppl. Figure 5) served to interrogate a potential ITGB4-dependency of LN5-mediated effects. Treatment of control and ITGB4 knockouts with soluble LN5 induced a transient and Cetuximab-sensitive activation of ERK1/2 after 10 min, which was back to base-level at 60 min in FaDu and Kyse30. Activation of ERK1/2 by EGF was unaffected by ITGB4 knockout and showed comparable dynamics to control lines (Fig. 4E). Comparison of pERK/ERK1/2 ratios following LN5-treatment between control and ITGB4 knockout clones showed no or a mild reduction in the absence of ITGB4 and was accompanied by reduced steady-state pER1/2 in absence of growth factors (Fig. 4F). Control spheroids behaved identically to wild-type FaDu with an approx. two-fold enhanced EGF-induced local invasion upon complementation of type I collagen with LN5. ITGB4 knockout significantly reduced local invasion but did not abrogate LN5 effects on local invasion (Fig. 4G, H). Treatment with soluble LN5 alone induced a minor increase in invasive distance and substantially enhanced EGF-mediated local invasion. In ITGB4 knockouts, soluble LN5 retained its ability to foster local invasion, although with reduced amplitude compared to controls (Fig. 4I, J). Thus, LN5 acts as a structural anchor for ITGB4 in ECM and as a novel ITGB4-independent activator of EGFR signaling and invasion.

### Sphingosine-1-phosphate contributes to local invasion

**Fig. 5:**
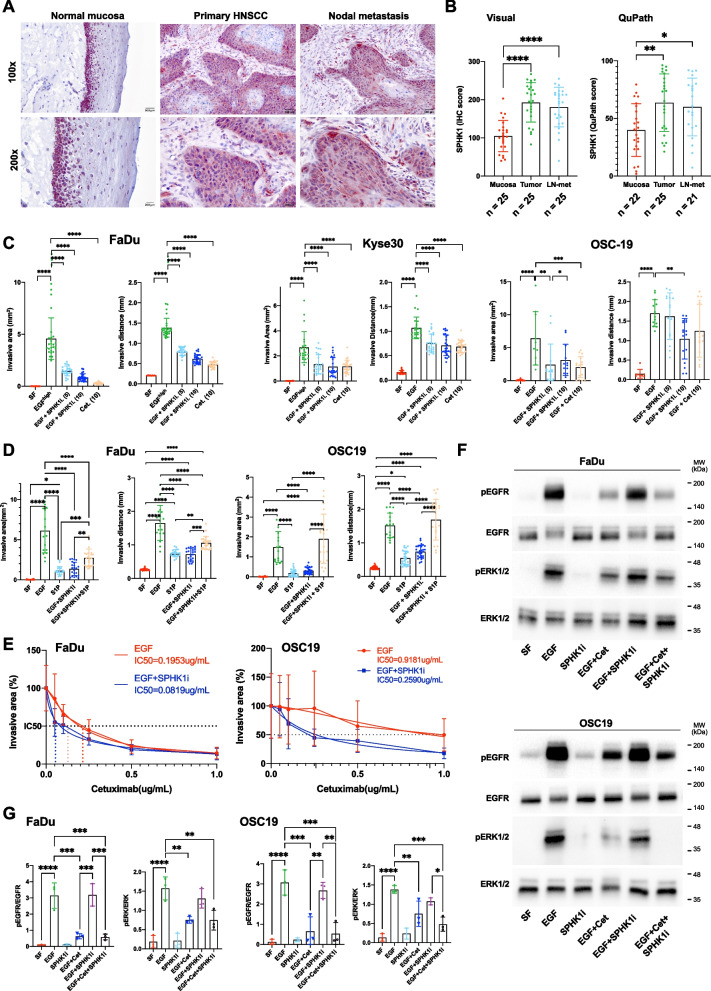
SPHK1 contributes to EGFR-mediated local invasion. **A** SPHK1 was detected by immunohistochemistry in sections of matched triplets of normal mucosa, primary HNSCC, and nodal metastasis (*n* = 25). One representative matched triplet is shown. **B** SPHK1 expression was quantified visually (left) or using the QuPath software following scanning of samples (right). **C** FaDu cell spheroids were embedded in type I collagen and treated with EGF, and EGF in combination with SPHK1 inhibitor (5 and 10 µM) or Cetuximab (Cet., 10 µg/mL). Quantification of local invasion from *n* = 3 independent experiments with 5–10 spheroids each per treatment is shown as mean invasive areas and distances with SD for FaDu, Kyse30, and OSC-19 cells. * *p*-value < 0.05, ** < 0.01, *** < 0.001, **** < 0.0001. **D** FaDu and OSC-19 spheroids were treated with EGF, S1P, and SPHKi, as indicated. Quantification of local invasion from *n* = 3 independent experiments with 5–10 spheroids each per treatment is shown as mean invasive areas and distances with SD for FaDu and OSC-19 cells. * *p*-value < 0.05, ** < 0.01, *** < 0.001, **** < 0.0001. **E** FaDu and OSC19 cell spheroids were embedded in type I collagen and treated with EGF to induce local invasion. Co-treatment was conducted with increasing concentration of Cetuximab in the presence or absence of a steady concentration of SPHK1 inhibitor to determine the functional IC50. Shown are mean with SD from *n* = 3 independent experiments. **F** FaDu and OSC19 cells were treated with EGF, SPHK inhibitor, Cetuximab as indicated for 10 min under serum-free conditions. Levels of phosphorylated EGFR, total EGFR, phosphorylated ERK1/2, and total ERK1/2 were assessed by immunoblotting with specific antibodies. Shown are representative immunoblots from *n* = 3 independent experiments. **G** Quantification of *n* = 3 independent immunoblots as mean with SD and statistical significance. * *p*-value < 0.05, ** 0.01, *** 0.001, **** 0.0001

Sphingosine kinase 1 (SPHK1) is a druggable kinase within the invGRN that was quantified in an in-house cohort of matched normal mucosae, primary tumors, and lymph node metastases (*n* = 25). SPHK1 was expressed in basal/suprabasal layers in normal epithelium and was upregulated in malignant cells of primary tumors and nodal metastases (Fig. 5A). Manual and semi-automated QuPath-based quantification of SPHK1 protein expression (immunohistochemistry) confirmed significantly higher expression of SPHK1 in primary tumors and nodal metastases compared to dysplasia-free mucosae (Fig. 5B).

Invasion induced by EGF^high^ in FaDu, Kyse30, and tongue carcinoma line OSC19 was significantly repressed by SPHK inhibition (Fig. 5C and Suppl. Figure 6A). Treatment with sphingosine-1-phosphate (S1P) alone induced mild local invasion and partly or entirely reverted effects of SPHK1-inhibitor in FaDu and OSC19, respectively (Fig. 5D and Suppl. Figure 6B). Co-treatment with a SPHK1-inhibitor concentration inefficient as single active substance (0.1 µM) reduced the IC_50_ of Cetuximab for half-maximal inhibition of local invasion in FaDu (60% IC_50_ reduction) and OSC19 (71.8% IC_50_ reduction) (Fig. 5E and Suppl. Figure 6C, D). Next, FaDu and OSC19 cells were induced with EGF in combination with Cetuximab, SPHK1-inhibitor, or both drugs. Activating phosphorylation EGFR and of the central nod ERK1/2, which is mandatory for target gene, EMT, and invasion induction [11, 13], were assessed by immunoblotting. SPHK1-inhibitor did neither affect EGFR nor ERK1/2 phosphorylation and did not enhance Cetuximab-mediated inhibition of EGFR and ERK in FaDu and OSC19 cells (Fig. 5F, G). Hence, SPHK1 inhibition suppresses EGF-mediated local invasion and shows synthetic effects with Cetuximab independently of an inhibition of upstream EGFR signaling.

### EGFR-activity subtypes regulating fDEGs and EMT

**Fig. 6:**
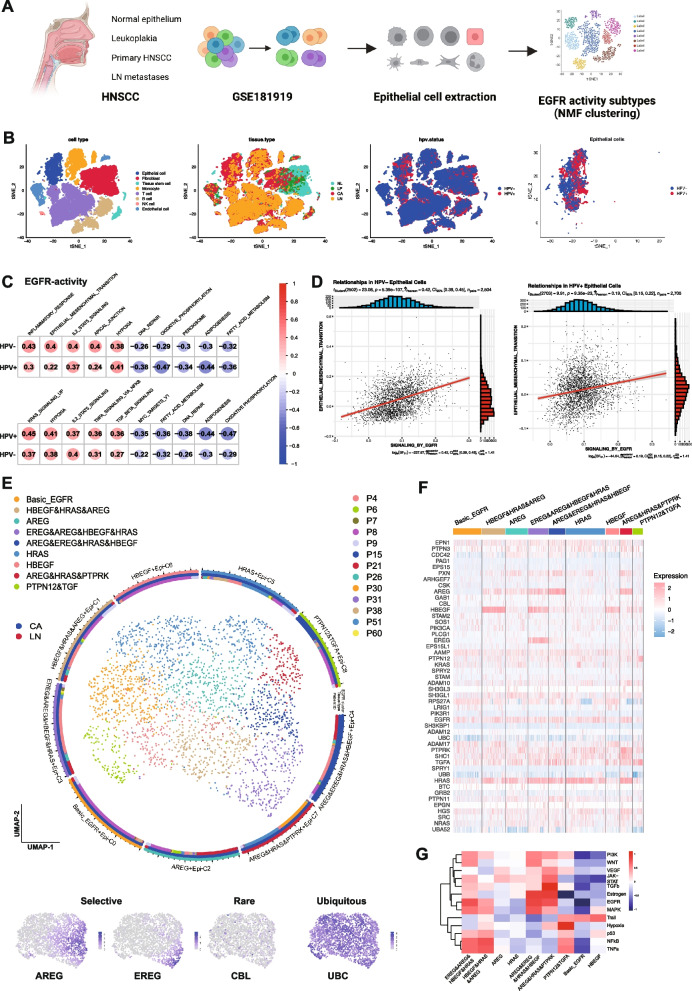
Identification of EGFR-activity subtypes in HNSCC. **A** scRNA-seq dataset GSE181919 served to extract normal epithelium, leukoplakia, primary HNSCC, and nodal metastases-derived epithelial cells. EGFR-activity subtypes were identified by non-negative matrix factorization using the “REACTOME_SIGNALING_BY_EGFR” signature. **B** UMAP of cell types, tissue of origin, HPV-status, and epithelial cells. **C** EGFR-activity scores were correlated with human cancer hallmark scores (*n* = 50; MSigDB) for HPV-neg. and -pos. epithelial cells. Shown are top 5 positively (red) and negatively (blue) correlated cancer hallmarks for HPV-neg. and -pos. epithelial cells (Pearson correlation). **D** EGFR signaling and EMT scores from malignant HNSCC cells (primary tumors and nodal metastases) are shown in a dot plot (Pearson correlations). **E** EGFR-activity subtypes identified by NMF from malignant HNSCC cells (primary tumor and nodal metastases) are depicted in a UMAP with tissue type of origin and patient ID. Examples of selective genes (AREG, EREG), rare genes (CBL), and ubiquitous genes (UBC) are depicted in lower UMAPs. **F** Heatmap representation of the gene expression of “REACTOME_SIGNALING_BY_EGFR” in EGFR-activity subtypes. **G** Heatmap of intensity scores of *n* = 13 PROGENy signaling pathways for all EGFR-activity subtypes

We hypothesized the existence of recurrence-promoting EGFR-activities impacting on therapy efficacy. EGFR-activities were determined by non-negative matrix factorization (NMF) of the “REACTOME_SIGNALING_BY_EGFR_IN_CANCER” gene set in the external HNSCC scRNA-seq dataset GSE181919 [16] (Fig. 6A). Cell types were attributed to tissue of origin (normal epithelia, leukoplakia, primary HNSCC, nodal metastases) and human papillomavirus (HPV)-status to extract HPV-neg. and -pos. epithelial cells (Fig. 6B). A higher positive correlation of EGFR-activity scores with EMT in HPV-neg. epithelial cells and a higher negative correlation with oxidative phosphorylation in HVP-pos. epithelial cells were observed (Fig. 6C). Differences in EGFR-activity correlations with EMT were accentuated in primary tumors and nodal metastases (Pearson HPV-neg._corr_ 0.42 vs. HPV-pos._corr_ 0.19) (Fig. 6D). NMF clustering of HPV-neg. epithelial cells from primary tumors and nodal metastases uncovered a subtype with low EGFR-activity (Basic EGFR) and subtypes with higher activity, determined by combinations of selectively expressed *HRAS* and/or EGFR ligands *AREG*, *EREG*, *HBEGF*, and *TGFA* (Fig. 6E, F). EGFR signaling was highest in the “AREG&HRAS&PTPRK”, “AREG&EREG&HRAS&HBEF”, “HBEGF&HRAS&AREG”, and “EREG&AREG&HBEGF&HRAS” subtypes (Fig. 6G).

**Fig. 7:**
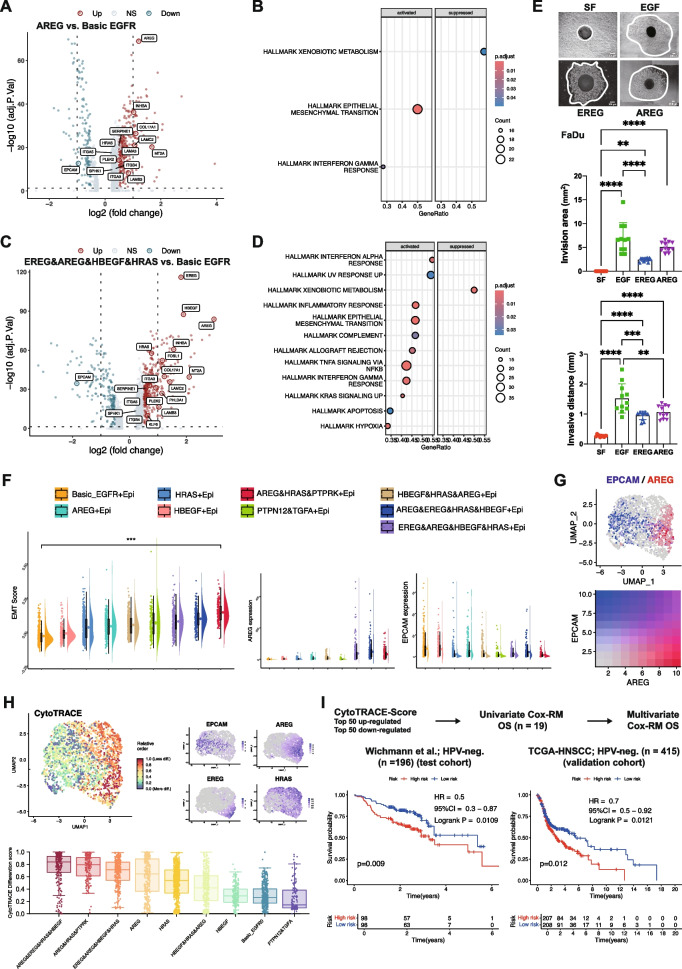
EGFR-activity subtypes regulate EMT and associate with overall survival. **A** , **C** Volcano plots with log2FC and *p*-value of DEGs from “AREG” versus “Basic EGFR” subtypes (**A**) and “EREG&AREG&HBEGF”HRAS” versus “Basic EGFR” subtypes (**C**). **B**, **D** Gene set variation analysis (GSVA) of “AREG” versus”Basic EGFR” (**B**) and “EREG&AREG&HBEGF”HRAS” versus”Basic EGFR” (**D**). Shown are significantly activated and suppressed human cancer hallmarks (MSigDB) with gene counts and adjusted *p*-values. **E** FaDu cell spheroids were embedded in type I collagen and treated with EGF, EREG, or AREG. Shown are representative examples from *n* = 3 independent experiments performed with 5–10 spheroids for each treatment and experiment. **F** Quantification of local invasion from *n* = 3 independent experiments with 5–10 spheroids each per treatment is shown as mean invasive areas and distances with SD. ** *p*-value < 0.01, *** < 0.001, **** < 0.0001. **F** EMT scores, *AREG* and *EpCAM* expression of EGFR-activity subtypes in malignant cells of GSE181919 are shown in violin plots. **G** UMAP and heatmap of *AREG* (red) and *EpCAM* (blue) expression in malignant cells of GSE181919. **H** CytoTRACE2 differentiation scores of malignant cells of GSE181919 and EGFR-activity subtypes are showns as UMAP and boxplot-whisker, respectively. Expression pattern of AREG, EpCAM, EREG, and HRAS are shown as UMAP. **I** Top 50 up- and down-regulated genes associated with CytoTRACE2 scores were analyzed by univariate Cox regression for correlation with OS. Correlated genes (*n* = 19) were implemented in a multivariate Cox regression model-based risk score for OS in HPV-neg. HNSCC-TCGA samples (*n* = 415, test cohort). Risk score was confirmed in the validation cohort (*n* = 196 HPV-neg. HNSCC). Shown are KM curves with HR, 95%-CI, *p*-value, and numbers at risk

Mild *AREG* upregulation in the “AREG” subtype induced several fDEGs including *INHBA*, *SERPINE1*, ITGA3, *COL17A1*, *LAM3*, *LAMB3*, *LAMC2*, and *MT2A* compared to the “Basic EGFR” subtype (Fig. 7A). Gene set variation analysis (GSVA) revealed EMT as major activated hallmark of the “AREG” subtype (Fig. 7B). Enhanced EGFR-activity of the “*EREG*&*AREG*&*HBEGF*&*HRAS*” subtype correlated with additionally induced fDEGs *PLEK2*, *PHLDA1*, *KLF6*, and *ITGB4* and hallmark pathways (Fig. 7C, D). Consequently, recombinant AREG induced local invasion of FaDu, OSC19, and HSC4 cells, whereas EREG promoted invasion in FaDu and HSC4 cells (Fig. 7E and Suppl. Figure 7A-D). Induction of local invasion by AREG and EREG was reflected by correlating levels of ERK1/2 activating phosphorylation (Suppl. Figure 7E-G), further corroborating a central role of pERK1/2 in EGFR-mediated invasion [10, 11]. *AREG* expression strength positively correlated with EMT scores of EGFR-activity subtypes, whereas epithelial marker EpCAM was negatively correlated (Fig. 7F), and both genes marked two distinct cell populations (Fig. 7G). CytoTRACE-inferred potency scores visualized cells in a differentiated state coinciding with *EPCAM* and less differentiated cells expressing *AREG*, *EREG*, and/or *HRAS* (Fig. 7H). CytoTRACE differentiation and EMT scores were concordant across EGFR-activity subtypes, with high CytoTRACE and EMT scores for “*AREG*&*EREG*&*HRAS*&*HBEGF*”, AREG&HRAS&PTPRK”, “*EREG*&*AREG*&*HBEGF*&*HRAS*” subtypes (Fig. 7F and H). The top 100 differential genes correlated to high CytoTRACE scores were extracted (Suppl. Figure 8A) and analyzed for univariate association with OS in an external HPV-neg. test cohort [20]. Nineteen correlated genes (Suppl. Figure 8B, C) were implemented in a multivariate Cox regression model-based risk score stratifying HPV-neg. HNSCC of the test and the TCGA-HNSCC validation cohorts (*n* = 196 and *n* = 415, respectively) (Fig. 7I). Although patients deemed at high-risk based on the expression of the *n* = 19 genes consistently showed reduced survival for the clinical end-points progression-free, disease-free, disease-specific survival and progression-free interval, differences were not statistically significant.

Fewer EGFR-activity subtypes were determined in HPV-pos. HNSCC of the external dataset GSE181919, which remained associated with *AREG*, *HBEGF*, *HRAS*, and *TGFA* expression (Suppl. Figure 9A, B). Subtypes “TGFA”, “AREG”, and “HBEGF” displayed strongest EGFR activity (Suppl. Figure 9B, C). HPV-pos. subtypes were neither characterized by substantial induction of fDEGs nor EMT and subtype “AAMP” was characterized by suppression of EMT (Suppl. Figure 9D). Thus, accumulated expression of AREG and EGFR ligands induces progressive EMT, fDEGs, an invGRN activation, and de-differentiation that prognosticates poor OS in HPV-neg. but not in HPV-pos. HNSCCs.

### EMT-dependent TME interactions

Non-malignant cells within the external dataset GSE181919 were annotated using common markers [29]. Cell compositions displayed increased proportion of IgG^+^ plasma B cells in HPV-neg. HNSCC, increased proportion of naïve CD4 T cells in HPV-pos. HNSCC, and increased plasmacytoid dendritic cells (pDCs) and inflammatory cancer-associated fibroblasts (iCAFs) in nodal metastases (Suppl. Figure 10A). CellChat-inferred ligand-receptor communication showed that EMT^high^ “AREG&HRAS&PTPRK” malignant cells were characterized by enhanced communication with each other, immune cells, monocytes, and DC compared to EMT^low^ “Basic EGFR” cells in HPV-negative tumors. Increased numbers and strength of inferred interactions of EMT^high^ malignant cells was observed with plasma B cells, proliferative CD4 and CD8 T cells, cytotoxic CD8 T cells, and pro-tumorigenic M2 macrophages (Suppl. Figure 10B, C). Selective interactions of EMT^high^ malignant cells occurred via Thrombospondin/CD47 with all B cell subtypes, all CD4 T cell subtypes except naïve, cytotoxic, naïve, and proliferative CD8 T cells, and all monocytes and DCs. A potential activation of CXCR3 by CXCL9, 10, and 11 was inferred for CD4 and CD8 T cells, conventional DCs (cDCs) and pDCs. Interaction of Poliovirus receptor cell adhesion molecule PVR with DNAX accessory molecule-1 CD226/DNAM-1 was seen with cDC1. Lastly, activation of NOTCH receptors 1–4 on various subtypes of CAFs by EMT^high^ malignant cell-derived Delta-like 1 and Jagged 2 ligands was suggested (Suppl. Figure 10D and 11).

“AREG”- and “HBEGF”-associated subtypes were identified in HPV-neg. and -pos. HNSCC, but only HPV-neg.-derived subtypes showed enhanced interactions amongst malignant cells and with B, CD4, and CD8 T cells, monocytes, DCs, and fibroblasts. Thrombospondin1/2-CD47 and PVR-TIGIT interactions that contribute to escape from the immune system as immune checkpoints [30, 31] were selectively observed in HPV-neg. but not HPV-pos. samples (Suppl. Figure 12).

### Sub-localization and association of fDEGs with Cetuximab response

**Fig. 8:**
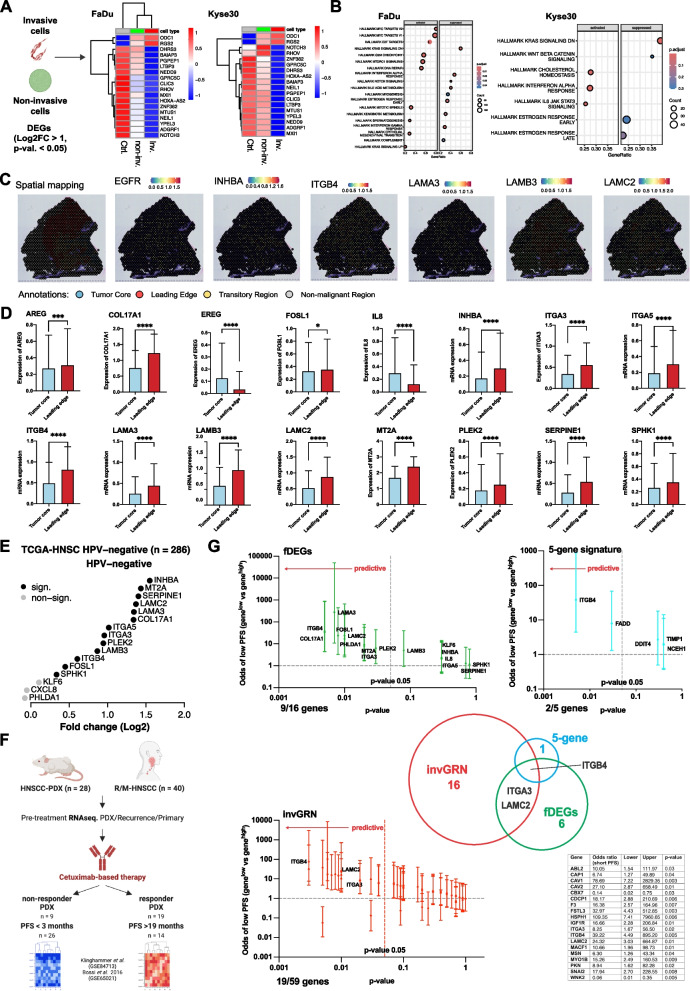
Expression of fDEGs correlates with response to Cetuximab. **A** Differential gene expression of invasive versus non-invasive cells (Log2FC > 1, *p*-value < 0.05) is shown as heatmaps for control, non-invasive, and invasive FaDu and Kyse30 cells. **B** GSEA of invasive versus non-invasive FaDu and Kyse30 cells is shown for human cancer hallmarks (MSigDB) with *p*-values and gene counts. **C** Spatial sub-localization of fDEGs was assessed in oral cavity squamous cell carcinoma (OSCC) in GSE208253. OSCC sections were classified into tumor core, leading edge, transitory region, and non-malignant region (Annotations). Shown is the representative expression of *EGFR*, *INHBA*, *ITGB4*, *LAMA3*, *LAMB3*, and *LAMC2* with expression intensities. **D** Tumor core- and leading edge-associated expression of significantly different fDEGs are shown as mean with SD of *n* = 10 OSCC patients.; *p*-values * < 0.05, ** < 0.01, *** < 0.001, **** < 0.0001. **E** Expression of *n* = 16 fDEGs was analyzed in budding and non-budding HPV-neg. HNSCC of the HNSCC-TCGA cohort *n* = 286). Shown are Log2FC of gene expression comparing budding with non-budding tumors. Black symbols = significant. Grey symbols = non-significant. **F** HNSCC patient-derived xenotransplants (PDX, *n* = 28) were subjected to RNA-seq. before transplantation and treatment with Cetuximab (GSE84713). Primary tumors or recurrences from recurrent/metastatic HNSCC-patients (R/M-HNSCC; GSE65021) were subjected to RNA-seq. before treatment with a Cetuximab-based regimen. PDXs were separated into Cetuximab-responders (*n* = 19) and non-responders (*n* = 9) based on tumor size. Patients were classified as short progression-free survival (PFS < 3 months; *n* = 26) and long PFS (> 19 months; *n* = 14). **G** Multivariate linear regression model estimating the odds for short versus long PFS in R/M-HNSCC-patients treated with Cetuximab. All fDEGs, genes of the invasive gene regulatory network (invGRN, see in Table), and the 5-gene signature are indicated with odds ratio to belong to the group of short progression-free survival (PFS), the range of odds, and *p*-value. Median split was chosen for gene expression. Common predictive genes are shown in a quantitative Venn diagram

Next, we addressed gene expression differences between invasive and non-invasive cells after EGF treatment in our 3D model. Differences were reflected by *n* = 18 common DEGs in FaDu and Kyse30 cells with a bias for downregulated genes in invasive cells (*n* = 16/18) (Fig. 8A). GSEA of invasive versus non-invasive cells identified two common suppressed hallmark pathways (HALLMARKS_KRAS_SIGNALING_DN, HALLMARK_ESTROGEN RESPONSE_EARLY), and activated EMT was seen in invasive FaDu cells, only (Fig. 8B). These findings suggested restricted differences after EGFR treatment in cells that actively invade versus spheroid-resident cells at the time point of sample collection. This pointed at a stochastic and/or time-dependent discrimination of invading versus non-invading cells after exposure to EGF in our 3D model. Spheroid size allowed a complete penetration of EGF and Cetuximab (see Fig. 1), thus not entirely mimicking gradients within larger tumors in vivo. Therefore, we used publicly available spatial transcriptomic (ST) data to interrogate sub-localizations of fDEGs in tumor core (TC), and leading edge (LE) of oral SCC (external dataset GSE208253 [18]), as exemplified for *ITGB4*, *LAMA3*, *LAMB3*, and *LAMC2* (Fig. 8C**)**. Significantly enhanced averaged expression in LE in *n* = 12 OSCC was observed for all fDEGs except *KLF6* and *PHPDLA1* (no significant difference), and *CXCL8* (IL8) (significantly reduced in LE) (Fig. 8D). Because the discrimination of leading edge and tumor core is based on marker gene expression rather than on invasive and metastatic capacities, we additionally investigated the expression of fDEGs in tumor budding, representing initial steps of local invasion mimicked in our 3D model [32]. In line with enhanced expression within the leading edge, 13/16 fDEGs were significantly enhanced in budding versus non-budding carcinomas of the HPV-neg. TCGA-HNSCC cohort (*n* = 286). Central genes of the invGRN, including *INHBA*, *LAMC2*, *LAMB3*, *ITGB4*, *ITGA5*, and *SPHK1*, were significantly associated with tumor budding, most strongly *INHBA*. Oppositely, *CXCL8*, *KLF6*, and *PHLDA1*, which were not enhanced in the LE, were not tumor budding-associated genes (Fig. 8E).

Next, we hypothesized that R/M-HNSCC patients with enhanced expression of fDEGs and EMT-associated EGFR-activity subtypes may benefit from Cetuximab. Cetuximab-treated patient-derived xenotransplants (PDX) (external dataset GSE84713 [21]) and R/M-HNSCC (external dataset GSE65021 [19]) were analyzed. Upon xenotransplantation and Cetuximab-treatment, PDX were categorized into responder (*n* = 19/28) and non-responder (*n* = 9/28). Cetuximab-treated R/M-HNSCC-patients showed either no signs of response with median progression-free survival (PFS) of three months (range 1–5 months) or response to treatment with median PFS 19 months (range 12–36 months) (Fig. 8F). *INHBA*, *ITGA3*, *ITGB4*, and *MT2A* were significantly upregulated in responder PDXs (AUC (ROC) values > 0.8) (Suppl. Figure 13). *AREG*, *COL17A1*, *EREG*, *FOSL1*, *ITGA3*, *ITGB4*, *LAMA3*, *LAMC2*, *MT2A*, *PHLDA1*, and *PLEK2* were upregulated in R/M-HNSCC-patients with long PFS, (AUC (ROC) 0.681–0.879) (Suppl. Figure 14A). After adjustment in a multivariate linear regression, low expression of *COL17A1*, *FOSL1*, *ITGA3*, *ITGB4*, *LAMA3*, *LAMC2*, *MT2A*, *PHLDA1*, and *PLEK2* was associated with significantly increased odds of low PFS (Fig. 8G**, **Suppl. Figure 14B, and Suppl. Table 3). We extended the identification of predictive markers to all genes comprised in the invGRN and to an EGFR-EMT-derived prognostic 5-gene signature [11]. N = 19/59 and n = 2/5 predictive genes were identified in the invGRN and the 5-gene signature, respectively. ITGB4 was determined as the predictive gene common to all three signatures, and the invGRN and fDEGs further shared ITGA3 and ITGB4 ligand LAMC2 (Fig. 8G and Suppl. Table 3). Thus, low expression of fDEGs, of genes of the invasive gene regulatory network, and of genes of EGFR-EMT-dependent 5-gene prognostic signature was associated with reduced response to Cetuximab in R/M-HNSCC patients.

## Discussion

Unlike targeted therapy protocols instructed by predictive companion biomarkers (*e.g.* Herceptin/HercepTest), Cetuximab-based therapy of R/M-HNSCC occurs without prior molecular stratification, resulting in poor overall response rates below 15% [33]. Hence, the potential of targeted therapies has not been fully realized despite an obvious oncogene addiction to EGFR. A rationale for a lack of response can be seen in the diversity of EGFR signaling and entailed phenotypes impacting on response to therapy. Empirical data revealed that EGFR expression levels do not predict response to Cetuximab in R/M-HNSCC [34]. Proteogenomic analyses demonstrated that ligand abundance dictates response to inhibitors by controlling EGFR activation, in accordance with an impact of EGFR-signaling outcome on treatment response [35]. However, it is known that EGF has dual capacity to induce proliferation and EMT, depending on strength and duration of the activation [10]. As described by Bossi et al*.*, response to Cetuximab in R/M-HNSCC-patients was associated with the basal molecular subtype, pronounced EGFR signaling, and hypoxia [19]. The authors defined differentially expressed genes that separated responders and non-responders and identified the gene sets “Ectoderm/Epidermis” differentiation, “Defense response”, “EGFR signaling pathway” and “Protein catabolism” in responders. Oncosignature mining revealed strongest correlations of non-responders to KRAS signaling and of responders to beta-catenin, E2F3, cMYC, and p53 pathways, which were confirmed in an isogenic pair of cetuximab-resistant and -sensitive cell lines [19]. Initial work by our group has identified a gene expression signature of EGFR-mediated EMT in 2D cell culture. This expression profile contains a 5-gene signature with prognostic value and actionable targets ITGB4 and CD73 [11, 13]. Hence, multi-faceted functionalities regulated by EGFR signaling contribute differently to tumor progression and impact on therapy response, thereby providing valuable avenues for treatment of advanced HNSCC.

Consequently, the present study focused on the molecular characterization of the functional link between EGFR-mediated EMT and tumor cell dissemination as a starting point to derive EGFR-entailed phenotypes predicting Cetuximab response. For this, a 3D model of local invasion in extracellular matrix was combined with photoconvertible and traceable fluorescence marker Dendra2, a sensitive and specific tracer of tumor cell dissemination [15]. After tracer-based cell enrichment, 3´-RNA-seq served to measure transcriptomic changes across samples. Although 3´-RNA-seq does not allow for the resolution of alternative splice variants, a key advantage is its ability to generate reliable transcriptomic data from minimal input material (< 50 ng total RNA), as required for restricted numbers of invasive cells. Using this model, we formally confirmed a link between EGFR-mediated EMT and local invasion and identified actionable genes controlling dissemination (fDEGs) after cross-referencing with external scRNA-seq datasets and HNSCC cohorts. Integrating 3’-RNA-seq data with whole RNA-seq datasets from public repositories is straightforward, when analyzed at the gene level, which both approaches quantify comparably. Additionally, many analyses rely on transcriptome-derived gene summarization methods, such as GSEA/GSVA, which translate gene expression into pathway or signaling activity scores [36]. These scores are generally robust across different RNA-seq protocols, ensuring compatibility between datasets.

We describe a novel EGFR-controlled gene regulatory network instrumental in invasive cells. This invGRN comprises fDEGs and is activated by subtypes of EGFR-activity that are primarily governed by EGFR ligands *AREG*, *EREG*, and *HBEGF* in single tumor cells. These findings corroborated the importance of ligand availability [35] and describe for the first time an impactful heterogeneity of EGFR signaling at the level of single malignant cells. EGFR-activity subtypes characterized by accumulating expression of *AREG* and concurrently reduced expression of epithelial marker *EpCAM* promote de-differentiation towards EMT, local invasion, tumor budding, and are correlated to worsened OS. Upregulation of fDEGs including central genes of the invGRN in primary cancers characterized by tumor budding is of particular interest since advanced OSCC patients with single invading tumor cells were at increased risk of local recurrence [37] and because tumor budding manifestation is an independent biomarker of HNSCC [24].

*INHBA* emerged as a central regulator, controlling numerous genes including *AREG*, *LAMB3*, and *LAMC2*. Blocking activin A suppressed local invasion, which was reverted by treatment with LN5 and S1P. These complementation experiments provided evidence for the interconnected functionality of the invGRN in controlling local invasion. Recent studies of HPV-negative and -positive oropharyngeal squamous cell carcinoma (OPSCC) disclosed *INHBA* as inducer of EMT, migration, proliferation, stemness, and resistance to cell death [38], as well as a multidrug chemoresistant gene in HNSCC [39]. The identification of *ITGB4* and genes composing its matrix-resident ligand laminin 5 (LN5: LAMA3, B3, C2) as fDEGs in invasive cells and as central components of the invGRN offers molecular insight into our initial description of an EGFR-EMT signature-derived risk score for HNSCC comprising ITGB4 [11]. We describe a novel ITGB4-independent function of LN5 in rapidly activating EGFR towards ERK1/2 phosphorylation, fostering local invasion induced by EGF. We thus propose LN5 as a potential novel EGFR ligand in HNSCC, in accordance with comparable functions of LAMC2 in other solid cancers [40, 41].

Inhibition experiments suggested synthetic effects of SPHK1 and Cetuximab in combinatorial treatments, in line with a capacity to increase sensitivity towards Cetuximab [42]. The identification of candidates for co-treatment becomes increasingly relevant as effects of dual inhibitors have been recently reported for EGFR/PI3K targeting in HNSCC. EGFR/PI3K single-molecule inhibitor MTX-531 showed high potency as monotherapy in HNSCC-derived PDX. Additional combination with MAPK or KRAS-G12C inhibitors further potentiated its beneficial effects [43], which is in line with potent effects of MEK inhibitor AZD6244 on EGFR-mediated EMT and local invasion shown in the present work. Combinations of EGFR-targeting drugs with inhibitors of MAPK and fDEGs such as ITGB4 and SPHK1 represent promising candidate approaches to be addressed in future studies.

p-EMT is preferentially induced at the interphase of tumor and stroma [5, 11], where the expression of fDEGs was enhanced. In combination with a significantly enhanced expression of fDEGs in budding tumors, these findings suggest a central role for tumor cells in p-EMT and resident in peripheral areas during initial steps of local dissemination and immune cell suppression. Arora et al*.* emphasized enhanced EGFR activity and cell–cell communication of malignant cells via LAMB3-ITGA6:ITGB4 at the leading edge [18]. We suggest that these interactions are fostered by EMT-related EGFR-activity subtypes promoting central ligand-receptor (L-R) pairs. Accordingly, in silico analyses revealed that EMT^high^ EGFR-activity subtypes undergo more interactions of higher strength with malignant cells, immune cells, and subtypes of CAFs. L-R interactions selectively observed with EMT^high^ EGFR-activity subtypes were part of immune escape mechanisms, such as thrombospondin 1 and 2 ligation of checkpoint molecule CD47 and PVR-CD226/TIGIT interactions [31, 44, 45]. Blockade of CD47 on tumor cells improved response to Cetuximab and radiation and showed efficacy in combination with Pembrolizumab in advanced solid tumors [46–50]. Accordingly, CD47-PD-L1-bispecific antibody PF-07257876 has entered a Phase I clinical trial for patients suffering from HNSCC, non-small cell lung cancer (NSCLC), and other PD-L1-positive carcinomas refractory and naïve to ICI (NCT04881045). Initial results demonstrated good tolerability and partial responses in HNSCC [48]. Activation of CXCR3 by CXCL9, 10, and 11 was reported to promote lympho-vascular invasion, metastasis formation, and EMT [51, 52], and may thus contribute to worsened survival of patients with EMT^high^ EGFR-activity in the absence of EGFR blockade. Overall, EMT-associated EGFR-activity subtypes provide HPV-neg. HNSCC cells with traits of local invasion, immune escape, and worsened OS. This notion is supported by the finding that *AREG*- and *HBEGF*-driven EGFR-activity subtypes identified in HPV-pos. samples showed comparably reduced immune-suppressive L-R communications, along with significantly improved OS.

It must however be noted that the present transcriptomic study focused on malignant cells and the impact of EGFR signaling on local invasion. Interactions with immune and stromal cells within the tumor microenvironment have been addressed solely in silico. Recently published work by the Wickström group delineated phenotypic signatures and tumor cell-stroma interactions correlated with metastases and recurrence in HNSCC. Of particular interest, malignant cells in p-EMT were shown to be primed for further induction towards invasion through cell–cell interaction with CAFs. Induction of an invasive phenotype selectively in p-EMT tumor cells depended on the activation of EGFR by AREG on fibroblasts [53]. Hence, tumor cell-intrinsic autocrine/paracrine capacities described in the present work and interaction with non-malignant cells seemingly converge at the level of EGFR ligands to govern local invasion. The resulting EGFR activity subtypes and entailed phenotypes, which differentially contribute to tumor progression, provide molecular rationales for varying patients´ responses to treatment. Accordingly, enhanced expression of numerous fDEGs was associated with the response to Cetuximab treatment in a PDX mouse model and in R/M-HNSCC-patients. Central invGRN regulator INHBA was associated with response to Cetuximab in PDX but not in R/M-HNSCC. A molecular explanation is yet to be given, but it is conceivable that INHBA-induced downstream effector molecules that substitute Activin A functions in EGFR-mediated invasion, represent better predictive markers. Significant correlation of INHBA targets ITGB4, LAMA3 and C2, and a strong tendency of AREG to predict response to treatment in R/M-HNSCC patients was observed. Cross-comparison with the 5-gene prognostic signature and genes composing the invGRN confirmed ITGB4 as common predictive gene. Together with the observation that ITGB4 was the only gene of the 5-gene signature also identified as fDEG and component of the invGRN, the current data suggest a central role for ITGB4 in the complex re-programming of tumor cells towards local invasion and in response to EGFR-based treatment. In conclusion, we propose that high expression of fDEGs characterizes primary HNSCC and recurrences with a dependency from EGFR-activity subtype-driven tumor progression. Accordingly, Cetuximab treatment of this subgroup of R/M-HNSCC patients has a beneficial therapeutic impact as it suppresses progression-relevant aspects of EGFR signaling.

### Limitation of the study and outlook

Translation of the present findings into predictive clinical tests for treatment response remains a key challenge. A consensus panel of genes derived from EGFR-associated progression pathways (*e.g.* fDEGs, invGRN, EGFR-related EMT) represents a promising predictive marker but cannot be assessed using sub-genomic panel sequencing, the current standard in routine molecular diagnostics for cancer patients. However, with the rise of clinical multi-omics, integrating gene expression-based biomarkers into precision oncology is becoming increasingly feasible at major comprehensive cancer centers. Crucially, these biomarkers must be validated in observational and, later, interventional clinical trials. Finally, the identified biomarkers of cetuximab response could drive the development of novel EGFR-targeted therapies, such as the bifunctional fusion antibody BCA101 [1].

## Conclusions

In summary, we present a comprehensive molecular landscape of EGFR-mediated local invasion including regulatory EGFR-activity subtypes, therapeutic target candidates, and predictive markers, which will fuel novel pre-clinical and clinical studies.

## Supplementary Information

Supplementary Material 1.

Supplementary Material 2.

Supplementary Material 3.

Supplementary Material 4.

Supplementary Material 5.

Supplementary Material 6.

Supplementary Material 7.

## Data Availability

Datasets are publicly available (TCGA [47], Wichmann et al. (GSE65858), FHCRC (GSE41613), Puram et al. (GSE103322), Choi et al. (GSE181919), Arora et al. (GSE208253), Bossi et al. (GSE65021), Klinghammer et al. (GSE84713), Quah et al. (GSE188737), or are deposited at GEO (GSE273463). All codes and R-packages used in the study are publicly available and have been disclosed in Methods or are available from the corresponding authors on reasonable request.

## Abbreviations

AKTi: Rac-alpha serine/threonine-kinase inhibitor
ANOVA: Analysis of variance
AREG: Amphiregulin
CPS: Combined positive score
DEG: Differentially expressed gene
ECM: Extracellular matrix
EGF: Epidermal growth factor
EGFR: Epidermal growth factor receptor
EMT: Epithelial-to-mesenchymal transition
EMT-TFs: EMT transcription factors
EREG: Epiregulin
Erk1/2: Extracellular regulated kinase 1/2
fDEGs: Functional DEGs
FGFR: Fibroblast growth factor receptor
FHCRC: Fred Hutchinson cancer research center
GEO: Gene expression omnibus
GSEA: Gene set enrichment analysis
GSVA: Gene set variation analysis
HB-EGF: Heparin-binding EGF like growth factor
HNSCC: Head and neck squamous cell carcinoma
HPV: Human papillovirus
INHBA: Inhibin subunit beta A
invGRN: Invasion gene regulatory network
ITGA5: Integrin alpha 5
ITGB4: Integrin beta 4
LE: Leading edge
LN5: Laminin 5
L-R: Ligand-receptor
MEKi: Mitogen-activated protein kinase kinase inhibitor
MSigDB: Molecular signature database
NFκB: Nuclear factor kappa B
NMF: Non-negative matrix factorization
NSCLC: Non-small cell lung cancer
OS: Overall survival
PDX: Patient-derived xenotransplant
PI3K: Phosphoinositide 3-kinase
R/M-HNSCC: Recurren/metastatic HSNCC
S1P: Sphingosine-1-phosphate
scRNA-seq: Single cell RNA sequencing
SNAI2: Snail family transcriptional repressor 2
SPHK1: Sphinogsin kinase 1
TB: Tumor budding
TC: Tumor core
TCGA: The cancer genome atlas
TGFβ: Transforming growth factor beta
TME: Tumor microenvironment
TNF: Tumor necrose factor
WGCNA: Weighted gene co-expression network analysis
