# Supplementary material for: EGFR-mediated local invasiveness and response to Cetuximab in head and neck cancer
Source: Mol Cancer. 2025 Mar 22;24:94. doi: 10.1186/s12943-025-02290-1 (PMC11929204; doi:10.1186/s12943-025-02290-1)
Supplement: Supplementary file 1 — Supplementary Material 1. [file 12943_2025_2290_MOESM1_ESM.docx]

| **Supplementary Table 2: TCGA HPV.neg HNSCC patients characteristics** | | | |
| --- | --- | --- | --- |
| **Clinical parameters** | **Variable** | **Total (415)** | **Percentages (%)** |
| Gender | Female | 123 | 29.6% |
|  | Male | 292 | 70.4% |
| T stage | T0 | 158 | 38.1% |
|  | T1 | 37 | 8.9% |
|  | T2 | 104 | 25.1% |
|  | T3 | 85 | 20.5% |
|  | T4 | 154 | 37.1% |
|  | Unknow | 34 | 8.2% |
| N stage | N0 | 151 | 36.3% |
|  | N1 | 56 | 13.5% |
|  | N2 | 137 | 33.0% |
|  | N3 | 65 | 15.7% |
|  | Unknow | 12 | 2.9% |
| M stage | M0 | 161 | 38.8% |
|  | M1 | 1 | 0.2% |
|  | Unknow | 253 | 61.0% |
| TNM stage | Stage I | 23 | 5.5% |
|  | Stage II | 58 | 14.0% |
|  | Stage III | 67 | 16.1% |
|  | Stage IV | 225 | 54.2% |
|  | Unknow | 42 | 10.1% |
| Survival status | Death | 191 | 46.0% |
|  | Alive | 224 | 54.0% |
