# Supplementary material for: EGFR-mediated local invasiveness and response to Cetuximab in head and neck cancer
Source: Mol Cancer. 2025 Mar 22;24:94. doi: 10.1186/s12943-025-02290-1 (PMC11929204; doi:10.1186/s12943-025-02290-1)
Supplement: Supplementary file 6 — Supplementary Material 6. [file 12943_2025_2290_MOESM6_ESM.pdf]

# Supplementary Figures

# EGFR-mediated Local Invasiveness and Response to Cetuximab in Head and Neck Cancer

*Jiefu Zhou[1,2,3,4,*], Min He[1,*], Qiong Zhao[1,*], Enxian Shi[1,*], Hairong Wang[1], Vaidehi Ponkshe[1], Jiahang Song[1], Zhengquan Wu[1], Dongmei Ji[5], Gisela Kranz[1], Anna Tscherne[1], Sabina Schwenk-Zieger[1], Nilofer Abdul Razak[1], Julia Hess[6,7], Claus Belka[7,8], Horst Zitzelsberger[6], Iordanis Ourailidis[9], Fabian Stögbauer[10], Melanie Boxberg[10], Jan Budczies[9], Christoph A. Reichel[1], Martin Canis[1,8], Philipp Baumeister[1,8], Hongxia Wang[5], Kristian Unger[7], Andreas Mock[8], and Olivier Gires[1]*

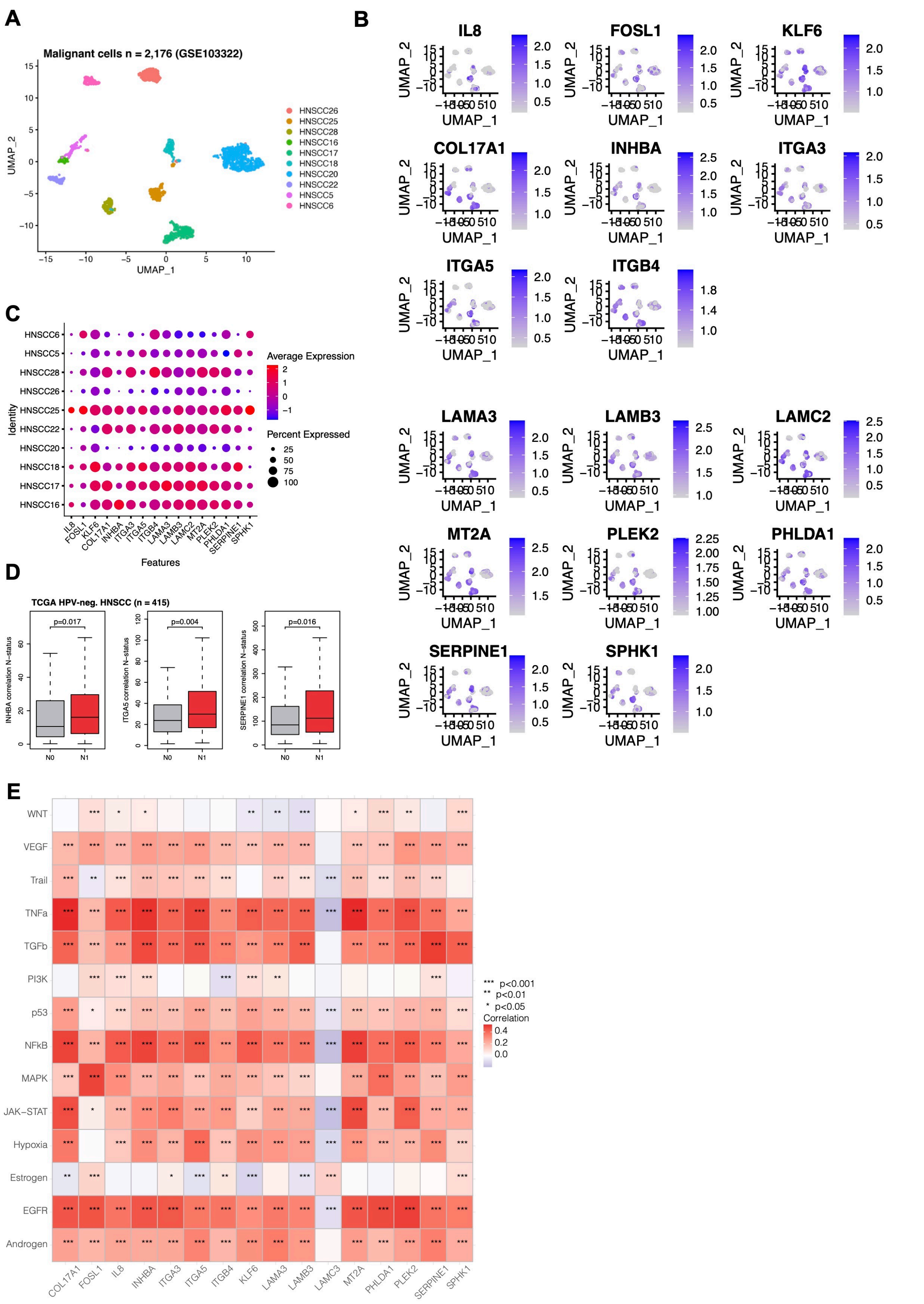


**Suppl. Figure 1: Expression of fDEGs in GSE103322**
(**A**) UMAP of malignant cells (n = 2,176) from n = 10 OSCC patients (GSE103322). (**B**) UMAP representation of n = 16 fDEGs in malignant cells of GSE103322. (**C**) Dot plot of fDEG expression in n = 10 OSCC patients (GSE103322) showing average expression and percent of positive single cells. (**D**) Bloxplot-whiskers graphs of the expression of *INHBA*, *ITGA5*, and *SERPINE1* in nodal metastases-

negative (N0) and -positive (N1) patients of the HPV-neg. HNSCC-TCGA cohort. (**E**) Heatmap of correlations of n = 16 fDEGs with signaling pathways within PROGENy with correlation and p-values.

**A**

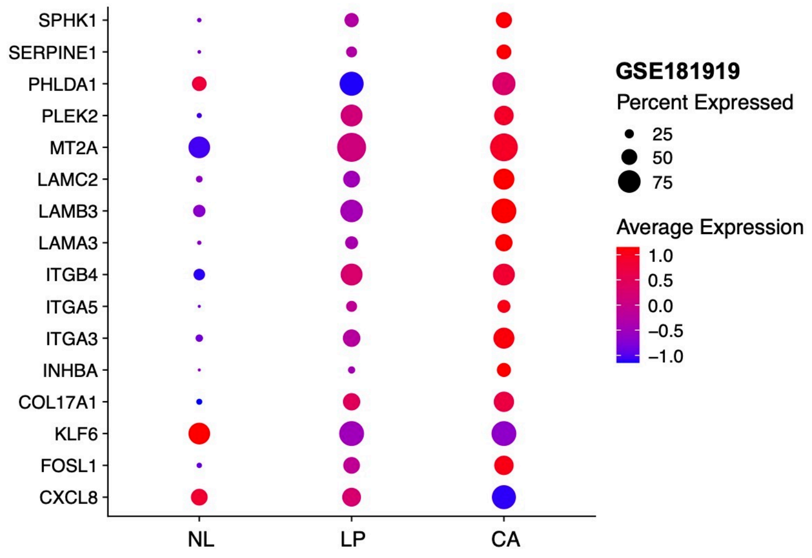


**B** **Normal**

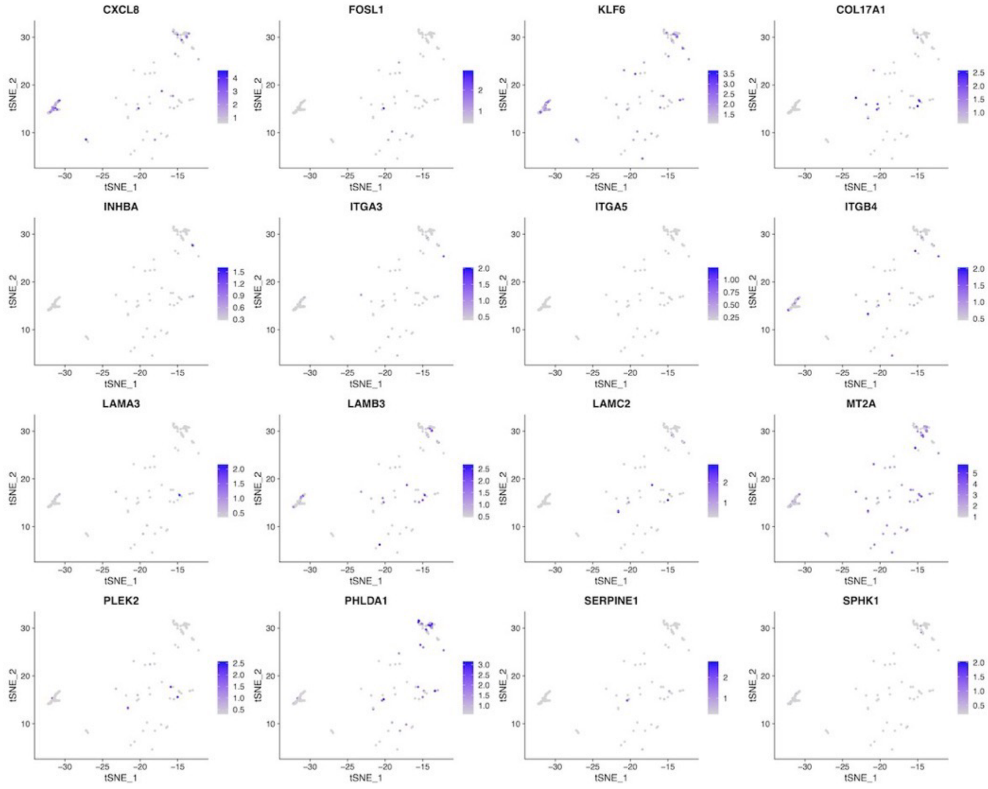

**C** **Leucoplakia**

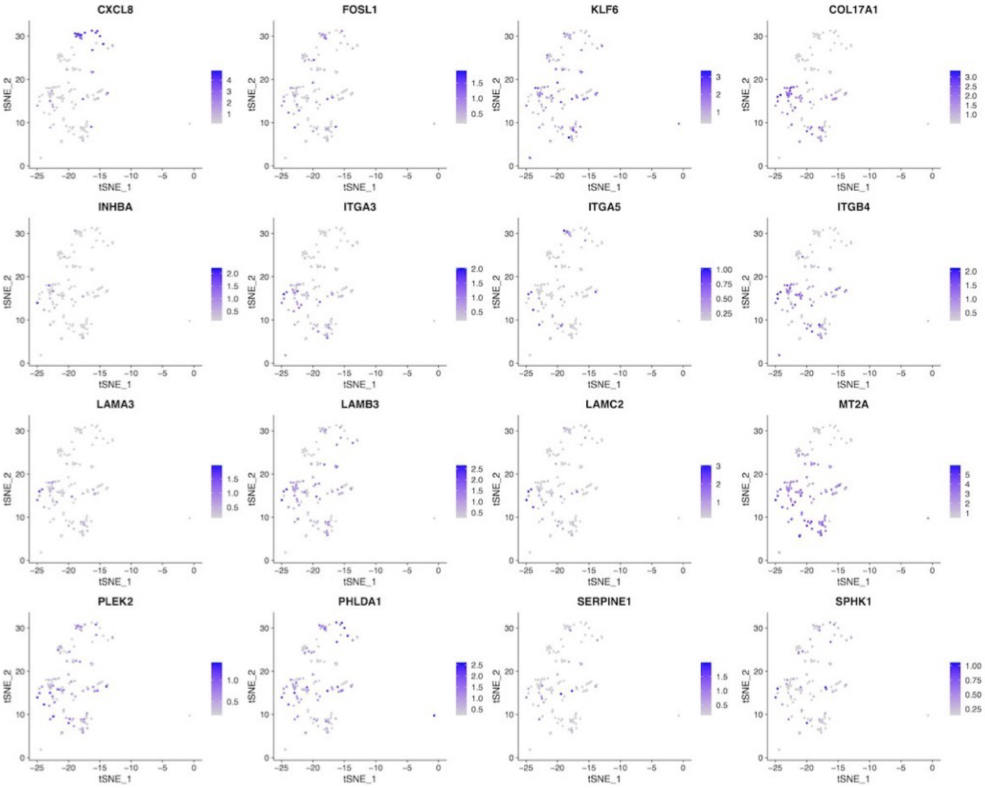


**D** **Carcinoma**

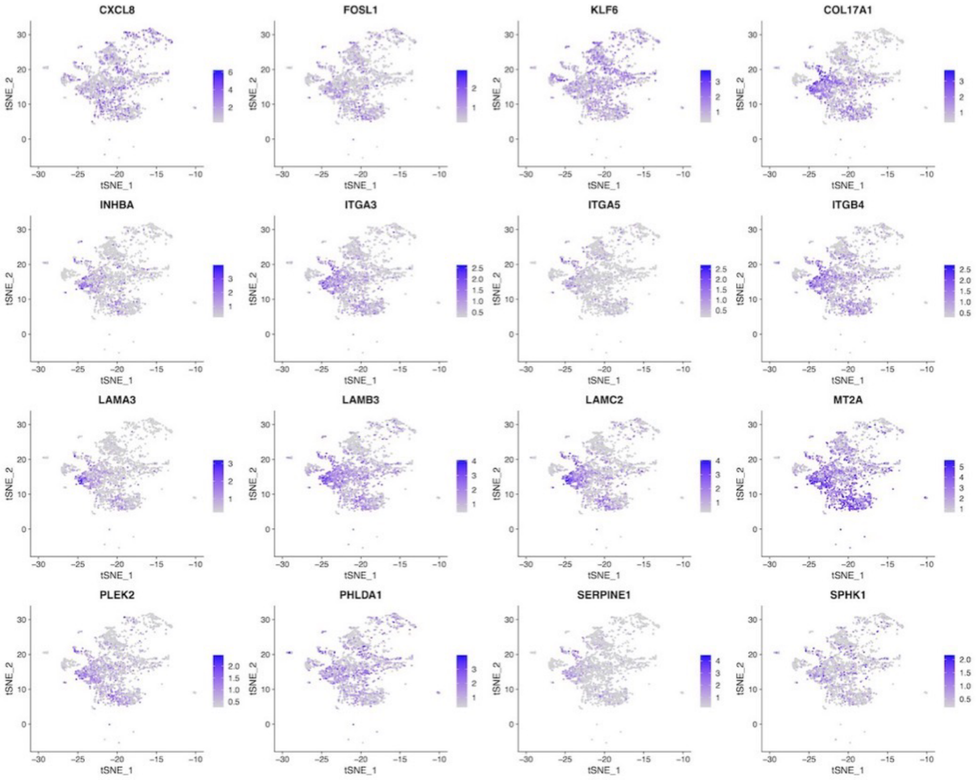

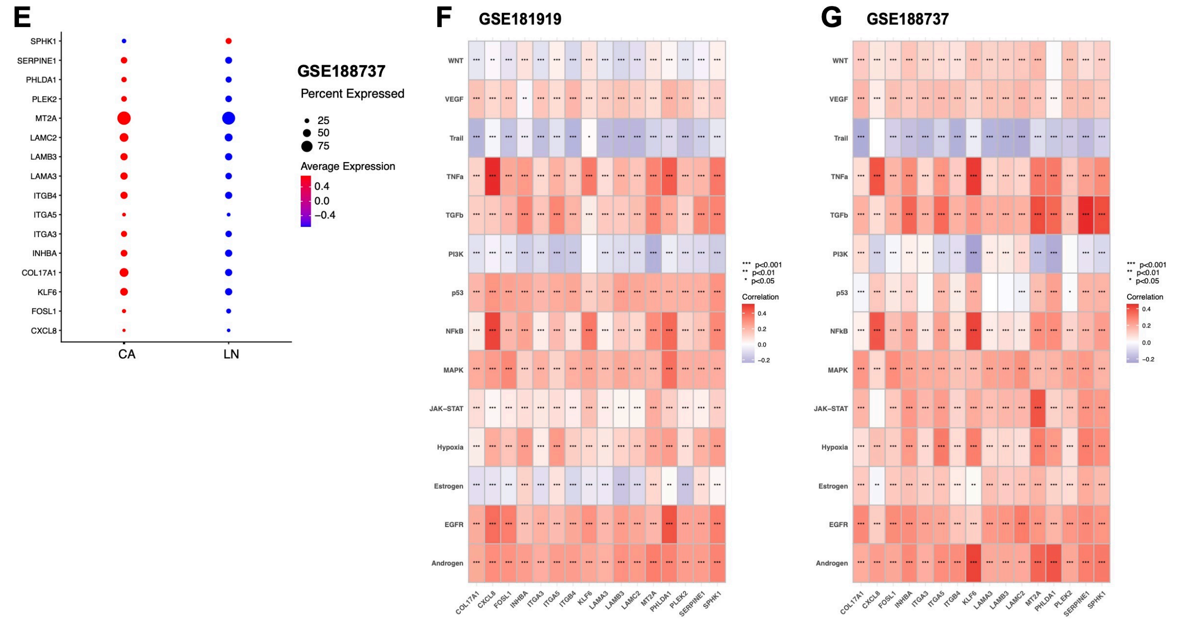


**Suppl. Figure 2: Expression of fDEGs in GSE181919**

(**A**) Dot plot of fDEGs expression in normal epithelial cells (NL), leucoplakia (LP), and primary HNSCC (CA) (GSE181919) showing average expression and percent of positive single cells. (**B-D**) UMAP representation of n = 16 fDEGs in normal epithelial cells (B), leucoplakia (C), and malignant cells (D). (**E**) Dot plot of fDEG expression in primary HNSCC (CA) and nodal metastases (LN) (GSE188737) showing average expression and percent of positive single cells. (**F, G**) Heatmap of correlations of n = 16 fDEGs with signaling pathways within PROGENy with correlation and p-values from GSE181919 (F) and GSE188737 (G).

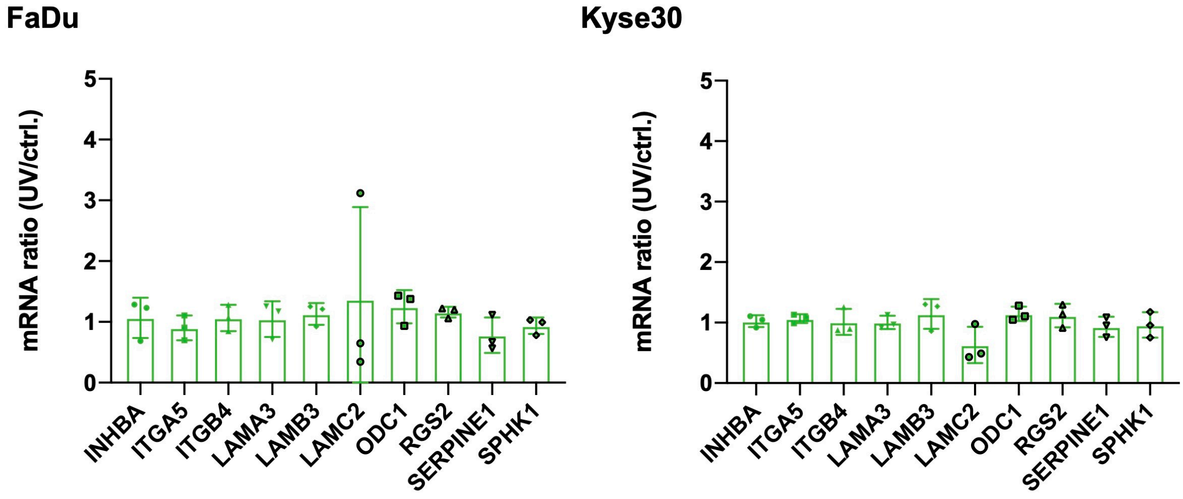


**Suppl. Figure 3: Effects of UV-irradiation on gene expression of selected functional differentially expressed genes (fDEGs)**

FaDu-Dendra2 and Kyse30-Dendra2 cells were grown as spheroids embedded in type I collagen and entire spheroids were treated with blue light in the DAPI channel (Leica DMi8 microscope system) in the absence of any further treatment. Cells in target areas were exposed to the following settings: FIM 100%, IL-Fld 2 round, time interval (2 secs), exposure (5 secs), cycle (4 times). Non-irradiated spheroids served as controls (ctrl.). Photoconverted red cells were sorted by FACS and the expression of the indicated genes was assessed by qRT-PCR. Shown are mean ratios with SD of delta-Ct values of UV-treated versus control samples from n = 3 independent experiments performed in triplicates.

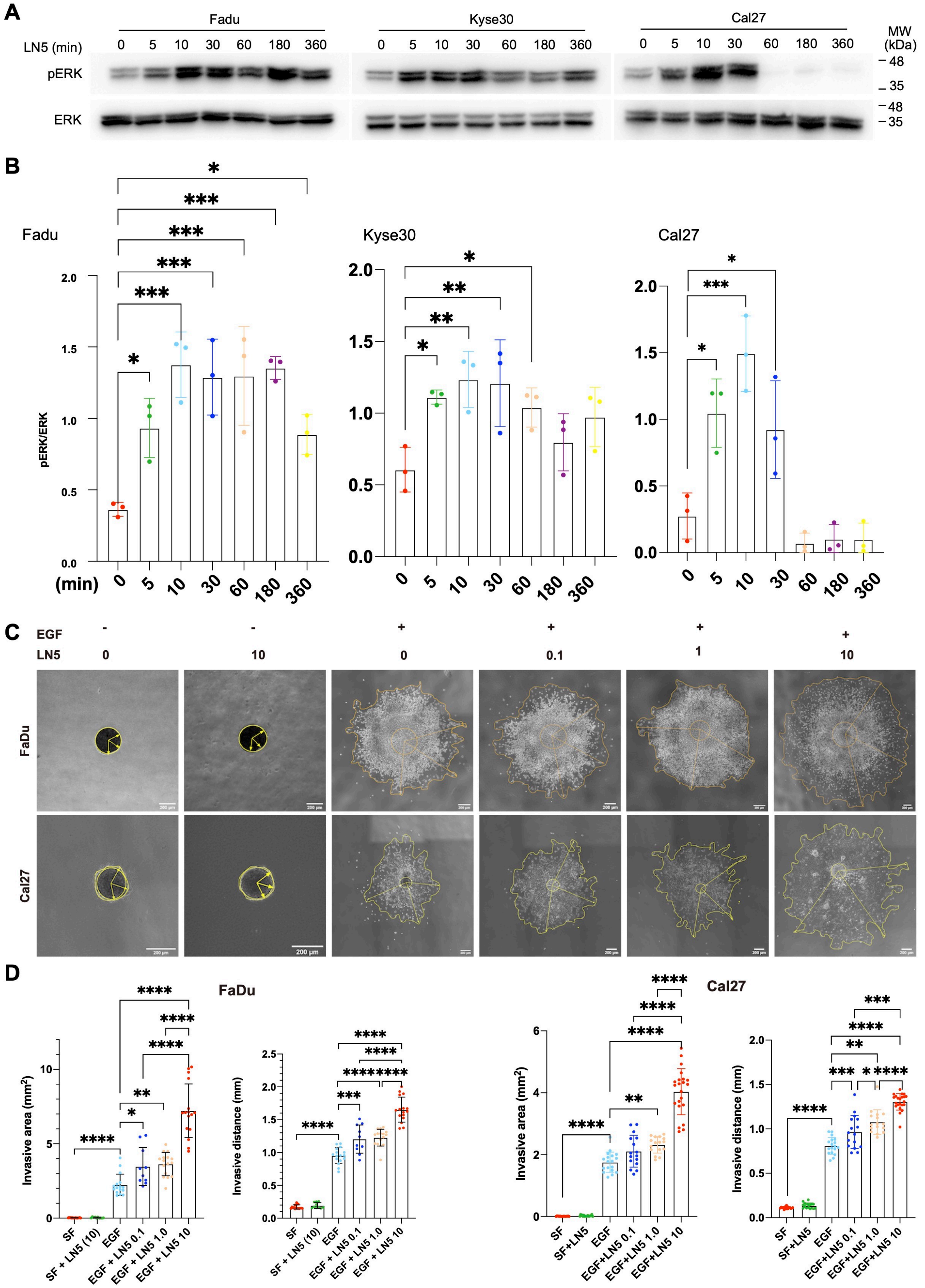


**Suppl. Figure 4: Kinetics of LN5-induced ERK1/2 phosphorylation**

(**A-B**) FaDu, Kyse30, and Cal27 cells were treated with laminin 5 (LN5) from 0-360 min. Whole cell extracts were separated by immunoblot and phosphorylated ERK (pERK) and ERK1/2 detected with specific antibodies. Shown are representative immunoblots from n = 3 independent experiments in (A). Quantification of n = 3 independent immunoblots as mean with SD and statistical significance is shown in (B). * p-value < 0.05, ** 0.01, *** 0.001, **** 0.0001. (**C**) FaDu and Cal27 cells were grown as

spheroids, embedded in type I collagen with or without increasing concentrations of laminin 5 (LN5), and treated with EGF as indicated. Shown are representative spheroids after 72 h of treatment with invasive area and distance from n = 3 independent experiments with 5-10 spheroids each per treatment. (**D**) Quantification of invasive area and distance from n = 3 independent experiments with 5-10 spheroids per treatment. Shown are mean invasive areas and distances with SD for FaDu (left) and Cal27 cells (right). ** p-value < 0.01, *** < 0.001, **** < 0.0001.

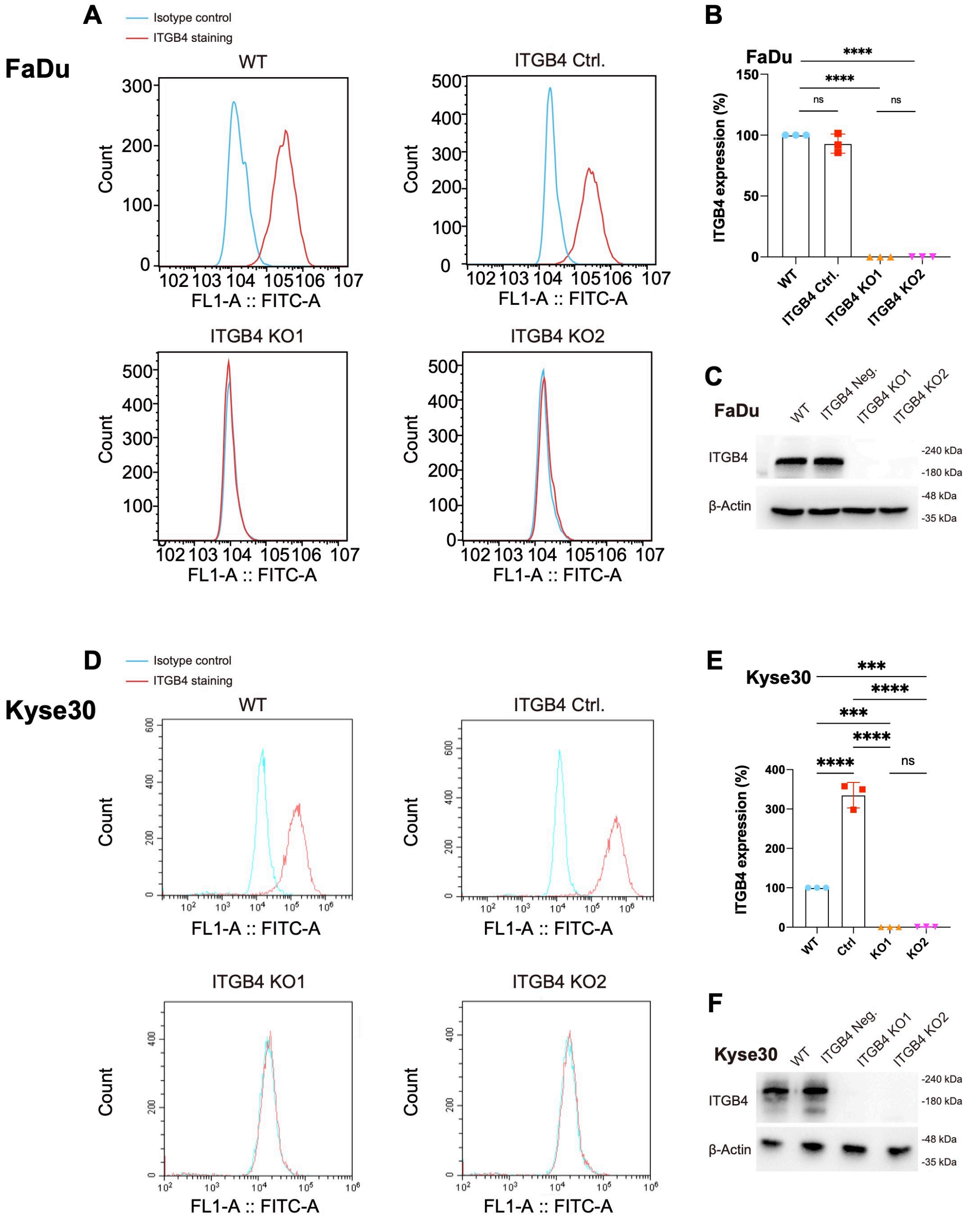


**Suppl. Figure 5: *ITGB4* knockout in FaDu cells**

(**A, B, D, and E**) ITGB4 expression was analyzed by flow cytometry in FaDu and Kyse30 wildtype cells (WT) and CRISPR-Cas9-mediated ITGB4 knockout clones including one ITGB4-positive control (ITGB4-Ctrl.) and two ITGB4-K.O. clones (ITGB4 KO1 and 2). Shown are representative histograms in (A) and (D) and mean fluorescence intensity (MFI) mean values with SD from n = 3 independent experiments in (D) and (E). (**C, F**) ITGB4 expression was analyzed by immunoblotting with specific antibodies in FaDu and Kyse30 WT, ITGB4-Ctrl., ITGB4-KO1 and -KO2. Shown are representative immunoblots from n = 3 independent experiments for FaDu (C) and Kyse30 (F).

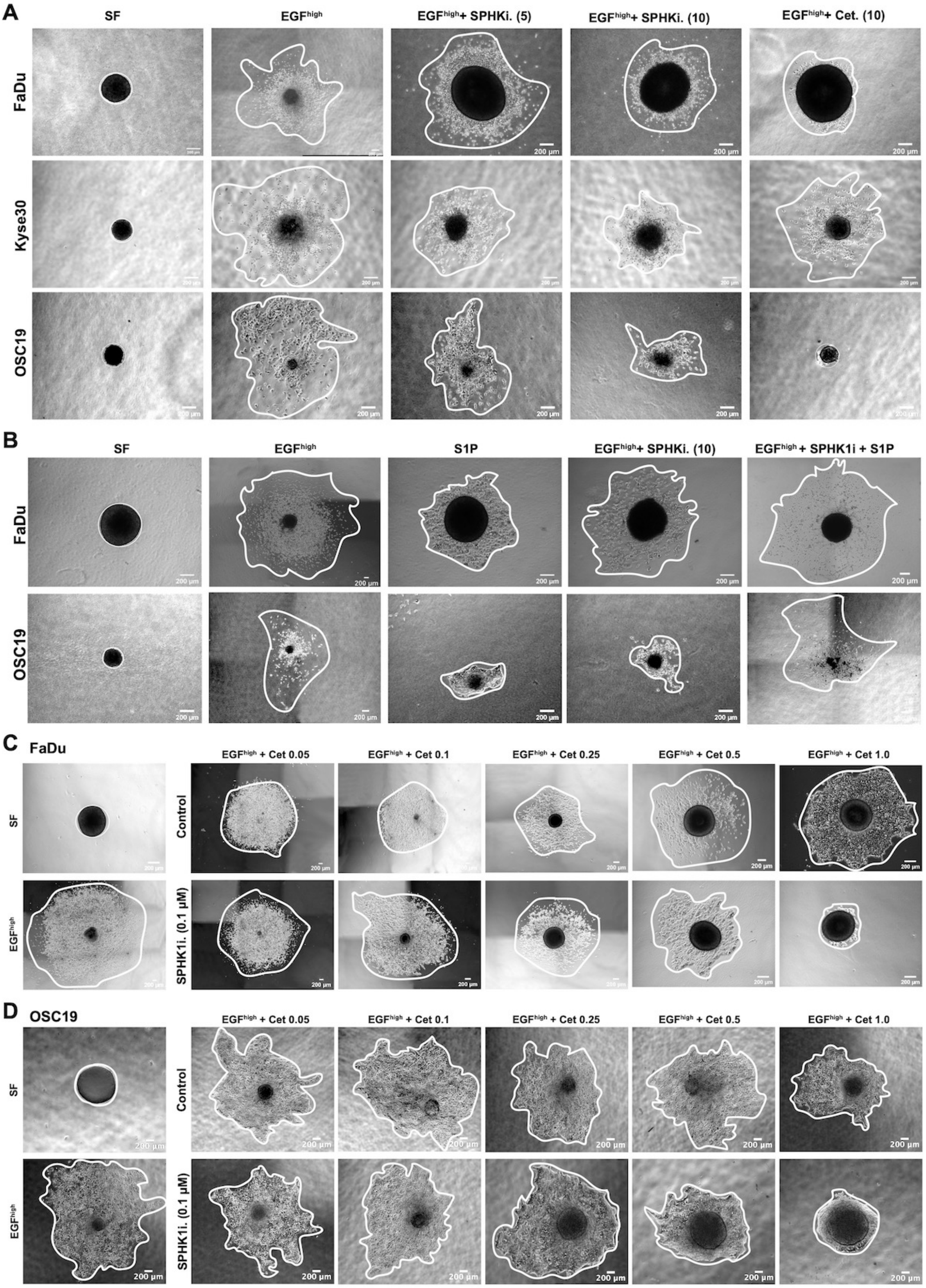


**Suppl. Figure 6: Effects of SPHK1 inhibition on local invasion**
(**A**) Fadu, Kyse30, and OSC-19 cell spheroids were embedded in type I collagen and treated with EGF (9 nM) in the absence or presence of SPHK1 inhibitor (5 and 10 µg/mL) and Cetuximab (10 µg/mL). Shown are representative pictures from n = 3 independent experiments with 5-10 spheroids per treatment and experiment. (**B**) FaDu and OSC-19 cell spheroids were embedded in type I collagen and treated with EGF, sphingosine-1-phosphate (S1P), and SPHK1 inhibitor in the indicated combinations.

Shown are representative examples from n = 3 independent experiments performed with 5-10 spheroids for each treatment and experiment. (**C-D**) FaDu (C) and OSC19 spheroids (D) were embedded in type I collagen and treated with EGF in combinations with Cetuximab (0.05-1.0 µg/mL) and SPHK1 inhibitor (SPHK1i) as indicated. Shown are representative examples from n = 3 independent experiments performed with 5-10 spheroids for each treatment and experiment.

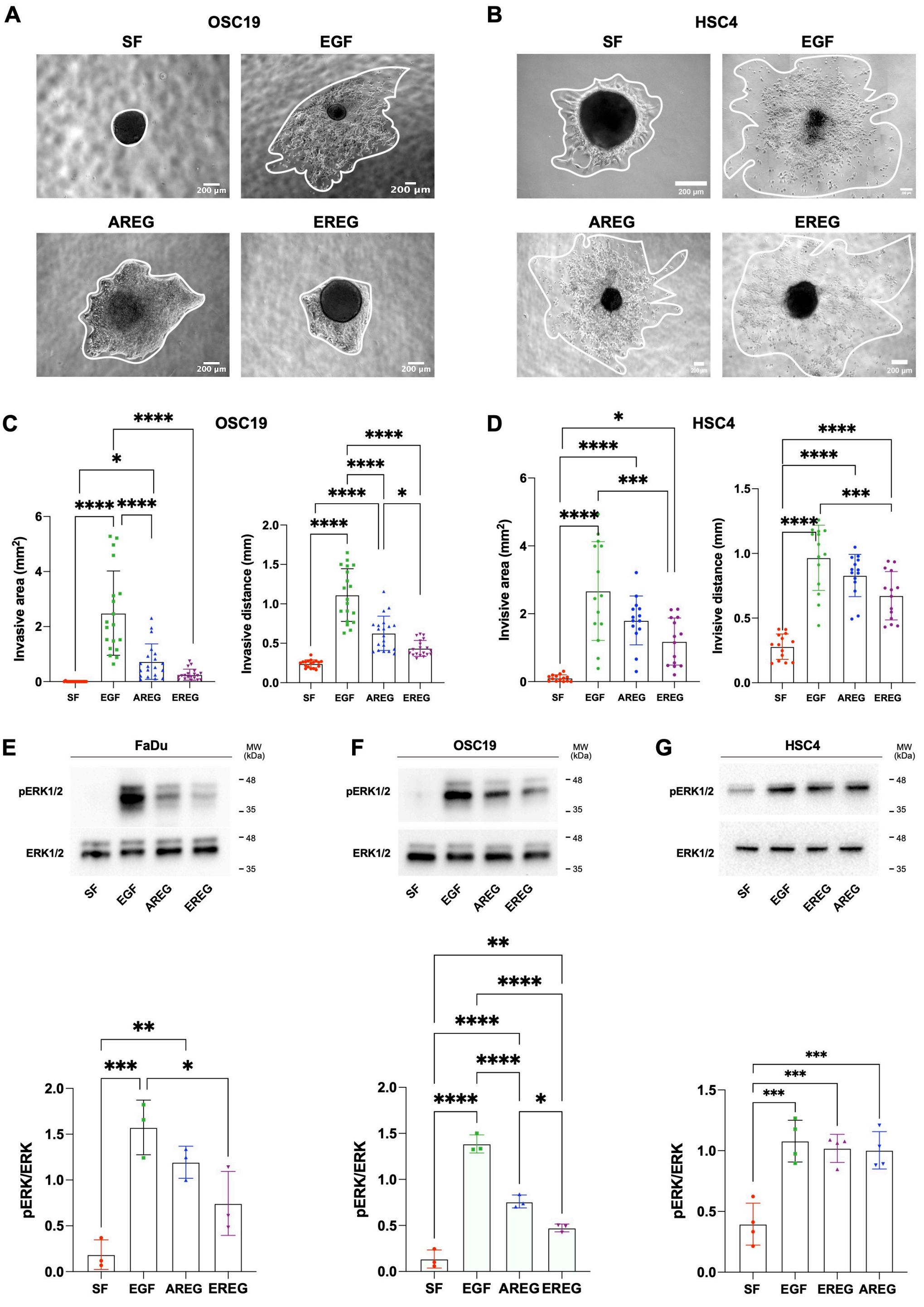


**Suppl. Figure 7: Effects of AREG and EREG on local invasion**

(**A-B**) OSC19 and HSC4 cell spheroids were embedded in type I collagen and treated with EGF (9 nM), AREG (1,000 ng/mL), or EREG (80 ng/mL). Shown are representative pictures from n = 3 independent experiments with 5-10 spheroids per treatment and experiment. (**C-D**) Quantification of invasive area and distance from n = 3 independent experiments with 5-10 spheroids each per treatment. Shown are mean invasive areas and distances with SD for OSC19 (C) and HSC4 cells (D). (**E-G**) FaDu (E), OSC19 (F), and HSC4 cells (G) were treated in monolayer cultures with EGF (9 nM), AREG (1,000 ng/mL), or

EREG (80 ng/mL) under serum-free conditions (10 min). Phosphorylated and total levels of ERK1/2 were visualized by immunoblotting with specific antibodies. Shown are representative immunoblots (top) and quantifications as mean with SD from n = 3 independent experiments (bottom). * p-value < 0.05, **** < 0.0001.

**A**

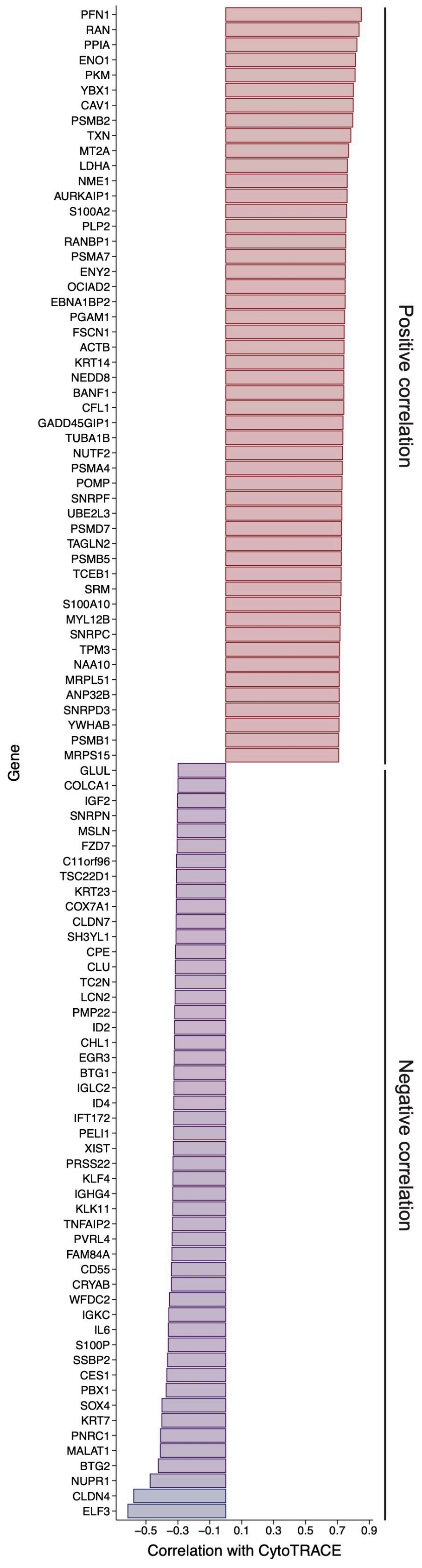

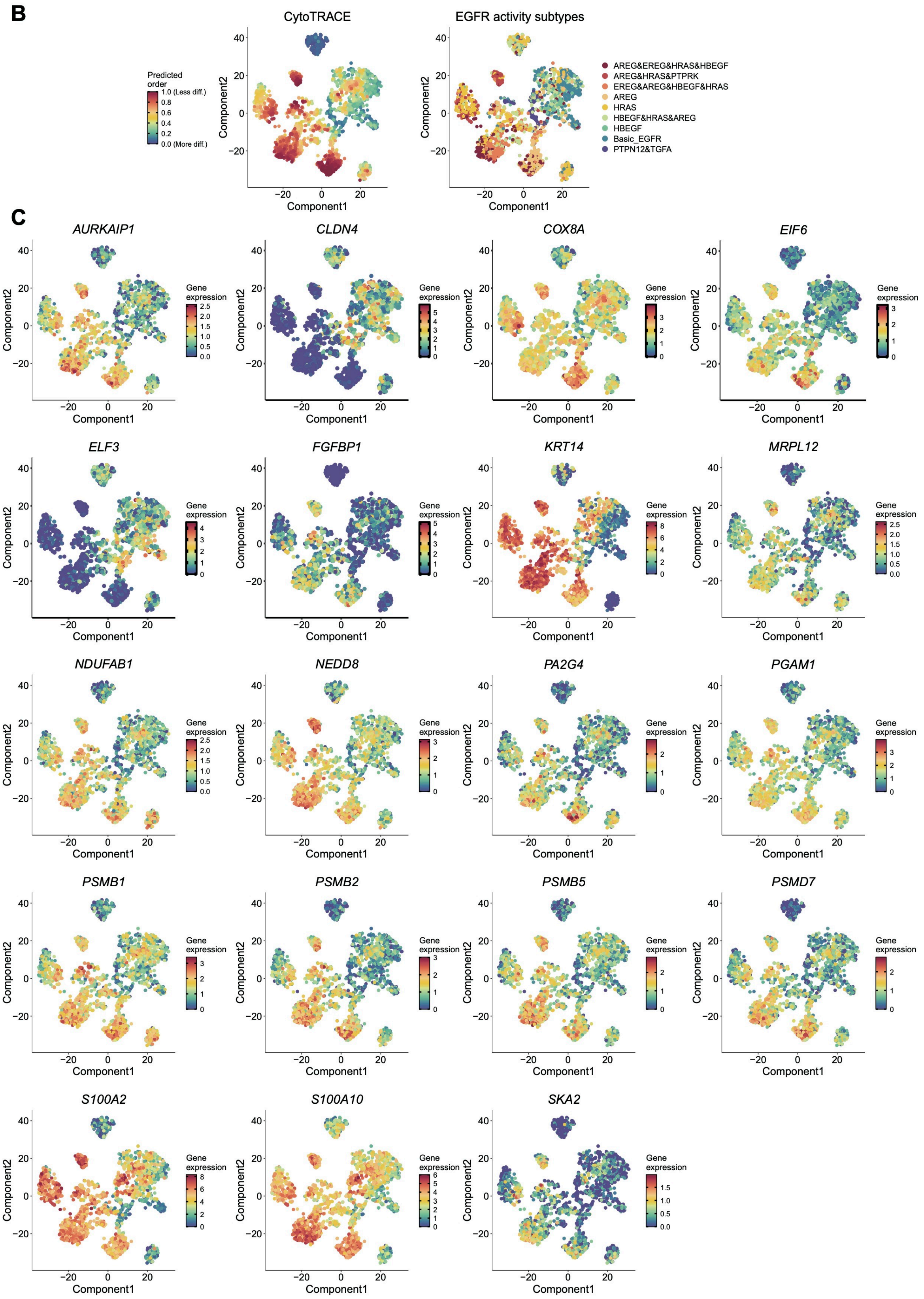


**Suppl. Figure 8: Expression of CytoTRACE differentiation score-associated genes in GSE181919** (**A**) Shown are the top n = 50 genes positively and the top n = 50 genes negatively correlated with the CytoTRACE score in malignant epithelial cells of GSE181919 (Pearson). (**B**) UMAP representation of CytoTRACE differentiation scores (left) and EGFR-activity subtypes (right) in malignant epithelial cells of GSE181919. Shown is the predicted order from more to less differentiated and all EGFR-activity subtypes by color-coding. (**C**) UMAP representation of n = 19 genes associated with high CytoTRACE scores from GSE181919 that are significantly correlated to OS in HPV-neg. HNSCC-TCGA samples.

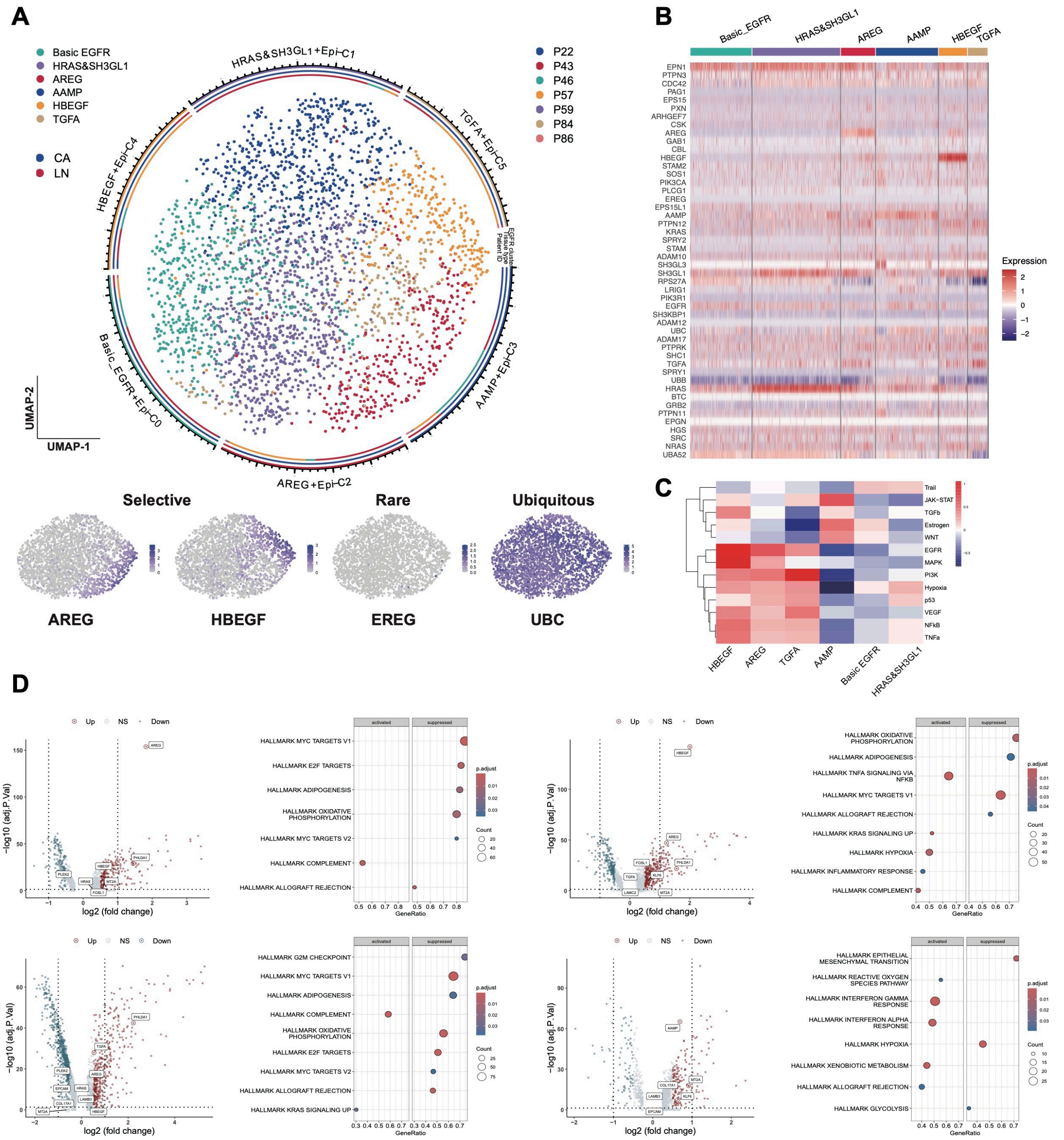


## Suppl. Figure 9: Identification of EGFR-activity subtypes in HPV-pos. HNSCC

(**A**) EGFR-activity subtypes identified by non-negative matrix factorization (NMF) from HPV-pos. malignant HNSCC cells (primary tumor and nodal metastases) are depicted in a UMAP with tissue type of origin and patient ID. Examples of selective genes (AREG, HBEGF), rare genes (EREG), and ubiquitous genes (UBC) are depicted in the lower UMAPs. (**B**) Heatmap representation of the gene expression of “REACTOME_SIGNALING_BY_EGFR” in EGFR-activity subtypes. (**C**) Heatmap of intensity scores of n = 13 PROGENy signaling pathways for all EGFR-activity subtypes of HPV-pos. malignant cells. (**D**) Volcano plots with log2FC and p-value of DEGs from “AREG” versus “Basic EGFR” subtypes and “HBEGF” versus “Basic EGFR” subtypes are shown. Gene set variation analysis (GSVA) of “AREG” versus ”Basic EGFR” and “HBEGF” versus ”Basic EGFR” are presented (right panels). Shown are significantly activated and suppressed human cancer hallmarks (MSigDB) with gene counts and adjusted p-values.

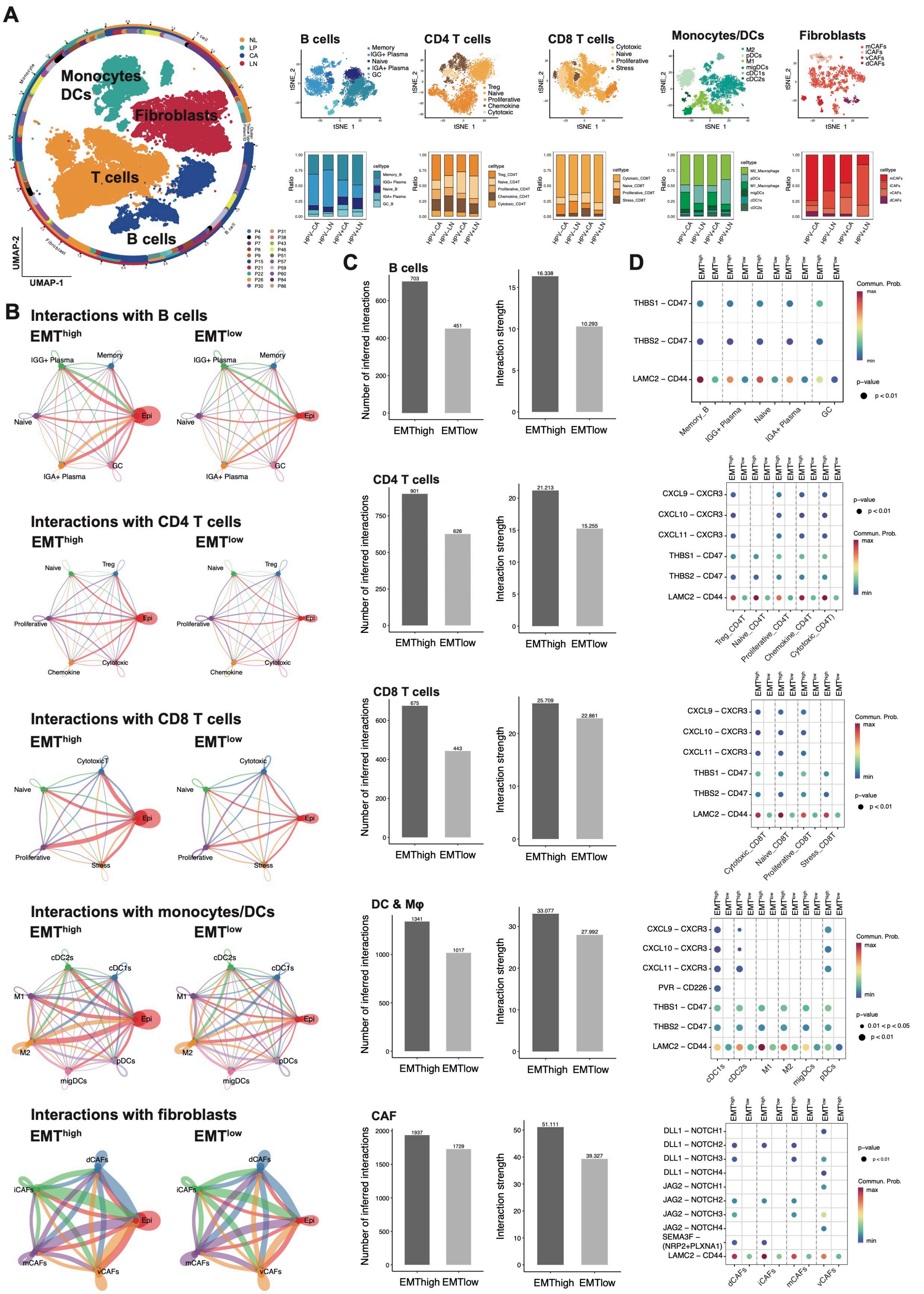

**Suppl. Figure 10: EMT$^{high}$-associated EGFR-activity subtypes interactions with non-malignant cells**

(**A**) B cells, CD4 and CD8 T cells, monocytes, dendritic cells, and fibroblasts were annotated in GSE181919. Shown are UMAP representations of all immune and stromal cells (left), and single UMAP representations for each cell subtype with stacked bar graphs of proportions of B cells, CD4 and CD8 T cells, monocytes, dendritic cells, and fibroblast subtypes, as indicated (right). (**B**) Interactions of

malignant cells with highest EMT scores (EMT$^{high}$, “AREG&HRAS&PTPRK” subtype) and lowest EMT scores (EMT$^{low}$, “Basic EGFR” subtype) with B cells, CD4 and CD8 T cells, monocytes, dendritic cells, and fibroblast subtypes are shown. (**C**) Numbers and interaction strength of EMT$^{high}$ and EMT$^{low}$ malignant cells with B cells, CD4 and CD8 T cells, monocytes, dendritic cells, and fibroblasts are shown as bar graphs. (**D**) Selective ligand-receptor interactions of EMT$^{high}$ and EMT$^{low}$ malignant cells with B cells, CD4 and CD8 T cells, monocytes, dendritic cells, and fibroblasts are shown as dot plots, with communication probabilities, p-values, and LAMC-CD44 interactions as reference. First gene = malignant cells, second gene = non-malignant cells.

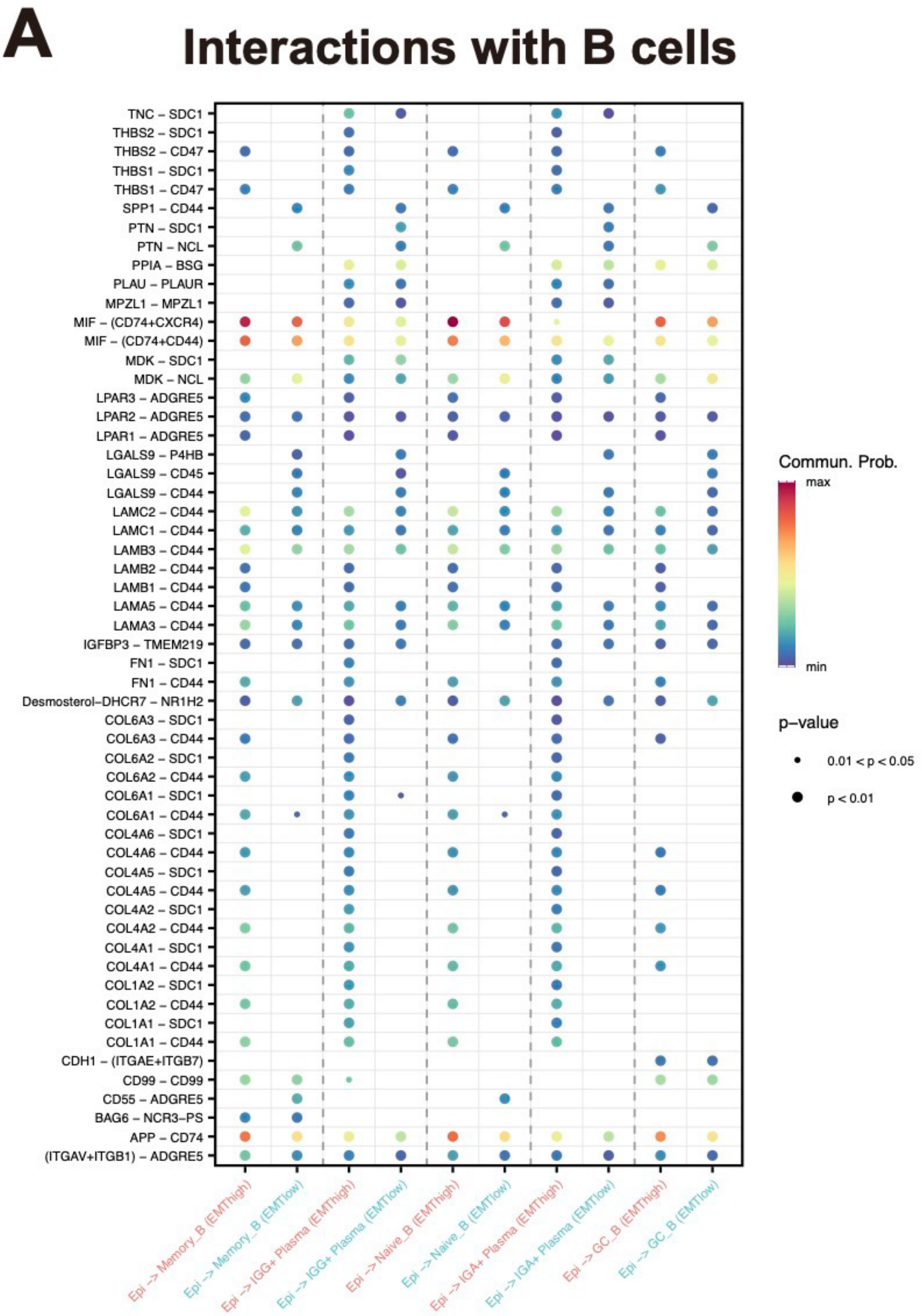

B

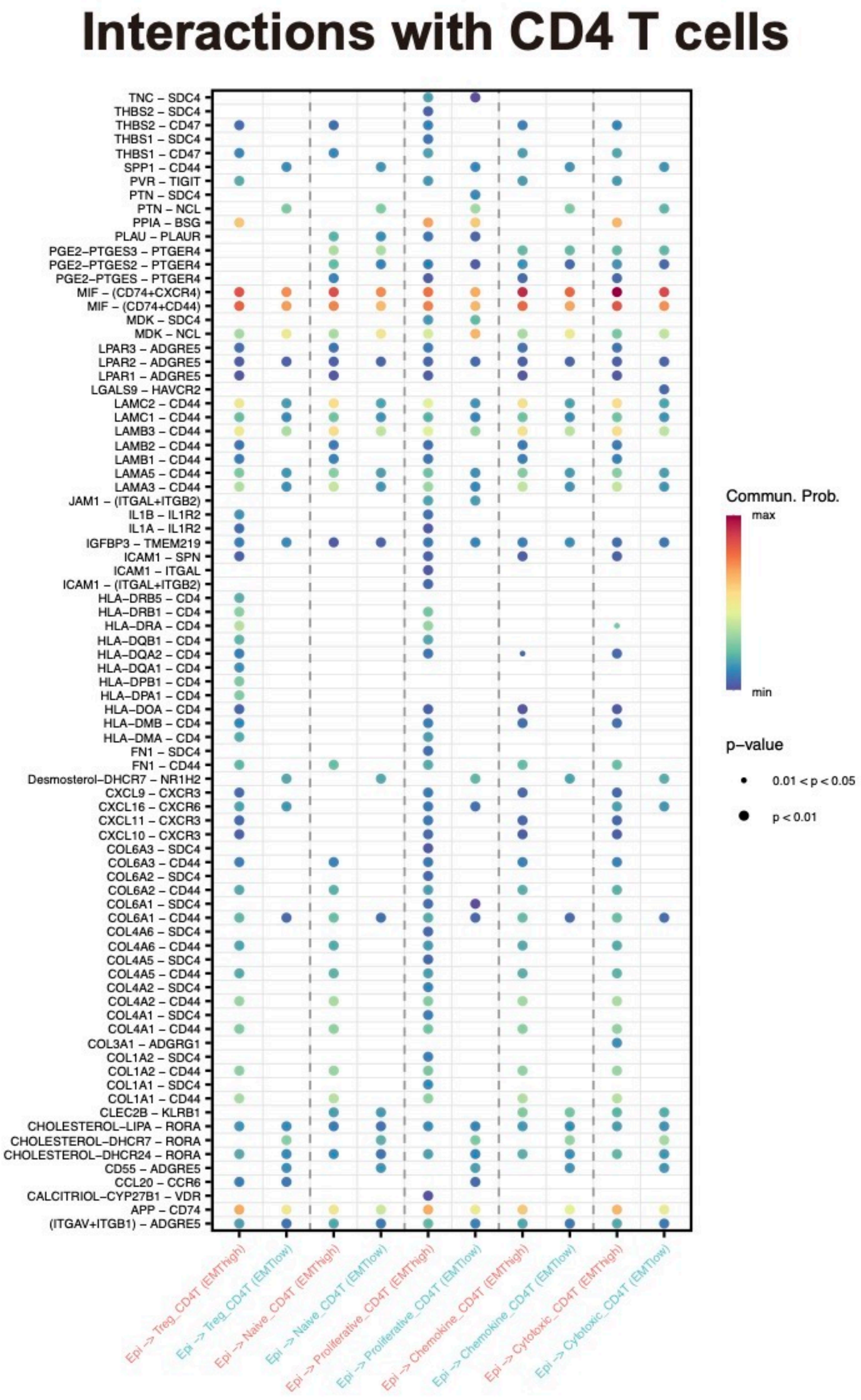
Interactions with CD4 T cells
TNC – SDC4
THBS2 – SDC4
THBS2 – CD47
THBS1 – SDC4
THBS1 – CD47
SPP1 – CD44
PVR – TIGIT
PTN – SDC4
PTN – NCL
PPIA – BSG
PLAU – PLAUR
PGE2–PTGES3 – PTGER4
PGE2–PTGES2 – PTGER4
PGE2–PTGES – PTGER4
MIF – (CD74+CXCR4)
MIF – (CD74+CD44)
MDK – SDC4
MDK – NCL
LPAR3 – ADGRE5
LPAR2 – ADGRE5
LPAR1 – ADGRE5
LGALS9 – HAVCR2
LAMC2 – CD44
LAMC1 – CD44
LAMB3 – CD44
LAMB2 – CD44
LAMB1 – CD44
LAMA5 – CD44
LAMA3 – CD44
JAM1 – (ITGAL+ITGB2)
IL1B – IL1R2
IL1A – IL1R2
IGFBP3 – TMEM219
ICAM1 – SPN
ICAM1 – ITGAL
ICAM1 – (ITGAL+ITGB2)
HLA–DRB5 – CD4
HLA–DRB1 – CD4
HLA–DRA – CD4
HLA–DQB1 – CD4
HLA–DQA2 – CD4
HLA–DQA1 – CD4
HLA–DPB1 – CD4
HLA–DPA1 – CD4
HLA–DOA – CD4
HLA–DMB – CD4
HLA–DMA – CD4
FN1 – SDC4
FN1 – CD44
Desmosterol–DHCR7 – NR1H2
CXCL9 – CXCR3
CXCL16 – CXCR6
CXCL11 – CXCR3
CXCL10 – CXCR3
COL6A3 – SDC4
COL6A3 – CD44
COL6A2 – SDC4
COL6A2 – CD44
COL6A1 – SDC4
COL6A1 – CD44
COL4A6 – SDC4
COL4A6 – CD44
COL4A5 – SDC4
COL4A5 – CD44
COL4A2 – SDC4
COL4A2 – CD44
COL4A1 – SDC4
COL4A1 – CD44
COL3A1 – ADGRG1
COL1A2 – SDC4
COL1A2 – CD44
COL1A1 – SDC4
COL1A1 – CD44
CLEC2B – KLRB1
CHOLESTEROL–LIPA – RORA
CHOLESTEROL–DHCR7 – RORA
CHOLESTEROL–DHCR24 – RORA
CD55 – ADGRE5
CCL20 – CCR6
CALCITRIOL–CYP27B1 – VDR
APP – CD74
(ITGAV+ITGB1) – ADGRE5
Epi -> Treg_CD4T (EMThigh)
Epi -> Treg_CD4T (EMTlow)
Epi -> Naive_CD4T (EMThigh)
Epi -> Naive_CD4T (EMTlow)
Epi -> Proliferative_CD4T (EMThigh)
Epi -> Proliferative_CD4T (EMTlow)
Epi -> Chemokine_CD4T (EMThigh)
Epi -> Chemokine_CD4T (EMTlow)
Epi -> Cytotoxic_CD4T (EMThigh)
Epi -> Cytotoxic_CD4T (EMTlow)
Commun. Prob.
max
min
p–value
0.01 < p < 0.05
p < 0.01

# C Interactions with CD8 T cells

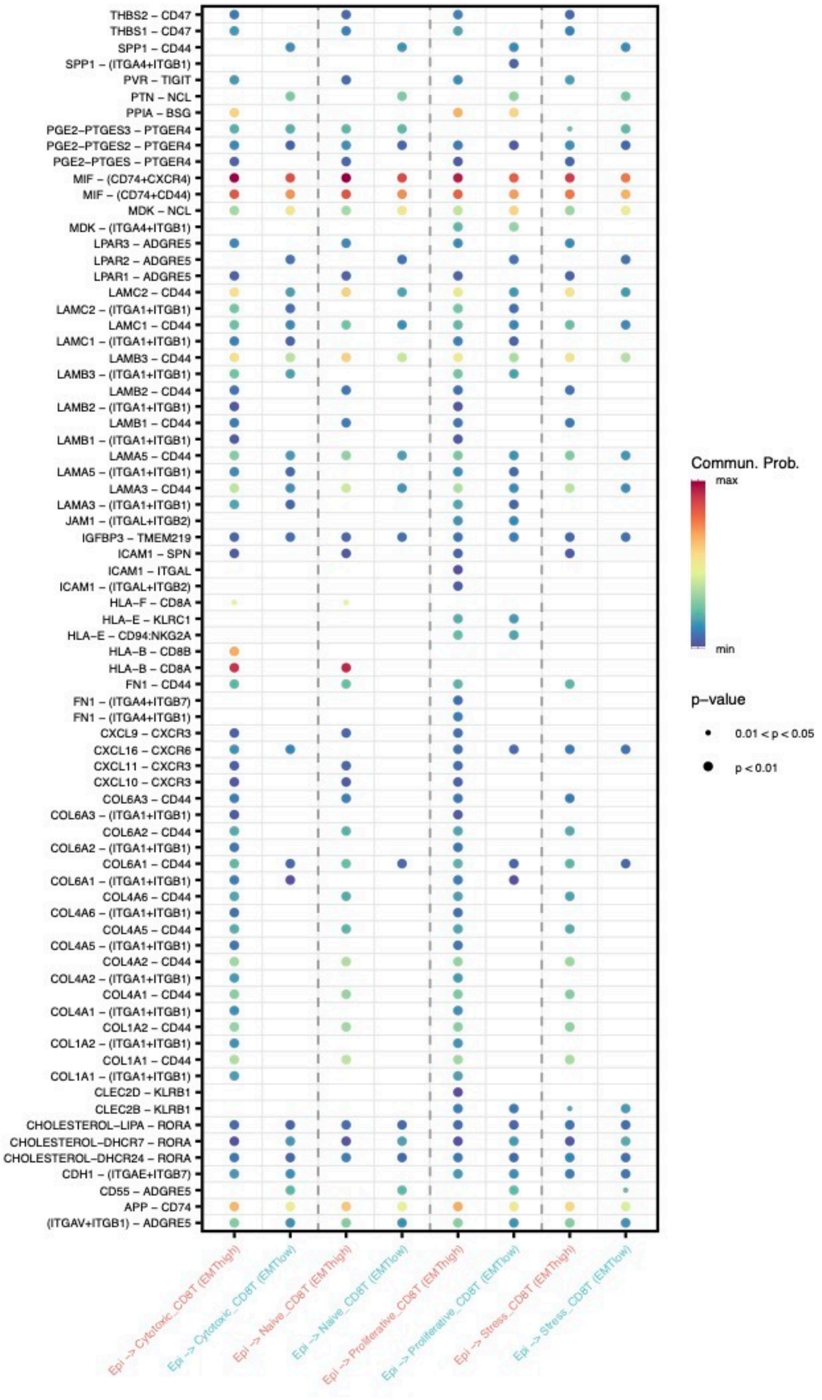

# D Interactions with monocytes/DCs

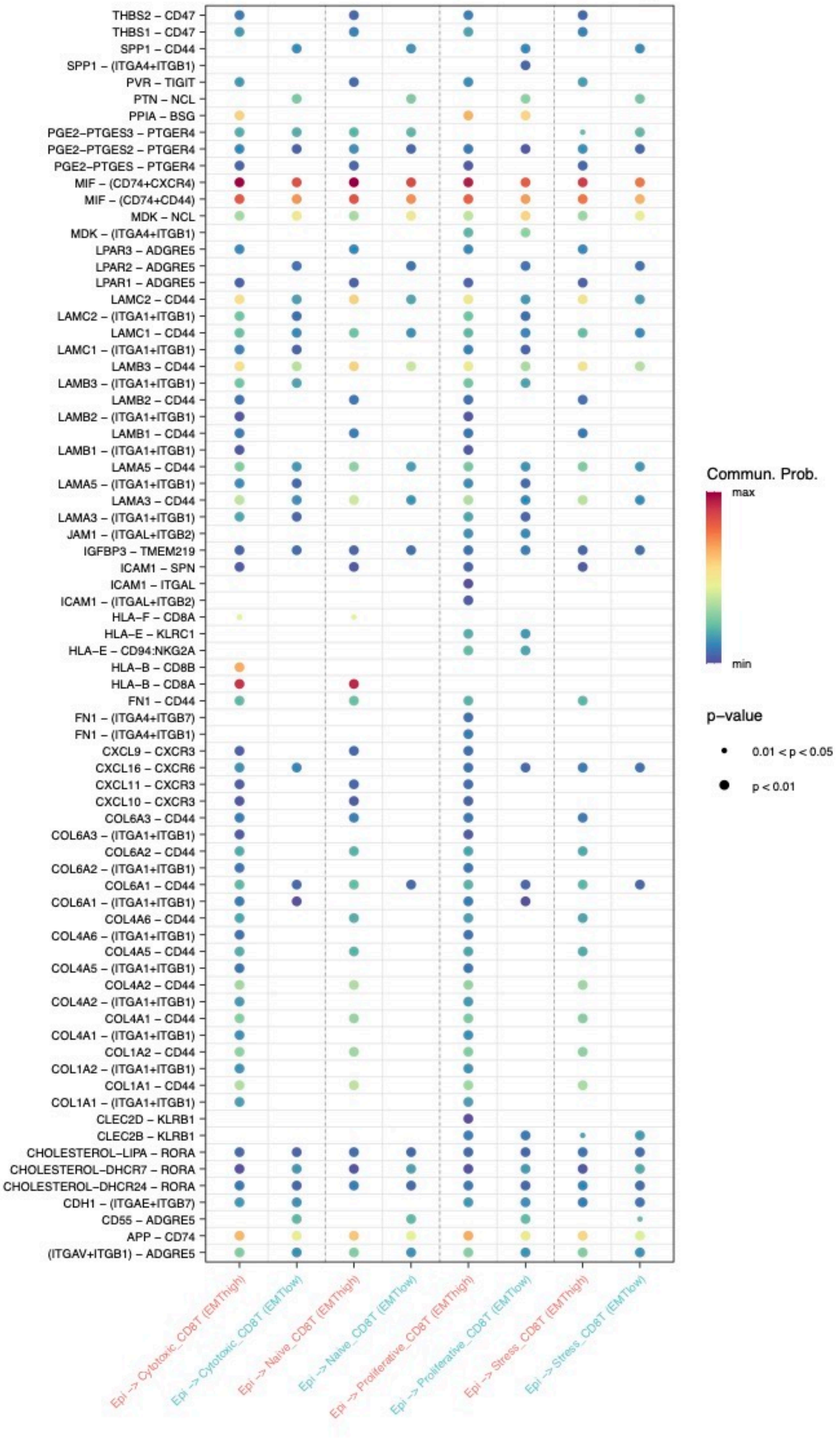

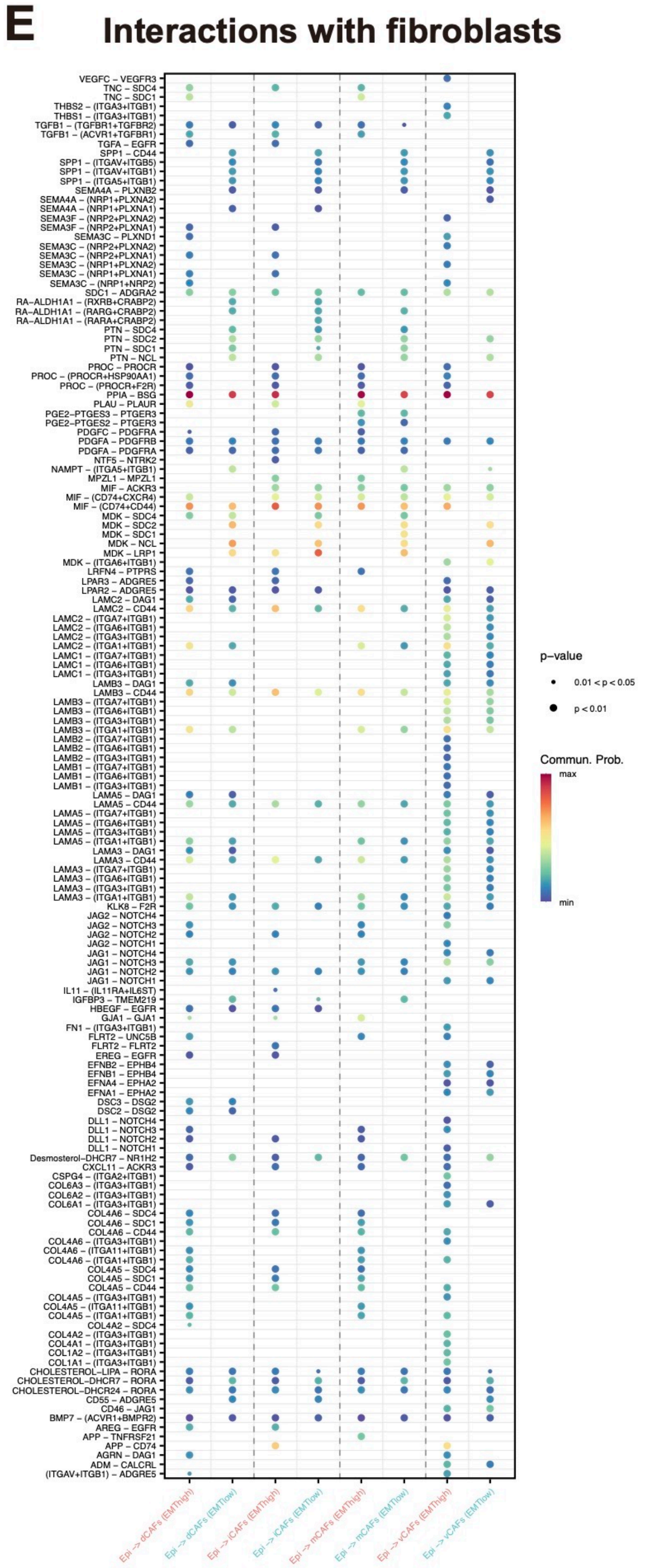


**Suppl. Figure 11: Ligand-receptor interactions of EMT$^{high}$ and EMT$^{low}$ malignant cells with non-malignant cells**

All ligand-receptor interactions of EMT$^{high}$ and EMT$^{low}$ malignant cells (GSE181919) with B cells (**A**), CD4 and CD8 T cells (**B, C**), monocytes and dendritic cells (**D**), and fibroblasts (**E**) are shown as dot plots, with communication probabilities, and p-values. First gene = malignant cells, second gene = non-malignant cells.

A

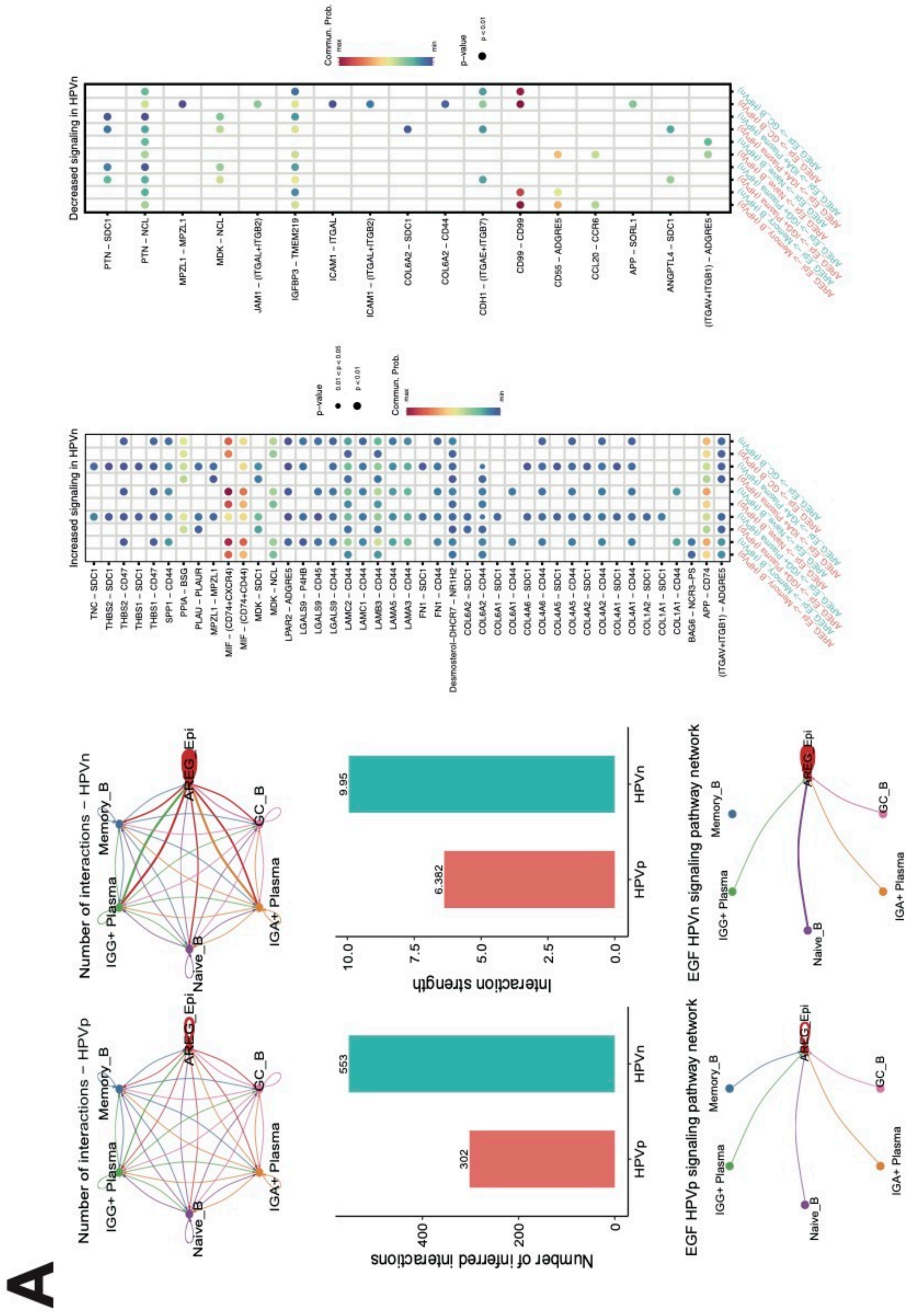
Number of interactions – HPVp
Number of interactions – HPVn
AREG_Epi
Memory_B
GC_B
IGA+ Plasma
Naive_B
IGG+ Plasma
Number of inferred interactions
302
553
HPVp
HPVn
Interaction strength
6.382
9.95
EGF HPVp signaling pathway network
EGF HPVn signaling pathway network
Increased signaling in HPVn
Decreased signaling in HPVn
Commun. Prob.
p-value
p < 0.01
0.01 < p < 0.05

B

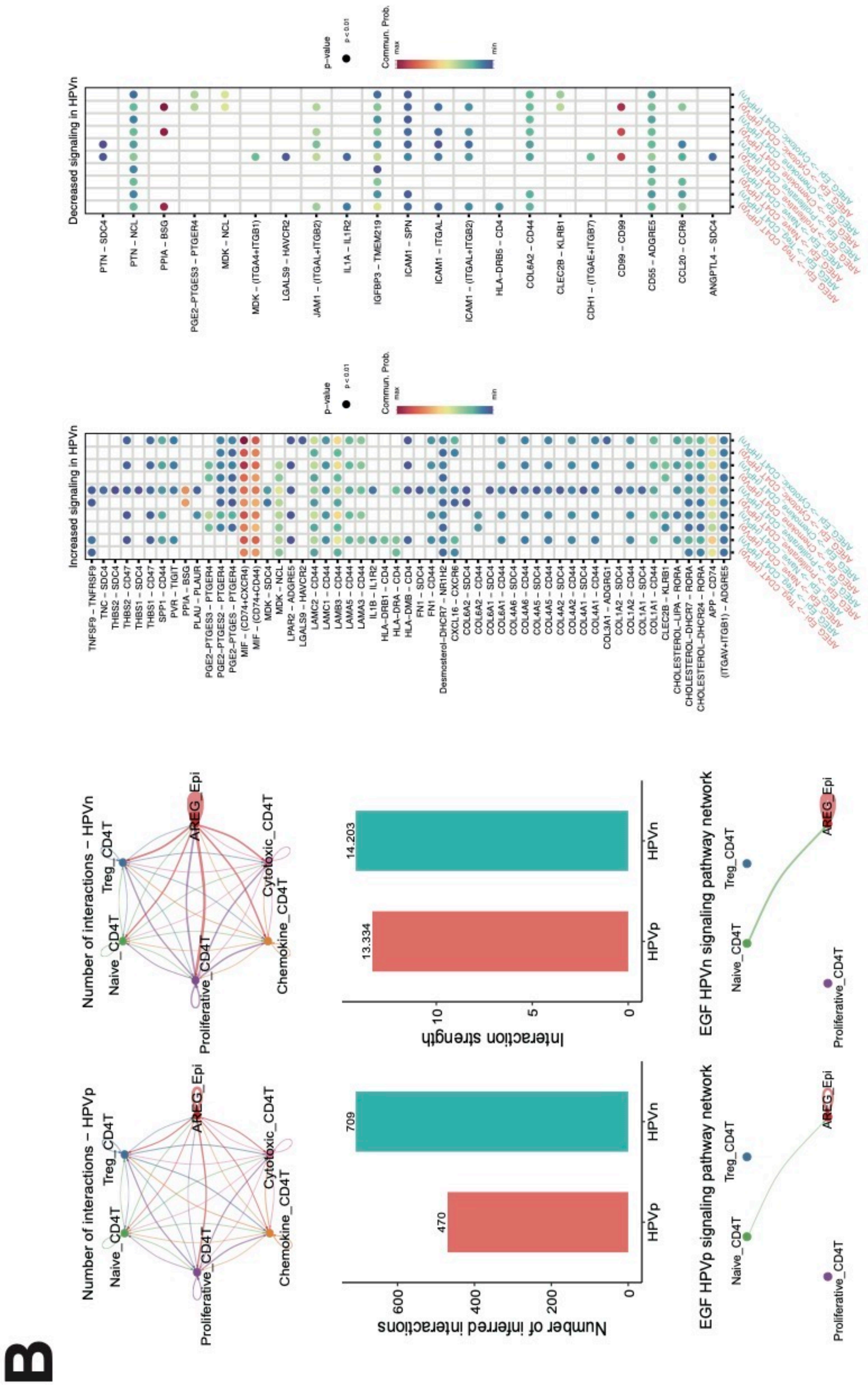
Number of interactions – HPVp
Number of interactions – HPVn
AREG_Epi
Treg_CD4T
Naive_CD4T
Proliferative_CD4T
Chemokine_CD4T
Cytotoxic_CD4T
Number of inferred interactions
470
709
HPVp
HPVn
Interaction strength
13.334
14.203
EGF HPVp signaling pathway network
EGF HPVn signaling pathway network
Increased signaling in HPVn
Decreased signaling in HPVn
p-value
p < 0.01
Commun. Prob.
max
min

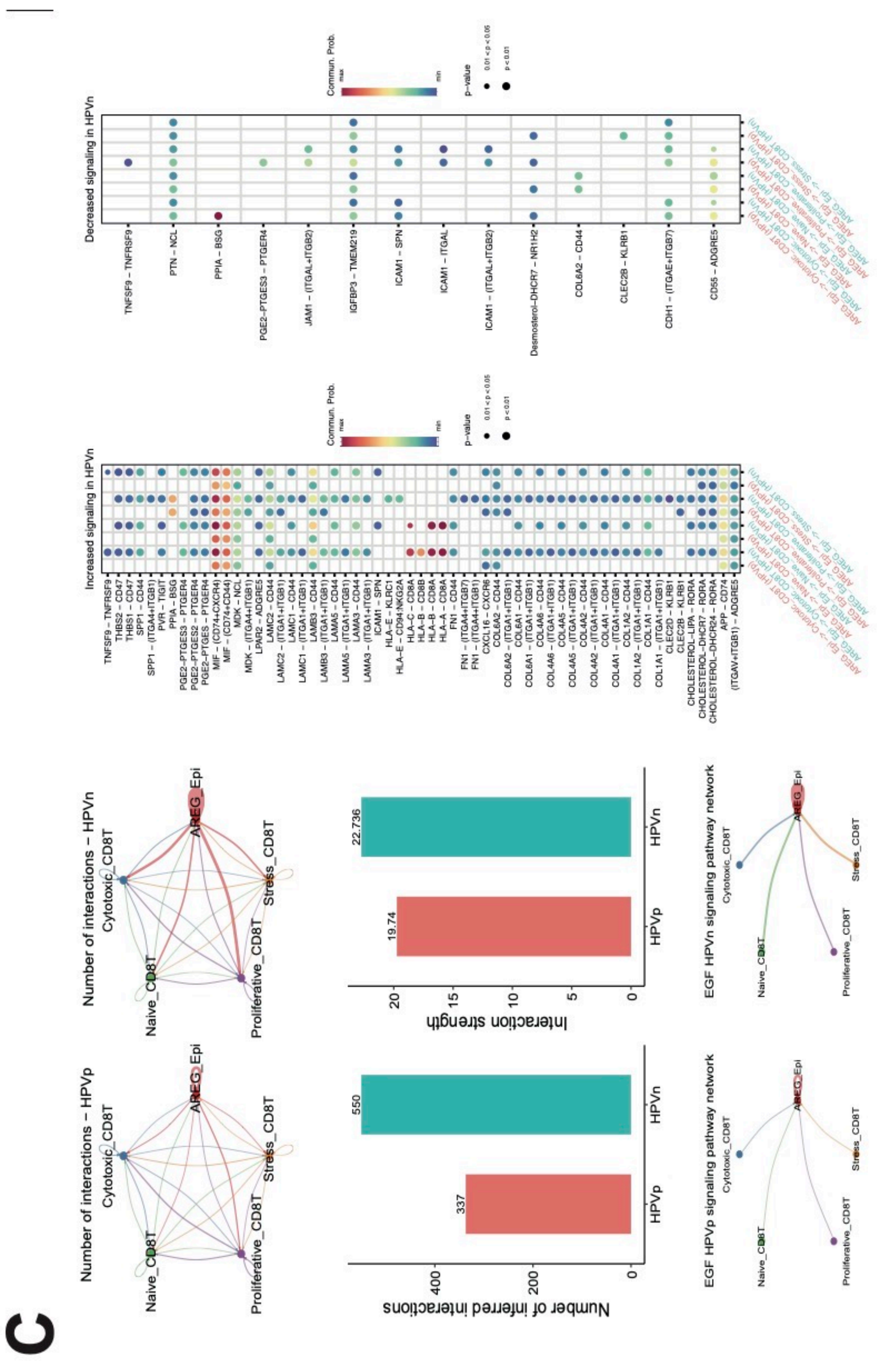
C
Number of interactions – HPVp
Number of interactions – HPVn
AREG_Epi
Cytotoxic_CD8T
Naive_CD8T
Proliferative_CD8T
Stress_CD8T
Number of inferred interactions
337
550
HPVp
HPVn
Interaction strength
19.74
22.736
EGF HPVp signaling pathway network
EGF HPVn signaling pathway network
Increased signaling in HPVn
Decreased signaling in HPVn
Commun. Prob.
max
min
p-value
0.01 < p < 0.05
p < 0.01
TNFSF9 – TNFRSF9
PTN – NCL
PPIA – BSG
PGE2–PTGES3 – PTGER4
JAM1 – (ITGAL+ITGB2)
IGFBP3 – TMEM219
ICAM1 – SPN
ICAM1 – ITGAL
ICAM1 – (ITGAL+ITGB2)
Desmosterol–DHCR7 – NR1H2
COL6A2 – CD44
CLEC2B – KLRB1
CDH1 – (ITGAE+ITGB7)
CD55 – ADGRE5

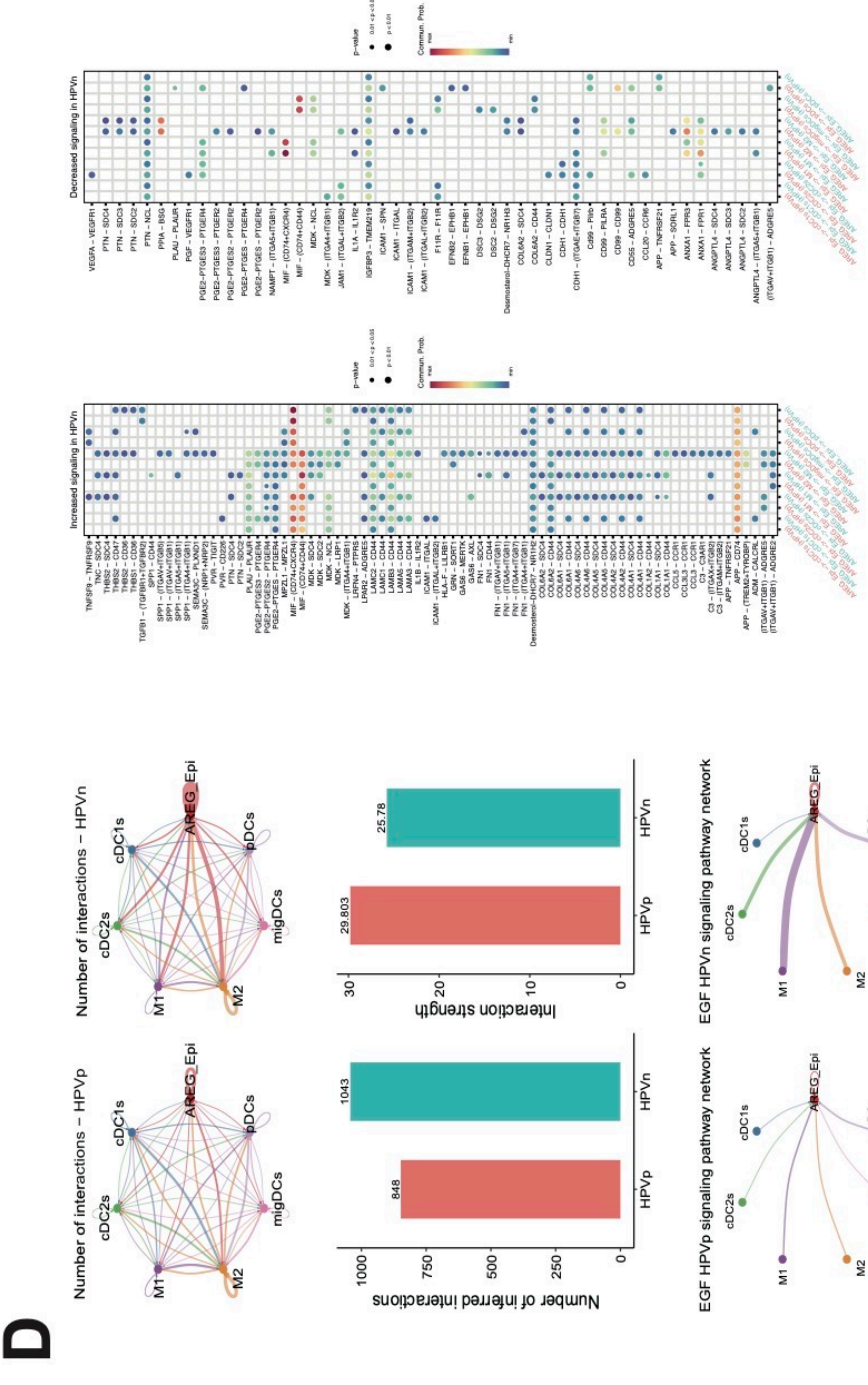
D
Number of interactions – HPVp
Number of interactions – HPVn
Number of inferred interactions
Interaction strength
848
1043
29.803
25.78
HPVp
HPVn
EGF HPVp signaling pathway network
EGF HPVn signaling pathway network
AREG_Epi
cDC1s
cDC2s
pDCs
migDCs
M1
M2
Increased signaling in HPVn
Decreased signaling in HPVn
p-value
Commun. Prob.

E

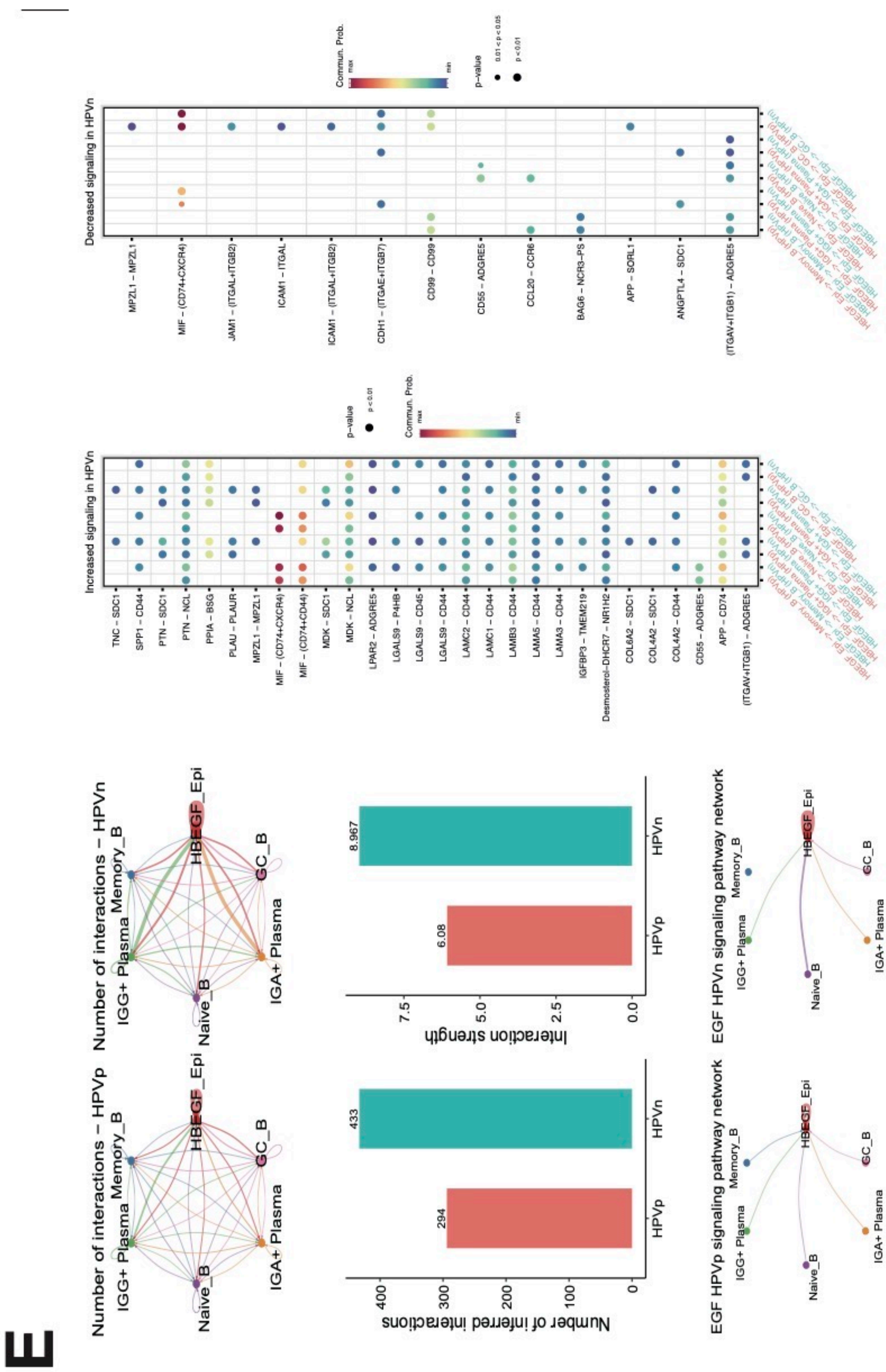

Number of interactions – HPVp
Number of interactions – HPVn
HBEGF_Epi
Memory_B
IGG+ Plasma
Naive_B
IGA+ Plasma
GC_B
Number of inferred interactions
Interaction strength
HPVp
HPVn
294
433
6.08
8.967
EGF HPVp signaling pathway network
EGF HPVn signaling pathway network
Increased signaling in HPVn
Decreased signaling in HPVn
Commun. Prob.
max
min
p-value
p < 0.01
0.01 < p < 0.05

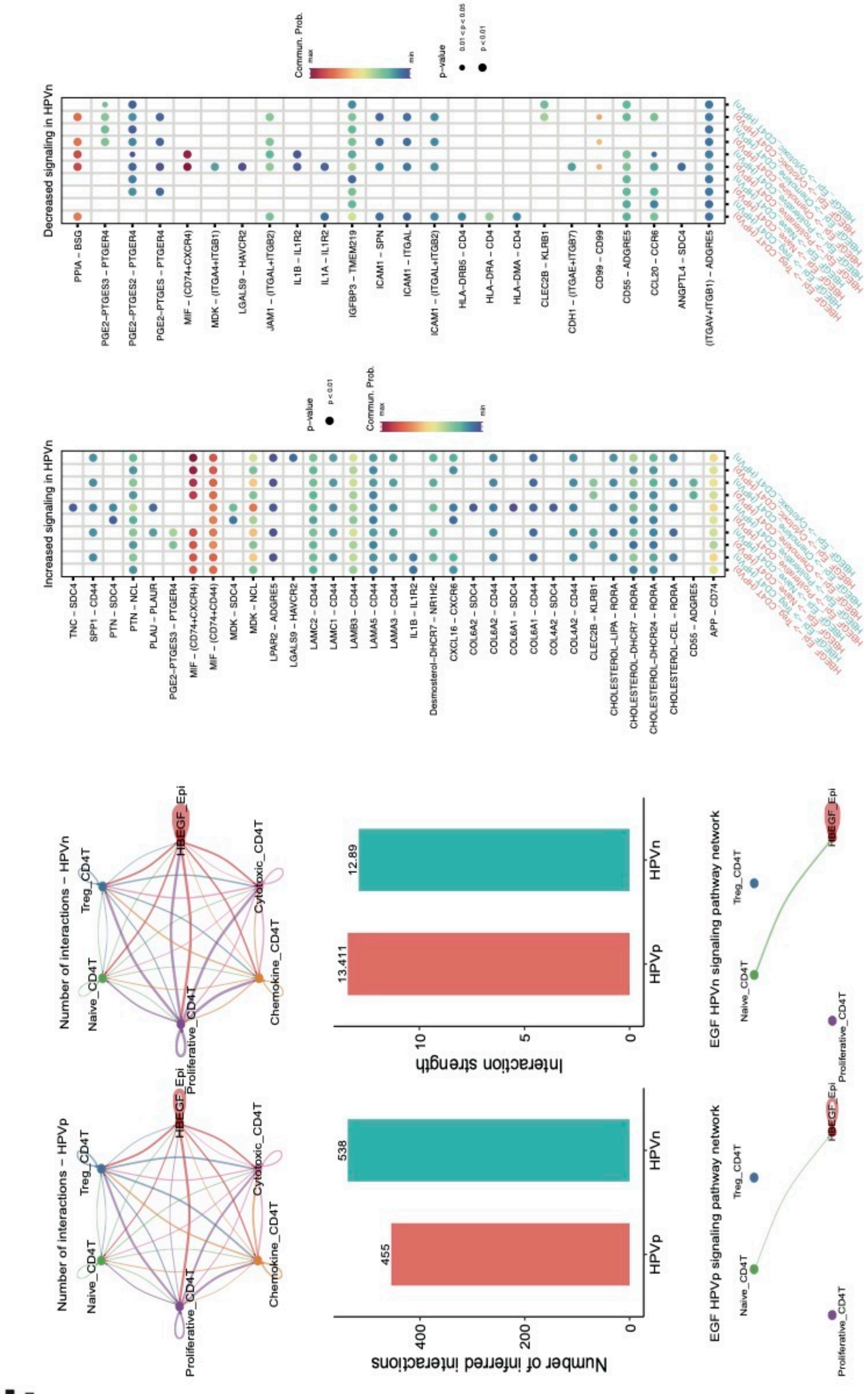

G

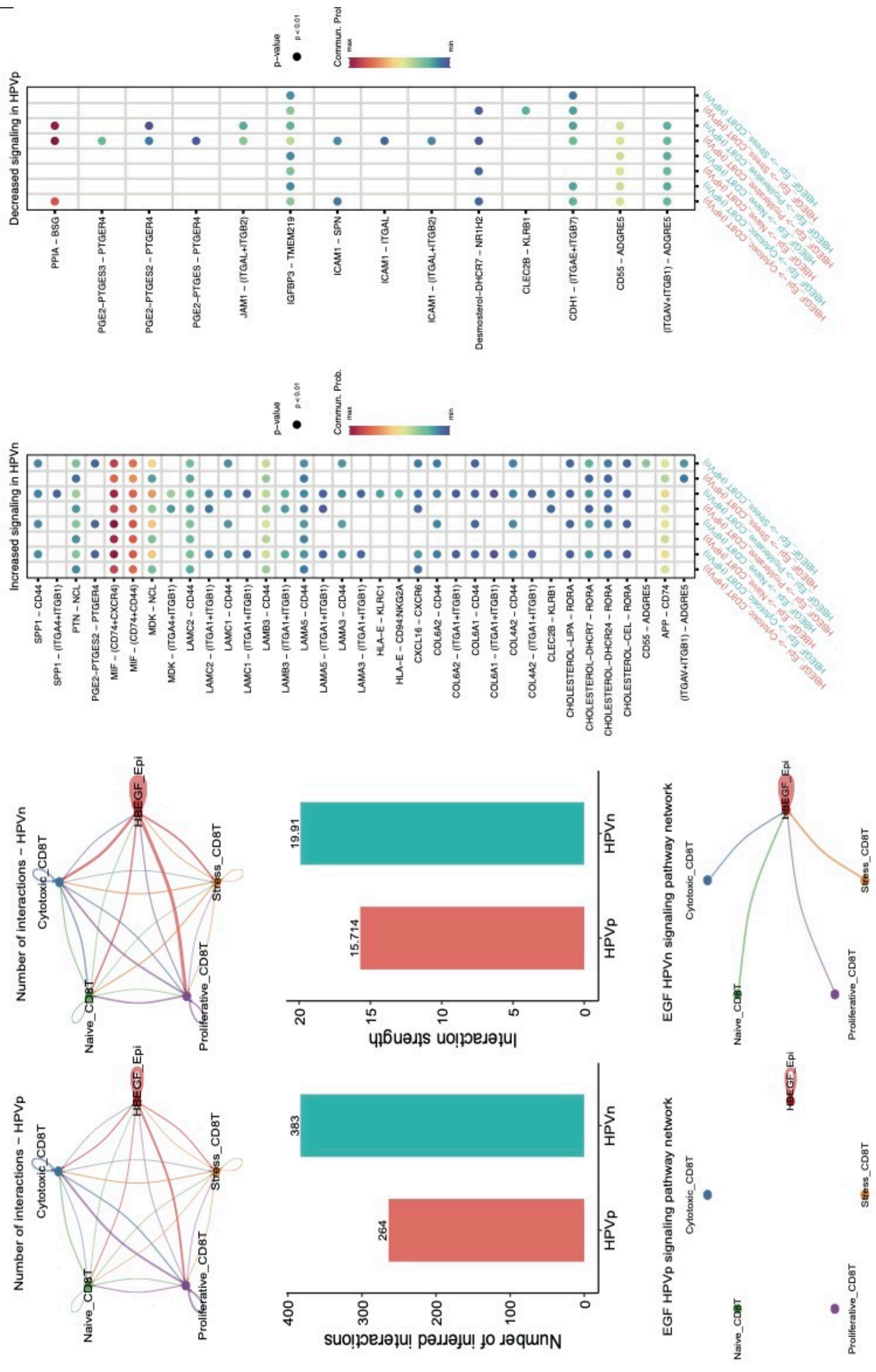

Number of interactions – HPVp
Number of interactions – HPVn
Cytotoxic_CD8T
Naive_CD8T
Proliferative_CD8T
Stress_CD8T
HBEGF_Epi
Number of inferred interactions
264
383
HPVp
HPVn
Interaction strength
15.714
19.91
EGF HPVp signaling pathway network
EGF HPVn signaling pathway network
Increased signaling in HPVn
Decreased signaling in HPVp
p–value
p < 0.01
Commun. Prob.
max
min

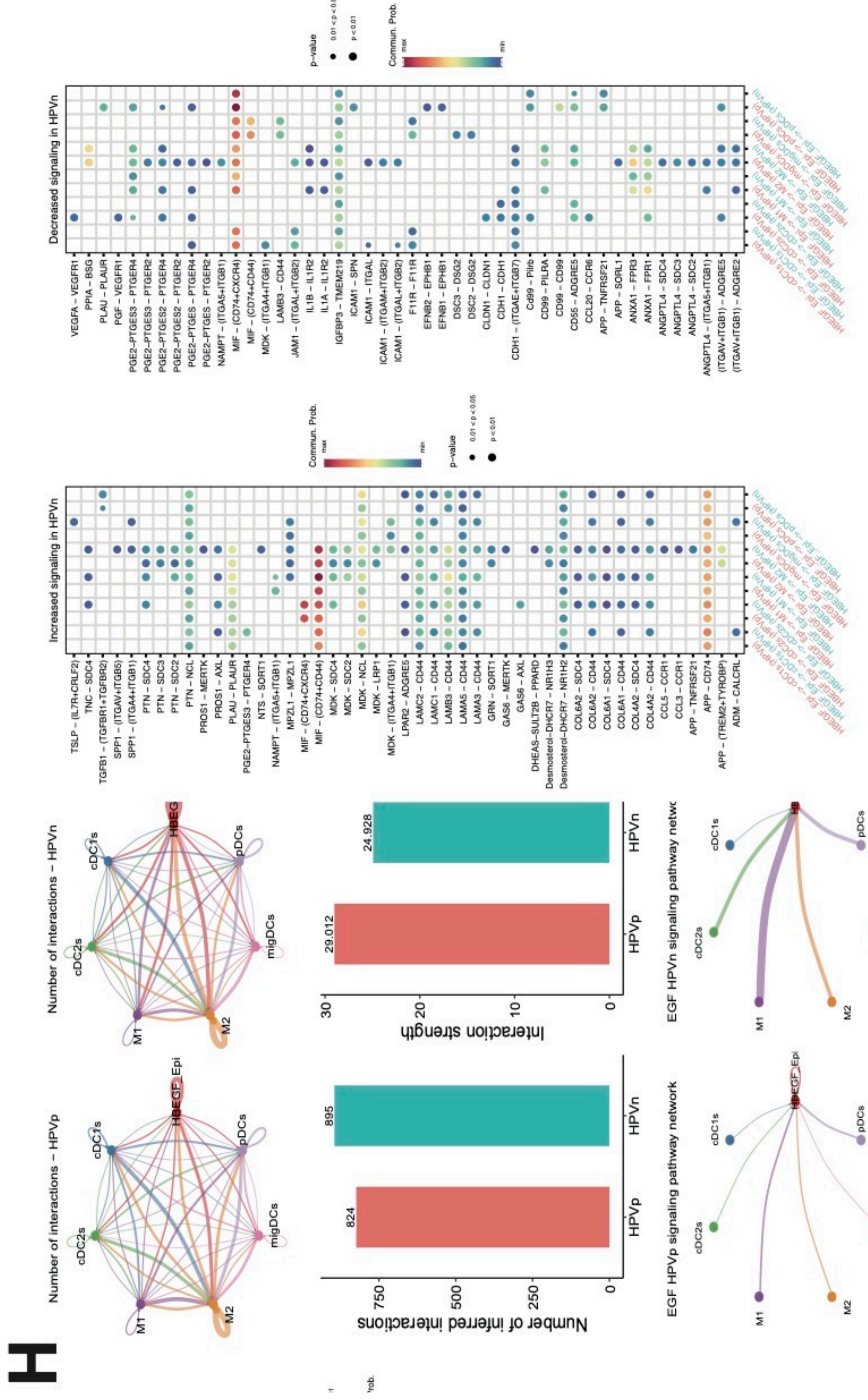
H
Number of interactions – HPVp
Number of interactions – HPVn
Number of inferred interactions
Interaction strength
824
895
29.012
24.928
HPVp
HPVn
EGF HPVp signaling pathway network
Increased signaling in HPVn
Decreased signaling in HPVn
Commun. Prob.
p-value

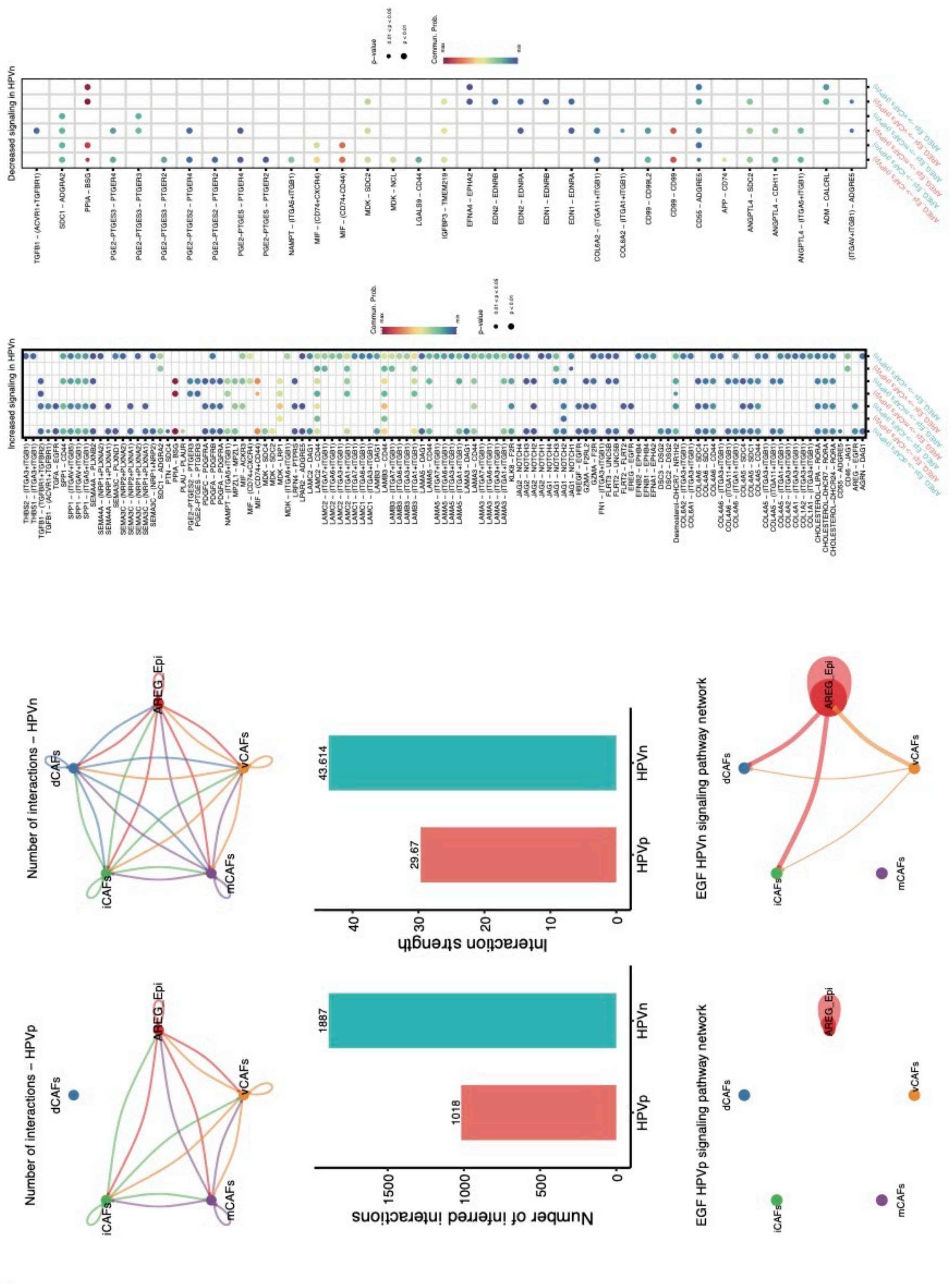
Decreased signaling in HPVn
Increased signaling in HPVn
Commun. Prob.
p-value
Number of interactions – HPVp
Number of interactions – HPVn
AREG_Epi
dCAFs
iCAFs
mCAFs
vCAFs
Number of inferred interactions
Interaction strength
HPVp
HPVn
1018
1887
29.67
43.614
EGF HPVp signaling pathway network
EGF HPVn signaling pathway network

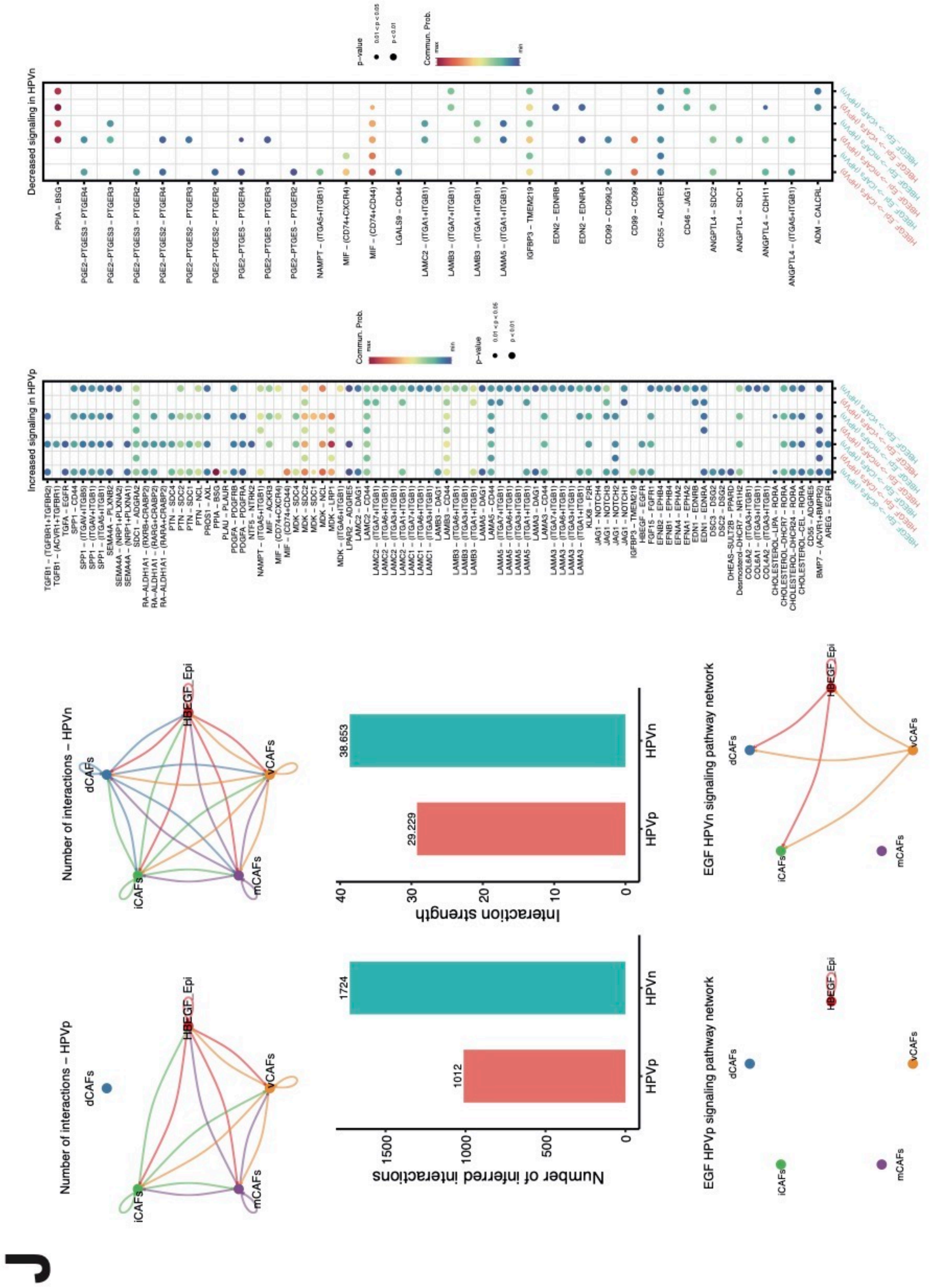


**Suppl. Figure 12: Interactions of HPV-neg. and -pos. malignant cells from GSE181919 with non-malignant cells**

Interactions of malignant HPV-neg. (HPVn) and HPV-pos. (HPVp) cells from GSE181919 with B cells, CD4 and CD8 T cells, monocytes, dendritic cells, and fibroblast subtypes are shown together with numbers and interaction strength and ligand-receptor interaction (communication probalibity and p-value). (**A-D**) Interactions of cells of the “AREG” subtype with immune cells as indicated. (**E-H**) Interactions of cells of the “HBEGF” subtype with immune cells as indicated. (**I**) Interactions of cells of the “AREG” subtype with fibroblast subtypes. (**J**) Interactions of cells of the “HBEGF” subtype with fibroblast subtypes.

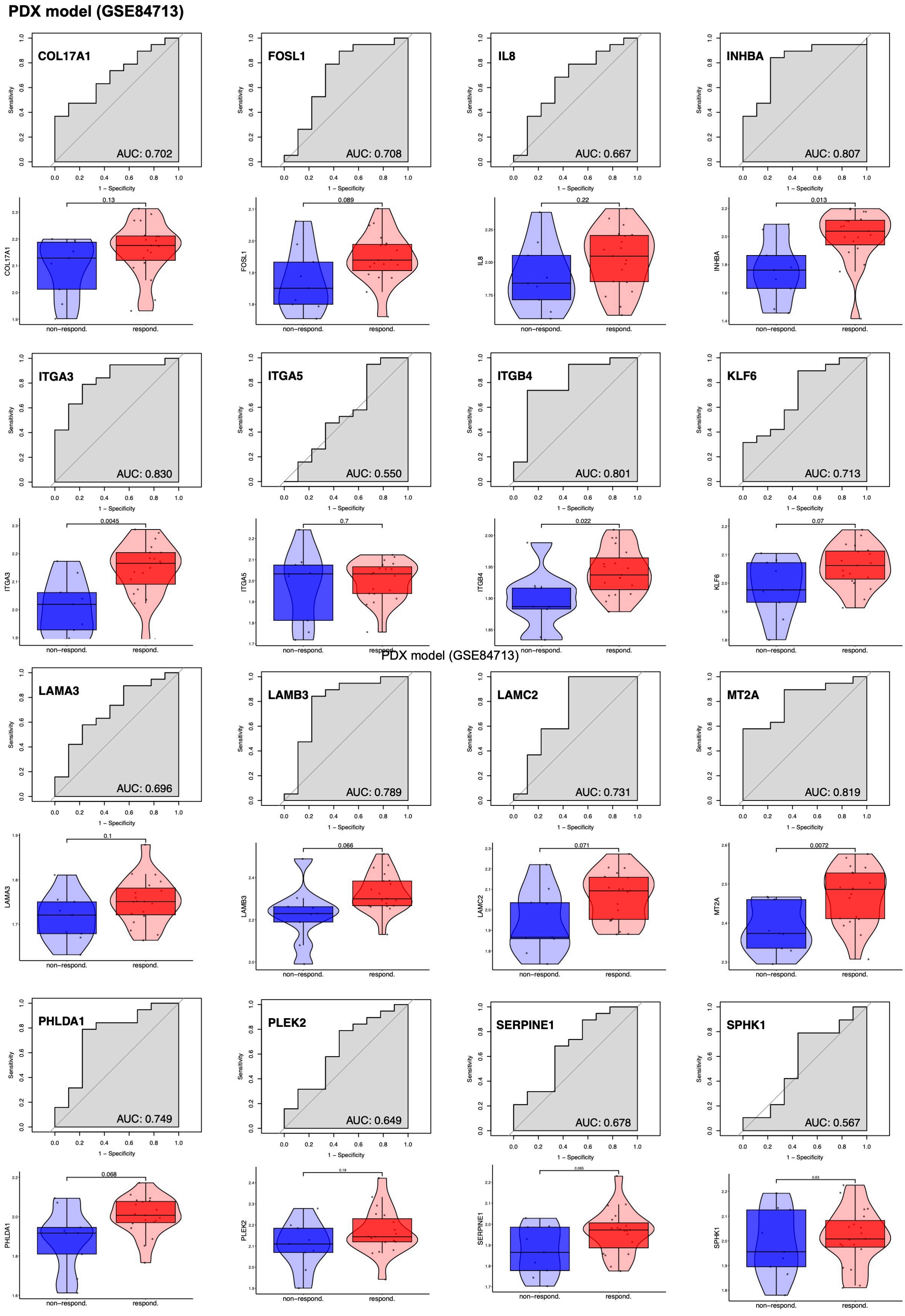


**Suppl. Figure 13: Correlation of fDEGs with Cetuximab response in a PDX-model of treatment**
Area under the curve (AUC) of receiver operating characteristic curves (ROC) for the association of the indicated genes with response to Cetuximab treatment in GSE84713 are shown in the upper graphs. Boxplot-whisker graphs are shown for the expression of the indicated gene with non-responder (blue) and responder (red) samples (displayed are: minimum, 1st quartile, median, 3rd quartile, maximum).

**A** **R/M-HNSCC (GSE65021)**

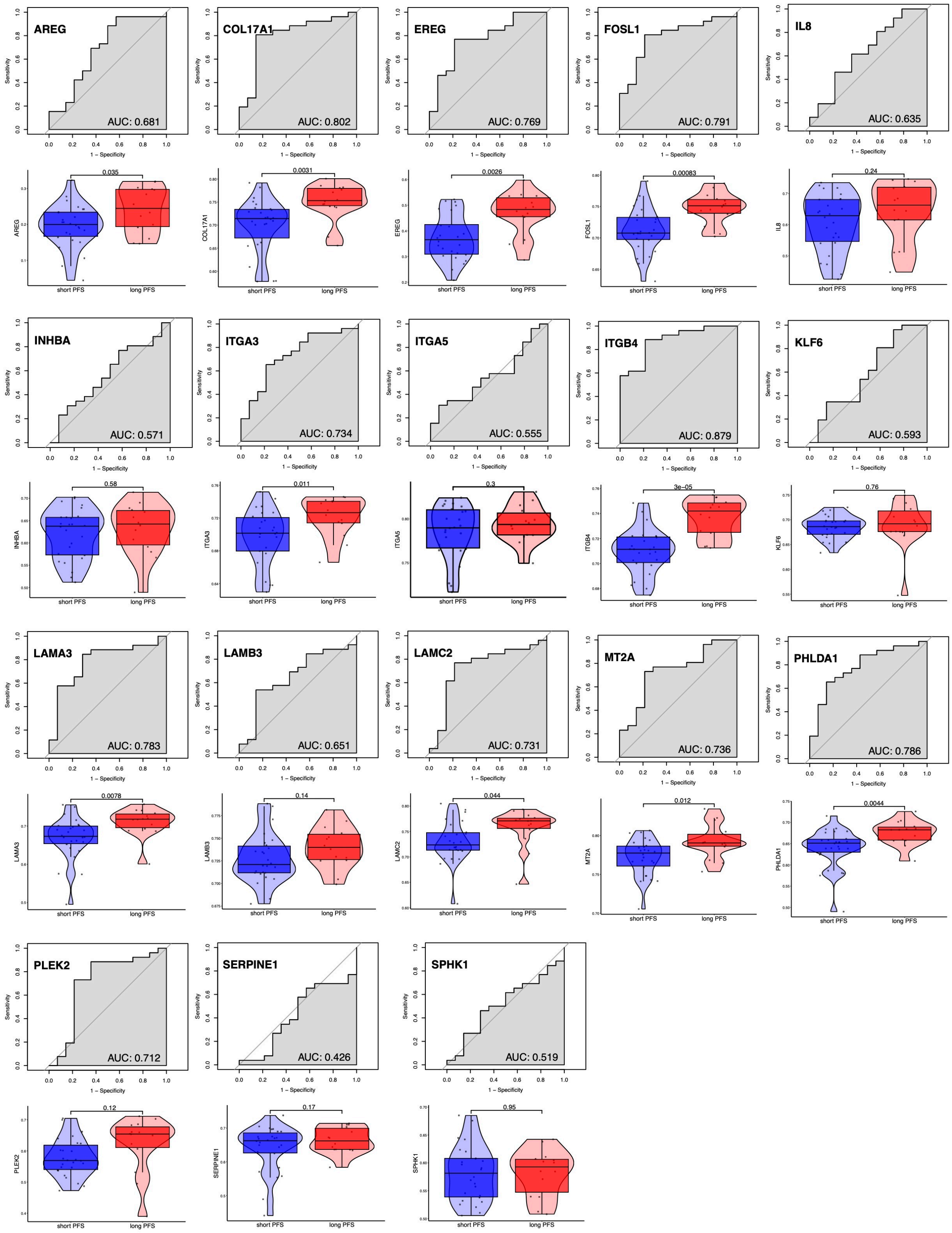

## B R/M-HNSCC Cetuximab treatment (odds of short PFS)

| Variable | | N | Odds ratio | p |
|---|---|---|---|---|
| Age | <60 | 15 | Reference | |
| | >=60 | 25 | 0.30 (0.03, 2.14) | 0.241 |
| Gender | Male | 31 | Reference | |
| | Female | 9 | 1.89 (0.15, 34.04) | 0.632 |
| Stage | Stage1 | 3 | Reference | |
| | Stage2 | 8 | 0.86 (0.02, 32.95) | 0.930 |
| | Stage3 | 9 | 0.03 (0.00, 1.66) | 0.100 |
| | Stage4 | 20 | 0.32 (0.01, 10.12) | 0.495 |
| Grade | Grade1 | 2 | Reference | |
| | Grade2 | 20 | 1.44 (0.02, 111.65) | 0.860 |
| | Grade3 | 18 | 2.48 (0.03, 222.14) | 0.671 |
| Radiotherapy | No | 6 | Reference | |
| | Yes | 34 | 0.34 (0.02, 4.91) | 0.438 |
| COL17A1 | high | 20 | Reference | |
| | low | 20 | 34.68 (4.35, 856.04) | 0.005 |

| Variable | | N | Odds ratio | p |
|---|---|---|---|---|
| Age | <60 | 15 | Reference | |
| | >=60 | 25 | 0.10 (0.00, 0.92) | 0.082 |
| Gender | Male | 31 | Reference | |
| | Female | 9 | 1.36 (0.09, 27.44) | 0.823 |
| Stage | Stage1 | 3 | Reference | |
| | Stage2 | 8 | 0.77 (0.01, 39.22) | 0.890 |
| | Stage3 | 9 | 0.03 (0.00, 1.74) | 0.105 |
| | Stage4 | 20 | 0.30 (0.01, 11.15) | 0.496 |
| Grade | Grade1 | 2 | Reference | |
| | Grade2 | 20 | 5.81 (0.08, 925.94) | 0.429 |
| | Grade3 | 18 | 21.94 (0.29, 3636.85) | 0.172 |
| Radiotherapy | No | 6 | Reference | |
| | Yes | 34 | 0.31 (0.02, 2.84) | 0.325 |
| FOSL1 | high | 20 | Reference | |
| | low | 20 | 24.55 (3.27, 441.69) | 0.008 |

| Variable | | N | Odds ratio | p |
|---|---|---|---|---|
| Age | <60 | 15 | Reference | |
| | >=60 | 25 | 0.27 (0.04, 1.32) | 0.1 |
| Gender | Male | 31 | Reference | |
| | Female | 9 | 1.23 (0.15, 13.24) | 0.9 |
| Stage | Stage1 | 3 | Reference | |
| | Stage2 | 8 | 1.47 (0.04, 39.20) | 0.8 |
| | Stage3 | 9 | 0.33 (0.01, 7.31) | 0.5 |
| | Stage4 | 20 | 1.52 (0.05, 29.06) | 0.8 |
| Grade | Grade1 | 2 | Reference | |
| | Grade2 | 20 | 2.54 (0.04, 171.87) | 0.6 |
| | Grade3 | 18 | 4.87 (0.08, 297.32) | 0.4 |
| Radiotherapy | No | 6 | Reference | |
| | Yes | 34 | 0.63 (0.05, 5.58) | 0.7 |
| IL8 | high | 20 | Reference | |
| | low | 20 | 2.45 (0.53, 12.77) | 0.3 |

| Variable | | N | Odds ratio | p |
|---|---|---|---|---|
| Age | <60 | 15 | Reference | |
| | >=60 | 25 | 0.31 (0.05, 1.45) | 0.2 |
| Gender | Male | 31 | Reference | |
| | Female | 9 | 1.62 (0.19, 19.74) | 0.7 |
| Stage | Stage1 | 3 | Reference | |
| | Stage2 | 8 | 1.61 (0.05, 38.64) | 0.8 |
| | Stage3 | 9 | 0.20 (0.01, 3.99) | 0.3 |
| | Stage4 | 20 | 1.11 (0.04, 17.21) | 0.9 |
| Grade | Grade1 | 2 | Reference | |
| | Grade2 | 20 | 5.82 (0.08, 419.88) | 0.4 |
| | Grade3 | 18 | 9.96 (0.16, 666.43) | 0.2 |
| Radiotherapy | No | 6 | Reference | |
| | Yes | 34 | 0.40 (0.03, 3.61) | 0.4 |
| INHBA | high | 20 | Reference | |
| | low | 20 | 2.30 (0.50, 12.47) | 0.3 |

| Variable | | N | Odds ratio | p |
|---|---|---|---|---|
| Age | <60 | 15 | Reference | |
| | >=60 | 25 | 0.28 (0.03, 1.66) | 0.19 |
| Gender | Male | 31 | Reference | |
| | Female | 9 | 1.81 (0.20, 21.62) | 0.60 |
| Stage | Stage1 | 3 | Reference | |
| | Stage2 | 8 | 2.85 (0.06, 139.43) | 0.58 |
| | Stage3 | 9 | 0.33 (0.01, 13.02) | 0.56 |
| | Stage4 | 20 | 1.46 (0.04, 46.15) | 0.83 |
| Grade | Grade1 | 2 | Reference | |
| | Grade2 | 20 | 5.37 (0.10, 444.99) | 0.40 |
| | Grade3 | 18 | 8.92 (0.16, 727.31) | 0.28 |
| Radiotherapy | No | 6 | Reference | |
| | Yes | 34 | 0.75 (0.06, 7.79) | 0.81 |
| ITGA3 | high | 20 | Reference | |
| | low | 20 | 8.25 (1.67, 56.50) | 0.02 |

| Variable | | N | Odds ratio | p |
|---|---|---|---|---|
| Age | <60 | 15 | Reference | |
| | >=60 | 25 | 0.32 (0.05, 1.54) | 0.2 |
| Gender | Male | 31 | Reference | |
| | Female | 9 | 1.31 (0.17, 14.42) | 0.8 |
| Stage | Stage1 | 3 | Reference | |
| | Stage2 | 8 | 1.48 (0.04, 39.87) | 0.8 |
| | Stage3 | 9 | 0.23 (0.01, 5.23) | 0.4 |
| | Stage4 | 20 | 1.61 (0.05, 35.73) | 0.8 |
| Grade | Grade1 | 2 | Reference | |
| | Grade2 | 20 | 5.11 (0.08, 332.44) | 0.4 |
| | Grade3 | 18 | 8.39 (0.14, 518.43) | 0.3 |
| Radiotherapy | No | 6 | Reference | |
| | Yes | 34 | 0.54 (0.04, 4.58) | 0.6 |
| ITGA5 | high | 20 | Reference | |
| | low | 20 | 2.26 (0.45, 14.24) | 0.3 |

| Variable | | N | Odds ratio | p |
|---|---|---|---|---|
| Age | <60 | 15 | Reference | |
| | >=60 | 25 | 0.11 (0.01, 0.74) | 0.038 |
| Gender | Male | 31 | Reference | |
| | Female | 9 | 6.34 (0.44, 159.98) | 0.207 |
| Stage | Stage1 | 3 | Reference | |
| | Stage2 | 8 | 1.36 (0.02, 103.08) | 0.885 |
| | Stage3 | 9 | 0.19 (0.00, 10.82) | 0.437 |
| | Stage4 | 20 | 1.86 (0.03, 108.72) | 0.752 |
| Grade | Grade1 | 2 | Reference | |
| | Grade2 | 20 | 262.19 (1.28, 158586.69) | 0.051 |
| | Grade3 | 18 | 533.22 (2.47, 456516.95) | 0.034 |
| Radiotherapy | No | 6 | Reference | |
| | Yes | 34 | 0.40 (0.02, 5.49) | 0.491 |
| ITGB4 | high | 20 | Reference | |
| | low | 20 | 39.22 (4.49, 895.20) | 0.005 |

| Variable | | N | Odds ratio | p |
|---|---|---|---|---|
| Age | <60 | 15 | Reference | |
| | >=60 | 25 | 0.26 (0.04, 1.29) | 0.1 |
| Gender | Male | 31 | Reference | |
| | Female | 9 | 1.41 (0.17, 16.18) | 0.8 |
| Stage | Stage1 | 3 | Reference | |
| | Stage2 | 8 | 1.34 (0.04, 35.30) | 0.9 |
| | Stage3 | 9 | 0.30 (0.01, 6.55) | 0.5 |
| | Stage4 | 20 | 1.54 (0.05, 29.63) | 0.8 |
| Grade | Grade1 | 2 | Reference | |
| | Grade2 | 20 | 7.32 (0.11, 532.91) | 0.3 |
| | Grade3 | 18 | 13.08 (0.21, 969.79) | 0.2 |
| Radiotherapy | No | 6 | Reference | |
| | Yes | 34 | 0.60 (0.05, 4.78) | 0.6 |
| KLF6 | high | 20 | Reference | |
| | low | 20 | 2.15 (0.45, 11.84) | 0.3 |

| Variable | | N | Odds ratio | p |
|---|---|---|---|---|
| Age | <60 | 15 | Reference | |
| | >=60 | 25 | 0.04 (0.00, 0.56) | 0.053 |
| Gender | Male | 31 | Reference | |
| | Female | 9 | 11.02 (0.75, 325.58) | 0.105 |
| Stage | Stage1 | 3 | Reference | |
| | Stage2 | 8 | 146.35 (1.16, 75502.72) | 0.064 |
| | Stage3 | 9 | 0.82 (0.01, 64.15) | 0.927 |
| | Stage4 | 20 | 16.10 (0.30, 1555.54) | 0.174 |
| Grade | Grade1 | 2 | Reference | |
| | Grade2 | 20 | 3.41 (0.04, 284.83) | 0.560 |
| | Grade3 | 18 | 39.37 (0.46, 5918.03) | 0.107 |
| Radiotherapy | No | 6 | Reference | |
| | Yes | 34 | 0.08 (0.00, 1.59) | 0.130 |
| LAMA3 | high | 20 | Reference | |
| | low | 20 | 288.11 (10.76, 50692.81) | 0.007 |

## B R/M-HNSCC Cetuximab treatment (odds of short PFS)

| Variable | | N | Odds ratio | p |
|---|---|---|---|---|
| Age | <60 | 15 | Reference | |
| | >=60 | 25 | 0.25 (0.03, 1.37) | 0.14 |
| Gender | Male | 31 | Reference | |
| | Female | 9 | 2.77 (0.31, 36.53) | 0.38 |
| Stage | Stage1 | 3 | Reference | |
| | Stage2 | 8 | 1.14 (0.03, 34.81) | 0.94 |
| | Stage3 | 9 | 0.12 (0.00, 3.50) | 0.23 |
| | Stage4 | 20 | 0.53 (0.02, 12.70) | 0.70 |
| Grade | Grade1 | 2 | Reference | |
| | Grade2 | 20 | 17.89 (0.23, 1680.84) | 0.18 |
| | Grade3 | 18 | 32.03 (0.43, 3210.28) | 0.11 |
| Radiotherapy | No | 6 | Reference | |
| | Yes | 34 | 0.46 (0.04, 4.21) | 0.51 |
| LAMB3 | high | 20 | Reference | |
| | low | 20 | 5.16 (0.92, 40.21) | 0.08 |

| Variable | | N | Odds ratio | p |
|---|---|---|---|---|
| Age | <60 | 15 | Reference | |
| | >=60 | 25 | 0.27 (0.03, 1.69) | 0.18 |
| Gender | Male | 31 | Reference | |
| | Female | 9 | 1.84 (0.21, 19.76) | 0.59 |
| Stage | Stage1 | 3 | Reference | |
| | Stage2 | 8 | 3.06 (0.04, 305.19) | 0.60 |
| | Stage3 | 9 | 0.10 (0.00, 4.83) | 0.30 |
| | Stage4 | 20 | 2.22 (0.04, 151.90) | 0.69 |
| Grade | Grade1 | 2 | Reference | |
| | Grade2 | 20 | 3.49 (0.06, 245.32) | 0.53 |
| | Grade3 | 18 | 4.46 (0.08, 276.36) | 0.44 |
| Radiotherapy | No | 6 | Reference | |
| | Yes | 34 | 0.22 (0.01, 2.50) | 0.24 |
| LAMC2 | high | 20 | Reference | |
| | low | 20 | 24.32 (3.03, 664.87) | 0.01 |

| Variable | | N | Odds ratio | p |
|---|---|---|---|---|
| Age | <60 | 15 | Reference | |
| | >=60 | 25 | 0.37 (0.05, 2.06) | 0.27 |
| Gender | Male | 31 | Reference | |
| | Female | 9 | 3.86 (0.39, 60.89) | 0.27 |
| Stage | Stage1 | 3 | Reference | |
| | Stage2 | 8 | 1.90 (0.04, 73.73) | 0.72 |
| | Stage3 | 9 | 0.25 (0.00, 7.74) | 0.45 |
| | Stage4 | 20 | 1.33 (0.04, 37.32) | 0.87 |
| Grade | Grade1 | 2 | Reference | |
| | Grade2 | 20 | 4.80 (0.08, 365.35) | 0.44 |
| | Grade3 | 18 | 5.44 (0.10, 353.24) | 0.39 |
| Radiotherapy | No | 6 | Reference | |
| | Yes | 34 | 0.45 (0.03, 4.58) | 0.51 |
| MT2A | high | 20 | Reference | |
| | low | 20 | 9.60 (1.76, 75.32) | 0.02 |

| Variable | | N | Odds ratio | p |
|---|---|---|---|---|
| Age | <60 | 15 | Reference | |
| | >=60 | 25 | 0.18 (0.02, 1.03) | 0.07 |
| Gender | Male | 31 | Reference | |
| | Female | 9 | 3.07 (0.30, 46.67) | 0.37 |
| Stage | Stage1 | 3 | Reference | |
| | Stage2 | 8 | 5.03 (0.12, 216.57) | 0.36 |
| | Stage3 | 9 | 1.62 (0.04, 60.98) | 0.79 |
| | Stage4 | 20 | 7.74 (0.21, 271.95) | 0.23 |
| Grade | Grade1 | 2 | Reference | |
| | Grade2 | 20 | 14.97 (0.21, 2118.64) | 0.22 |
| | Grade3 | 18 | 19.50 (0.30, 2213.83) | 0.16 |
| Radiotherapy | No | 6 | Reference | |
| | Yes | 34 | 0.98 (0.07, 13.30) | 0.99 |
| PHLDA1 | high | 20 | Reference | |
| | low | 20 | 17.38 (2.53, 215.44) | 0.01 |

| Variable | | N | Odds ratio | p |
|---|---|---|---|---|
| Age | <60 | 15 | Reference | |
| | >=60 | 25 | 0.39 (0.06, 2.19) | 0.30 |
| Gender | Male | 31 | Reference | |
| | Female | 9 | 1.52 (0.17, 17.57) | 0.71 |
| Stage | Stage1 | 3 | Reference | |
| | Stage2 | 8 | 0.96 (0.03, 28.95) | 0.98 |
| | Stage3 | 9 | 0.18 (0.00, 4.85) | 0.31 |
| | Stage4 | 20 | 0.50 (0.02, 11.22) | 0.66 |
| Grade | Grade1 | 2 | Reference | |
| | Grade2 | 20 | 1.81 (0.03, 122.05) | 0.77 |
| | Grade3 | 18 | 2.79 (0.05, 202.14) | 0.61 |
| Radiotherapy | No | 6 | Reference | |
| | Yes | 34 | 0.35 (0.03, 3.35) | 0.38 |
| PLEK2 | high | 20 | Reference | |
| | low | 20 | 6.55 (1.25, 44.27) | 0.03 |

| Variable | | N | Odds ratio | p |
|---|---|---|---|---|
| Age | <60 | 15 | Reference | |
| | >=60 | 25 | 0.28 (0.04, 1.32) | 0.1 |
| Gender | Male | 31 | Reference | |
| | Female | 9 | 1.56 (0.19, 17.56) | 0.7 |
| Stage | Stage1 | 3 | Reference | |
| | Stage2 | 8 | 1.60 (0.05, 41.68) | 0.8 |
| | Stage3 | 9 | 0.27 (0.01, 5.33) | 0.4 |
| | Stage4 | 20 | 1.22 (0.04, 20.40) | 0.9 |
| Grade | Grade1 | 2 | Reference | |
| | Grade2 | 20 | 4.23 (0.07, 269.13) | 0.5 |
| | Grade3 | 18 | 7.17 (0.13, 432.20) | 0.3 |
| Radiotherapy | No | 6 | Reference | |
| | Yes | 34 | 0.54 (0.05, 4.40) | 0.6 |
| SERPINE1 | high | 20 | Reference | |
| | low | 20 | 1.32 (0.27, 6.85) | 0.7 |

| Variable | | N | Odds ratio | p |
|---|---|---|---|---|
| Age | <60 | 15 | Reference | |
| | >=60 | 25 | 0.29 (0.05, 1.35) | 0.1 |
| Gender | Male | 31 | Reference | |
| | Female | 9 | 1.46 (0.19, 15.61) | 0.7 |
| Stage | Stage1 | 3 | Reference | |
| | Stage2 | 8 | 1.57 (0.05, 38.43) | 0.8 |
| | Stage3 | 9 | 0.28 (0.01, 5.45) | 0.4 |
| | Stage4 | 20 | 1.29 (0.05, 21.04) | 0.9 |
| Grade | Grade1 | 2 | Reference | |
| | Grade2 | 20 | 5.21 (0.08, 351.64) | 0.4 |
| | Grade3 | 18 | 8.60 (0.14, 565.64) | 0.3 |
| Radiotherapy | No | 6 | Reference | |
| | Yes | 34 | 0.53 (0.04, 4.39) | 0.6 |
| SPHK1 | high | 20 | Reference | |
| | low | 20 | 1.20 (0.26, 5.72) | 0.8 |

| Variable | | N | Odds ratio | p |
|---|---|---|---|---|
| Age | <60 | 15 | Reference | |
| | >=60 | 25 | 0.17 (0.01, 1.24) | 0.12 |
| Gender | Female | 9 | Reference | |
| | Male | 31 | 0.17 (0.01, 2.08) | 0.19 |
| Stage | Stage1 | 3 | Reference | |
| | Stage2 | 8 | 1.25 (0.01, 203.33) | 0.92 |
| | Stage3 | 9 | 0.07 (0.00, 5.66) | 0.26 |
| | Stage4 | 20 | 0.27 (0.00, 18.08) | 0.57 |
| Grade | Grade1 | 2 | Reference | |
| | Grade2 | 20 | 1.84 (0.03, 134.57) | 0.76 |
| | Grade3 | 18 | 13.15 (0.20, 1189.21) | 0.22 |
| Radiotherapy | No | 6 | Reference | |
| | Yes | 34 | 0.12 (0.01, 1.85) | 0.15 |
| EREG | High | 20 | Reference | |
| | Low | 20 | 58.99 (4.59, 2987.17) | 0.01 |

| Variable | | N | Odds ratio | p |
|---|---|---|---|---|
| Age | <60 | 15 | Reference | |
| | >=60 | 25 | 0.24 (0.03, 1.26) | 0.12 |
| Gender | Female | 9 | Reference | |
| | Male | 31 | 0.42 (0.03, 4.04) | 0.48 |
| Stage | Stage1 | 3 | Reference | |
| | Stage2 | 8 | 0.84 (0.02, 24.64) | 0.92 |
| | Stage3 | 9 | 0.11 (0.00, 3.04) | 0.21 |
| | Stage4 | 20 | 0.56 (0.02, 12.26) | 0.71 |
| Grade | Grade1 | 2 | Reference | |
| | Grade2 | 20 | 11.69 (0.18, 1516.68) | 0.25 |
| | Grade3 | 18 | 10.35 (0.19, 920.27) | 0.25 |
| Radiotherapy | No | 6 | Reference | |
| | Yes | 34 | 0.35 (0.03, 2.88) | 0.34 |
| AREG | High | 20 | Reference | |
| | Low | 20 | 4.69 (0.86, 34.20) | 0.09 |

**Suppl. Figure 14: Correlation of fDEGs with Cetuximab response in R/M-HNSCC patients**
(**A**) Area under the curve (AUC) of receiver operating characteristic curves (ROC) for the association of the indicated genes with response to Cetuximab treatment in R/M-HNSCC patients (GSE65021) are shown in the upper graphs. Boxplot-whisker graphs are shown for the expression of the indicated gene with short progression-free survival (PFS; blue) and long-PFS (red) (displayed are: minimum, $1^{st}$ quartile, median, $3^{rd}$ quartile, maximum). (**B**) Multivariate linear regression model testing the odds to belong to the short progression-free survival (PFS) patient group. Shown are reference, p-values, odds with confidence interval for the parameters age, gender, stage, grade, radiotherapy, and fDEG (stratified at mean into high and low gene expression).
