# Supplementary material for: EGFR-mediated local invasiveness and response to Cetuximab in head and neck cancer
Source: Mol Cancer. 2025 Mar 22;24:94. doi: 10.1186/s12943-025-02290-1 (PMC11929204; doi:10.1186/s12943-025-02290-1)
Supplement: Supplementary file 7 — Supplementary Material 7. [file 12943_2025_2290_MOESM7_ESM.docx]

**fDEGs (n = 16) expression and response to Cetuximab in Bossi et al.**

Shown are odds of low progression-free survival of low versus high gene expression (median split; n = 40 patients), odds range (lower and higher values), and p-values.

| **#** | **Gene** | **Odds ratio**  **(short PFS)** | **Lower** | **Upper** | **p-value** |
| --- | --- | --- | --- | --- | --- |
| **1** | **COL17A1** | 34.68 | 4.35 | 856.04 | 0.005 |
| **2** | **FOSL1** | 24.55 | 3.27 | 441.69 | 0.008 |
| **3** | **IL8** | 2.45 | 0.53 | 12.77 | 0.3 |
| **4** | **INHBA** | 2.30 | 0.50 | 12.47 | 0.3 |
| **5** | **ITGA3** | 8.25 | 1.67 | 56.50 | 0.02 |
| **6** | **ITGA5** | 2.26 | 0.45 | 14.24 | 0.3 |
| **7** | **ITGB4** | 39.22 | 4.49 | 895.20 | 0.005 |
| **8** | **KLF6** | 2.15 | 0.45 | 11.84 | 0.3 |
| **9** | **LAMA3** | 288.11 | 10.76 | 50692.81 | 0.007 |
| **10** | **LAMB3** | 5.16 | 0.92 | 40.21 | 0.08 |
| **11** | **LAMC2** | 24.32 | 3.03 | 664.87 | 0.01 |
| **12** | **MT2A** | 9.60 | 1.76 | 75.32 | 0.02 |
| **13** | **PHLDA1** | 17.38 | 2.53 | 215.44 | 0.01 |
| **14** | **PLEK2** | 6.55 | 1.25 | 44.27 | 0.03 |
| **15** | **SERPINE1** | 1.32 | 0.27 | 6.85 | 0.7 |
| **16** | **SPHK1** | 1.20 | 0.26 | 5.72 | 0.8 |

**5-gene signature expression and response to Cetuximab in Bossi et al.**

Shown are odds of low progression-free survival of low versus high gene expression (median split; n = 40 patients), odds range (lower and higher values), and p-values.

| **#** | **Gene** | **Odds ratio**  **(short PFS)** | **Lower** | **Upper** | **p-value** |
| --- | --- | --- | --- | --- | --- |
| **1** | **DDIT4** | 2.77 | 0.50 | 17.98 | 0.3 |
| **2** | **FADD** | 8.18 | 1.31 | 68.62 | 0.03 |
| **3** | **ITGB4** | 39.22 | 4.49 | 895.20 | 0.005 |
| **4** | **NCEH1** | 1.96 | 0.40 | 11.22 | 0.4 |
| **5** | **TIMP1** | 2.05 | 0.37 | 14.26 | 0.4 |

**invGRN genes (n = 56) expression and response to Cetuximab in Bossi et al.**

Shown are odds of low progression-free survival of low versus high gene expression (median split; n = 40 patients), odds range (lower and higher values), and p-values.

| **#** | **Gene** | **Odds ratio**  **(short PFS)** | **Lower** | **Upper** | **p-value** |
| --- | --- | --- | --- | --- | --- |
| **1** | **AARS1** | 1.29 | 0.25 | 7.13 | 0.8 |
| **2** | **ABL2** | 10.05 | 1.54 | 111.97 | 0.03 |
| **3** | **ACTN1** | 4.48 | 0.85 | 30.82 | 0.09 |
| **4** | **ALDH3A1** | 2.06 | 0.45 | 10.48 | 0.4 |
| **5** | **AREG** | 4.69 | 0.86 | 34.20 | 0.09 |
| **6** | **ARHGAP29** | 0.33 | 0.04 | 1.98 | 0.25 |
| **7** | **ATP5MC2** | 2.48 | 0.52 | 14.23 | 0.27 |
| **8** | **CAP1** | 6.74 | 1.27 | 49.89 | 0.04 |
| **9** | **CAV1** | 78.69 | 7.22 | 2829.35 | 0.003 |
| **10** | **CAV2** | 27.10 | 2.87 | 658.49 | 0.01 |
| **11** | **CBX7** | 0.14 | 0.02 | 0.75 | 0.03 |
| **12** | **CCDC6** | 1.00 | 0.21 | 4.93 | 1.0 |
| **13** | **CDCP1** | 18.17 | 2.88 | 210.69 | 0.006 |
| **14** | **CLSTN1** | 4.75 | 0.83 | 43.10 | 0.1 |
| **15** | **DUSP6** | 1.49 | 0.30 | 7.68 | 0.6 |
| **16** | **ENO1** | 1.08 | 0.24 | 4.96 | 0.9 |
| **17** | **ERBB2** | 1.71 | 0.35 | 8.50 | 0.5 |
| **18** | **F3** | 16.38 | 2.57 | 164.96 | 0.007 |
| **19** | **FADS1** | 1.44 | 0.26 | 9.16 | 0.7 |
| **20** | **FLNA** | 6.46 | 1.02 | 67.38 | 0.07 |
| **21** | **FSTL3** | 32.97 | 4.43 | 512.85 | 0.003 |
| **22** | **GGT6** | 1.07 | 0.23 | 5.11 | 0.9 |
| **23** | **HINT1** | 3.07 | 0.66 | 17.08 | 0.2 |
| **24** | **HSPA12A** | 0.88 | 0.19 | 4.03 | 0.9 |
| **25** | **HSPH1** | 109.35 | 7.41 | 7960.65 | 0.006 |
| **26** | **IGF1R** | 16.66 | 2.28 | 206.84 | 0.01 |
| **27** | **IGF2BP2** | 3.73 | 0.77 | 22.16 | 0.1 |
| **28** | **INHBA** | 2.30 | 0.50 | 12.47 | 0.3 |
| **29** | **ITGA3** | 8.25 | 1.67 | 56.50 | 0.02 |
| **30** | **ITGA5** | 2.26 | 0.45 | 14.24 | 0.3 |
| **31** | **ITGB1** | 2.52 | 0.49 | 16.47 | 0.3 |
| **32** | **ITGB4** | 39.22 | 4.49 | 895.20 | 0.005 |
| **33** | **JAG1** | 3.55 | 0.81 | 18.71 | 0.1 |
| **34** | **KLF10** | 4.87 | 0.85 | 44.20 | 0.1 |
| **35** | **KPNA4** | 2.4 | 0.4 | 18.21 | 0.4 |
| **36** | **LAMB1** | 2.02 | 0.42 | 10.78 | 0.4 |
| **37** | **LAMB3** | 5.16 | 0.92 | 40.21 | 0.08 |
| **38** | **LAMC2** | 24.32 | 3.03 | 664.87 | 0.01 |
| **39** | **LRRC59** | 4.09 | 0.62 | 37.43 | 0.17 |
| **40** | **MACF1** | 10.66 | 1.96 | 98.73 | 0.01 |
| **41** | **MAP4K4** | 5.57 | 0.96 | 46.44 | 0.07 |
| **42** | **MLLT6** | 0.67 | 0.11 | 3.79 | 0.7 |
| **43** | **MSN** | 6.30 | 1.26 | 43.34 | 0.04 |
| **44** | **MUC20** | 0.47 | 0.07 | 2.58 | 0.4 |
| **45** | **MYH9** | 2.75 | 0.64 | 13.66 | 0.2 |
| **46** | **MYO1B** | 15.26 | 2.49 | 160.53 | 0.009 |
| **47** | **MYO10** | 1.96 | 0.33 | 14.01 | 0.5 |
| **48** | **NFIA** | 1.10 | 0.21 | 5.59 | 0.9 |
| **49** | **PKN** | 8.94 | 1.62 | 82.28 | 0.02 |
| **50** | **PTGS2** | 4.17 | 0.74 | 33.41 | 0.1 |
| **51** | **PXN** | 0.37 | 0.07 | 1.68 | 0.2 |
| **52** | **RNPC3** | 0.77 | 0.16 | 3.50 | 0.7 |
| **53** | **SNAI2** | 17.94 | 2.70 | 228.55 | 0.008 |
| **54** | **SPHK1** | 1.20 | 0.26 | 5.72 | 0.8 |
| **55** | **SSR3** | 0.7 | 0.12 | 3.77 | 0.7 |
| **56** | **TAPT1** | 0.25 | 0.04 | 1.15 | 0.09 |
| **57** | **TFDP2** | 1.10 | 0.24 | 5.17 | 0.9 |
| **58** | **TRIB1** | 1.34 | 0.28 | 6.36 | 0.7 |
| **59** | **WNK2** | 0.06 | 0.01 | 0.35 | 0.005 |
